# Supplementary material for: Evaluating the higher-order structure of the Profile of Emotional Competence (PEC): Confirmatory factor analysis and Bayesian structural equation modeling
Source: PLoS One. 2019 Nov 14;14(11):e0225070. doi: 10.1371/journal.pone.0225070 (PMC6855477; doi:10.1371/journal.pone.0225070)
Supplement: S1 Table — (PDF) [file pone.0225070.s001.pdf]

S1 Table. Results of the CFA with a robust maximum likelihood estimation of the competence-based structure model.

	Sample A: French-speaking Belgian	Sample B: Dutch-speaking Belgian	Sample C: Spanish	Sample D: Japanese
Identification				
Identification-self	.78* [.74, .81]	.83* [.81, .85]	.67* [.58, .75]	.76* [.68, .85]
Identification-other	.79* [.75, .82]	.84* [.82, .85]	.77* [.70, .85]	.75* [.67, .83]
Comprehension				
Comprehension-self	.69* [.66, .72]	.67* [.65, .69]	.58* [.51, .65]	.75* [.67, .83]
Comprehension-other	.87* [.84, .90]	.89* [.88, .91]	.81* [.71, .91]	.86* [.79, .94]
Expression				
Expression-self	.66* [.62, .71]	.72* [.70, .74]	.64* [.54, .74]	.76* [.61, .91]
Expression-other	.65* [.62, .69]	.77* [.75, .79]	.69* [.62, .76]	.69* [.55, .84]
Regulation				
Regulation-self	.60* [.57, .63]	.64* [.62, .65]	.53* [.47, .59]	.68* [.62, .75]
Regulation-other	.95* [.92, .99]	.95* [.93, .97]	.96* [.88, 1.03]	.90* [.84, .97]
Utilization				
Utilization-self	.52* [.47, .56]	.55* [.52, .58]	.54* [.45, .63]	.52* [.42, .62]
Utilization-other	.64* [.58, .70]	.65* [.61, .69]	.53* [.41, .65]	.91* [.81, 1.01]
Factor correlations				
Identification <-> Comprehension	1.20* [1.15, 1.25]	1.18* [1.16, 1.20]	1.39* [1.23, 1.55]	1.24* [1.11, 1.36]
Identification <-> Expression	1.17* [1.11, 1.22]	1.10* [1.08, 1.13]	1.27* [1.13, 1.40]	1.08* [.94, 1.22]
Identification <-> Regulation	.88* [.83, .92]	.86* [.83, .88]	.75* [.62, .88]	.84* [.73, .95]
Identification <-> Utilization	.86* [.78, .94]	.87* [.82, .91]	.78* [.60, .97]	.81* [.68, .94]
Comprehension <-> Expression	1.14* [1.08, 1.19]	1.07* [1.05, 1.10]	1.26* [1.12, 1.40]	.99* [.86, 1.11]
Comprehension <-> Regulation	.82* [.77, .86]	.82* [.79, .84]	.78* [.64, .92]	.84* [.74, .94]
Comprehension <-> Utilization	.77* [.70, .85]	.79* [.75, .83]	.85* [.65, 1.04]	.78* [.66, .91]
Expression <-> Regulation	1.06* [1.00, 1.12]	1.00* [.97, 1.03]	.98* [.86, 1.09]	1.03* [.85, 1.21]
Expression <-> Utilization	.89* [.80, .99]	.88* [.83, .93]	1.03* [.83, 1.23]	.88* [.67, 1.09]
Regulation <-> Utilization	1.00* [.93, 1.06]	1.00* [.96, 1.04]	1.16* [.99, 1.33]	1.13* [1.01, 1.24]

Note. 95% confidence intervals are in square brackets. EC: emotional competence.

*95% confidence interval does not include zero.
