# Supplementary material for: Evaluating the higher-order structure of the Profile of Emotional Competence (PEC): Confirmatory factor analysis and Bayesian structural equation modeling
Source: PLoS One. 2019 Nov 14;14(11):e0225070. doi: 10.1371/journal.pone.0225070 (PMC6855477; doi:10.1371/journal.pone.0225070)
Supplement: S2 Table — (PDF) [file pone.0225070.s002.pdf]

S2 Table. Results of the CFA with a robust maximum likelihood estimation of the hybrid structure model

	Sample A: French-speaking Belgian	Sample B: Dutch-speaking Belgian	Sample C: Spanish	Sample D: Japanese
Intrapersonal EC				
Identification-self	.96* [.93, .99]	.95* [.94, .97]	1.00* [1.00, 1.00]	.97* [.93, 1.01]
Comprehension-self	.89* [.86, .92]	.90* [.88, .91]	.83* [.76, .90]	.87* [.79, .95]
Expression-self	.77* [.73, .80]	.78* [.77, .80]	.71* [.63, .80]	.77* [.69, .86]
Regulation-self	.60* [.56, .64]	.62* [.59, .64]	.52* [.43, .62]	.65* [.56, .74]
Utilization-self	.30* [.24, .35]	.28* [.24, .32]	.37* [.26, .47]	.34* [.18, .50]
Interpersonal EC				
Identification-other	.92* [.90, .95]	.96* [.95, .97]	.92* [.84, .99]	.78* [.65, .90]
Comprehension-other	.91* [.89, .94]	.95* [.93, .96]	.95* [.88, 1.03]	.83* [.73, .93]
Expression-other	.76* [.73, .80]	.83* [.81, .84]	.85* [.79, .90]	.73* [.64, .81]
Regulation-other	.84* [.81, .86]	.82* [.80, .84]	.76* [.68, .84]	.93* [.87, 1.00]
Utilization-other	.48* [.43, .54]	.51* [.48, .54]	.35* [.23, .47]	.89* [.80, .97]
Identification				
Identification-self	-.26* [-.35, -.17]	.25* [.19, .31]	.00 [.00, .00]	.25* [.07, .42]
Identification-other	-.18* [-.24, -.12]	.15* [.11, .18]	.00 [.00, .00]	.23* [.05, .40]
Comprehension				
Comprehension-self	-.23* [-.28, -.18]	.19* [.16, .22]	.00 [-.01, .01]	.34* [.20, .49]
Comprehension-other	-.33* [-.41, -.26]	.30* [.25, .34]	.00 [-.01, .02]	.32* [.19, .45]
Expression				
Expression-self	.11 [-.01, .24]	.17* [.13, .22]	.08 [-.21, .38]	.29* [.03, .55]
Expression-other	.14 [-.01, .29]	.22* [.16, .28]	.13 [-.33, .59]	.23* [.04, .42]
Regulation				
Regulation-self	.40* [.36, .44]	.45* [.43, .47]	.42* [.35, .49]	.33* [.17, .48]
Regulation-other	.55* [.51, .59]	.56* [.53, .59]	.65* [.56, .74]	.36* [.19, .53]
Utilization				
Utilization-self	.31* [.22, .39]	.33* [.27, .38]	.34* [.17, .51]	.23* [.01, .45]
Utilization-other	.30* [.22, .38]	.30* [.25, .35]	.29* [.14, .44]	.27* [.02, .53]

Note. 95% confidence intervals are in square brackets. EC: emotional competence

*95% confidence interval does not include zero.
