# Supplementary material for: Evaluating the higher-order structure of the Profile of Emotional Competence (PEC): Confirmatory factor analysis and Bayesian structural equation modeling
Source: PLoS One. 2019 Nov 14;14(11):e0225070. doi: 10.1371/journal.pone.0225070 (PMC6855477; doi:10.1371/journal.pone.0225070)
Supplement: S3 Table — (PDF) [file pone.0225070.s003.pdf]

Table S3 Results of the Bayesian structural equation modeling of the modified two second-order factor model with cross-loadings and residual correlations

Sample A: French-speaking Belgian

First-order factor loadings	Identification-self	Comprehension-self	Expression-self	Regulation-self	Utilization-self	Identification-other	Comprehension-other	Expression-other	Regulation-other	Utilization-other
Item 6	**.39* [.19, .60]**	.02 [-.10, .15]	.00 [-.10, .10]	.03 [-.08, .14]	.08 [-.01, .16]	-.01 [-.12, .09]	.00 [-.10, .11]	.00 [-.10, .09]	.01 [-.11, .12]	.01 [-.07, .10]
Item 16	**.46* [.27, .66]**	-.01 [-.13, .11]	.01 [-.09, .10]	-.03 [-.14, .07]	.00 [-.08, .08]	.03 [-.07, .14]	.00 [-.10, .11]	.01 [-.08, .11]	.00 [-.11, .11]	-.02 [-.11, .06]
Item 20	**.52* [.32, .72]**	.05 [-.07, .17]	-.01 [-.10, .09]	-.03 [-.13, .08]	-.05 [-.13, .03]	-.01 [-.12, .10]	.01 [-.09, .12]	.00 [-.09, .10]	-.01 [-.12, .11]	.00 [-.09, .08]
Item 48	**.48* [.35, .65]**	.04 [-.08, .15]	.00 [-.09, .09]	.01 [-.09, .12]	.02 [-.06, .10]	-.02 [-.12, .09]	.01 [-.09, .11]	-.02 [-.12, .08]	-.02 [-.13, .09]	-.01 [-.09, .08]
Item 49	**.63* [.44, .82]**	.06 [-.06, .18]	.00 [-.09, .10]	-.03 [-.13, .08]	.01 [-.07, .09]	.01 [-.10, .11]	.01 [-.10, .11]	-.02 [-.11, .08]	-.02 [-.14, .10]	-.01 [-.09, .08]
Item 1	-.01 [-.11, .08]	**.69* [.56, .84]**	-.01 [-.11, .08]	-.01 [-.11, .10]	-.03 [-.11, .05]	.00 [-.10, .11]	.00 [-.10, .10]	-.01 [-.11, .08]	.01 [-.11, .13]	-.01 [-.09, .08]
Item 2	.03 [-.06, .12]	**.53* [.37, .69]**	.02 [-.07, .12]	.08 [-.02, .19]	-.05 [-.12, .03]	.03 [-.08, .13]	.01 [-.09, .11]	-.01 [-.10, .09]	.02 [-.09, .14]	-.02 [-.10, .06]
Item 10	.02 [-.07, .12]	**.21* [.05, .38]**	.01 [-.08, .11]	-.10 [-.21, .00]	.14* [.05, .22]	.02 [-.09, .12]	.01 [-.09, .12]	.03 [-.07, .12]	-.02 [-.13, .10]	.00 [-.08, .09]
Item 26	.01 [-.08, .10]	**.65* [.49, .80]**	-.03 [-.12, .07]	.07 [-.04, .17]	.00 [-.08, .08]	-.01 [-.12, .09]	-.02 [-.13, .08]	.00 [-.10, .09]	.00 [-.12, .12]	.00 [-.08, .09]
Item 43	.02 [-.07, .11]	**.62* [.46, .78]**	.01 [-.09, .10]	.03 [-.08, .13]	-.03 [-.11, .05]	-.02 [-.12, .09]	-.01 [-.11, .09]	.01 [-.09, .11]	-.01 [-.12, .11]	-.02 [-.11, .06]
Item 8	.00 [-.09, .10]	-.01 [-.12, .11]	**.61* [.46, .77]**	.04 [-.06, .14]	.03 [-.05, .11]	.00 [-.11, .10]	.01 [-.09, .11]	-.02 [-.11, .08]	.02 [-.10, .13]	.04 [-.05, .12]
Item 17	.01 [-.08, .10]	.03 [-.08, .14]	**.22* [.07, .38]**	.27* [.17, .38]	-.01 [-.09, .07]	-.01 [-.12, .09]	-.01 [-.11, .09]	-.01 [-.10, .09]	.08 [-.03, .20]	.02 [-.06, .11]
Item 25	-.01 [-.10, .08]	-.02 [-.13, .09]	**.72* [.57, .88]**	.00 [-.11, .11]	.00 [-.08, .08]	-.01 [-.11, .10]	.00 [-.10, .11]	.00 [-.10, .10]	.01 [-.11, .13]	.02 [-.07, .10]
Item 38	.01 [-.08, .09]	-.01 [-.12, .10]	**.55* [.44, .69]**	-.09 [-.20, .02]	.04 [-.04, .13]	.01 [-.09, .12]	.00 [-.10, .10]	.01 [-.09, .11]	-.02 [-.13, .10]	-.02 [-.10, .07]
Item 42	.01 [-.08, .10]	.04 [-.07, .16]	**.19* [.02, .36]**	.07 [-.04, .17]	.03 [-.06, .11]	.03 [-.07, .14]	.04 [-.06, .14]	.03 [-.07, .12]	.04 [-.07, .16]	.00 [-.09, .08]
Item 12	-.01 [-.11, .07]	-.04 [-.15, .07]	-.02 [-.11, .07]	**.80* [.70, .92]**	-.02 [-.10, .06]	-.01 [-.11, .09]	-.01 [-.11, .09]	-.01 [-.10, .09]	-.03 [-.15, .08]	.02 [-.07, .10]
Item 15	-.01 [-.10, .08]	-.01 [-.12, .10]	.02 [-.08, .11]	**.59* [.47, .72]**	.00 [-.08, .08]	.00 [-.11, .10]	.00 [-.10, .10]	-.02 [-.12, .08]	.02 [-.10, .14]	.02 [-.06, .11]
Item 37	.04 [-.05, .13]	.13* [.02, .24]	.06 [-.03, .15]	**.49* [.37, .61]**	-.07 [-.15, .00]	.01 [-.09, .12]	.01 [-.09, .11]	.01 [-.08, .10]	-.01 [-.12, .11]	.02 [-.06, .10]
Item 39	-.01 [-.10, .08]	-.01 [-.12, .09]	-.01 [-.11, .08]	**.67* [.54, .80]**	-.03 [-.11, .05]	.00 [-.10, .11]	.00 [-.10, .10]	.00 [-.10, .09]	.00 [-.12, .12]	-.03 [-.12, .05]
Item 50	.01 [-.08, .10]	.03 [-.08, .14]	.01 [-.09, .10]	**.45* [.32, .58]**	.08 [.00, .16]	.00 [-.11, .10]	.01 [-.09, .11]	.00 [-.10, .10]	-.01 [-.12, .11]	.01 [-.08, .10]
Item 9	-.02 [-.11, .07]	-.04 [-.14, .07]	.00 [-.09, .09]	-.17* [-.27, -.06]	**.40* [.30, .49]**	.02 [-.08, .13]	.02 [-.08, .12]	.04 [-.05, .14]	-.02 [-.13, .10]	-.01 [-.10, .07]
Item 21	.00 [-.09, .10]	.01 [-.10, .12]	.01 [-.09, .10]	.07 [-.04, .17]	**.65* [.59, .72]**	-.02 [-.13, .08]	-.02 [-.12, .09]	.00 [-.10, .10]	.00 [-.11, .12]	.01 [-.08, .09]
Item 22	.00 [-.09, .09]	-.01 [-.11, .10]	.00 [-.09, .09]	.02 [-.08, .12]	**.31* [.21, .41]**	.06 [-.04, .17]	.04 [-.06, .15]	.04 [-.06, .14]	.06 [-.06, .17]	.05 [-.04, .13]
Item 24	-.01 [-.10, .08]	-.02 [-.13, .09]	.00 [-.09, .10]	.00 [-.11, .11]	**.66* [.57, .75]**	.01 [-.10, .11]	.01 [-.09, .11]	.00 [-.10, .10]	.02 [-.10, .13]	-.01 [-.09, .08]
Item 41	.02 [-.06, .12]	.04 [-.07, .15]	.01 [-.08, .11]	-.05 [-.16, .05]	**.57* [.47, .66]**	.03 [-.07, .14]	.03 [-.07, .14]	.02 [-.07, .12]	.01 [-.10, .13]	-.02 [-.11, .07]
Item 7	.01 [-.08, .10]	-.02 [-.13, .08]	-.03 [-.12, .07]	.01 [-.09, .11]	.01 [-.07, .09]	**.56* [.37, .74]**	-.01 [-.12, .10]	.00 [-.10, .10]	-.02 [-.14, .10]	.00 [-.09, .08]
Item 29	.01 [-.08, .10]	.01 [-.10, .12]	.00 [-.09, .09]	.01 [-.09, .11]	-.01 [-.09, .07]	**.61* [.47, .77]**	.01 [-.09, .11]	.01 [-.08, .11]	.01 [-.11, .13]	-.02 [-.11, .06]
Item 30	-.02 [-.11, .07]	-.02 [-.12, .08]	.01 [-.08, .10]	-.02 [-.11, .08]	.04 [-.04, .11]	**.59* [.42, .76]**	.04 [-.07, .15]	.03 [-.07, .12]	.04 [-.08, .16]	.02 [-.06, .10]
Item 40	.01 [-.08, .10]	.04 [-.07, .15]	.00 [-.09, .09]	.00 [-.10, .10]	-.03 [-.11, .05]	**.54* [.35, .72]**	.01 [-.10, .12]	-.04 [-.14, .06]	-.02 [-.15, .10]	-.01 [-.09, .08]
Item 44	.00 [-.09, .09]	.04 [-.07, .14]	-.02 [-.11, .07]	-.03 [-.13, .07]	.00 [-.08, .08]	**.70* [.52, .88]**	.01 [-.09, .12]	-.01 [-.11, .08]	-.02 [-.15, .10]	-.02 [-.10, .07]
Item 5	.00 [-.09, .09]	.00 [-.11, .11]	.01 [-.08, .10]	-.04 [-.14, .07]	.03 [-.05, .11]	.02 [-.09, .13]	**.57* [.37, .77]**	.02 [-.08, .12]	-.01 [-.13, .11]	-.01 [-.09, .07]
Item 13	.00 [-.09, .09]	-.02 [-.12, .09]	.01 [-.08, .10]	.01 [-.09, .11]	.03 [-.05, .11]	.02 [-.09, .12]	**.56* [.42, .73]**	-.03 [-.13, .07]	.01 [-.11, .13]	.03 [-.05, .11]

Item 14	-.01 [-.10, .08]	-.04 [-.15, .07]	-.02 [-.11, .07]	-.02 [-.12, .08]	.04 [-.04, .12]	.03 [-.08, .14]	**.57* [.36, .78]**	.00 [-.10, .10]	.00 [-.12, .13]	.01 [-.08, .09]
Item 18	.02 [-.06, .12]	.05 [-.06, .16]	.04 [-.05, .14]	.04 [-.05, .14]	.03 [-.05, .11]	.01 [-.10, .12]	**.38* [.17, .59]**	-.02 [-.12, .08]	-.02 [-.14, .10]	.02 [-.06, .11]
Item 34	.01 [-.08, .10]	.01 [-.10, .12]	.00 [-.10, .09]	-.06 [-.16, .05]	.03 [-.05, .11]	.03 [-.08, .14]	**.54* [.33, .74]**	.04 [-.06, .14]	-.02 [-.14, .10]	-.05 [-.13, .04]
Item 23	-.01 [-.10, .07]	-.03 [-.13, .08]	-.03 [-.12, .06]	-.03 [-.14, .07]	.02 [-.06, .10]	.02 [-.09, .12]	-.01 [-.11, .10]	**.66* [.52, .80]**	.03 [-.09, .15]	-.01 [-.09, .08]
Item 28	-.01 [-.10, .08]	.01 [-.10, .11]	-.01 [-.10, .08]	.02 [-.08, .12]	-.05 [-.13, .03]	.00 [-.11, .10]	-.01 [-.11, .10]	**.51* [.37, .67]**	.02 [-.10, .14]	-.07 [-.16, .02]
Item 31	.00 [-.09, .09]	-.01 [-.11, .10]	.00 [-.09, .09]	.00 [-.10, .10]	.01 [-.07, .09]	-.03 [-.13, .07]	.00 [-.11, .10]	**.61* [.51, .75]**	-.02 [-.13, .10]	-.05 [-.13, .03]
Item 45	-.01 [-.10, .07]	-.02 [-.13, .08]	-.01 [-.11, .08]	.00 [-.10, .10]	.03 [-.05, .11]	.00 [-.10, .11]	.00 [-.10, .11]	**.64* [.50, .78]**	.04 [-.08, .16]	-.03 [-.11, .05]
Item 46	.01 [-.07, .10]	.04 [-.06, .15]	.03 [-.06, .13]	.02 [-.08, .12]	-.01 [-.09, .07]	.02 [-.09, .13]	.03 [-.07, .13]	**.35* [.21, .50]**	.03 [-.09, .15]	.03 [-.06, .11]
Item 19	-.01 [-.10, .08]	.00 [-.11, .11]	-.02 [-.11, .07]	.01 [-.09, .11]	-.02 [-.09, .06]	-.02 [-.14, .08]	-.02 [-.12, .09]	-.01 [-.12, .08]	**.70* [.56, .85]**	.00 [-.09, .08]
Item 27	.02 [-.07, .11]	.04 [-.07, .14]	.04 [-.05, .13]	-.07 [-.18, .03]	.01 [-.06, .09]	.04 [-.06, .16]	.03 [-.07, .14]	.12* [.02, .23]	**.40* [.20, .59]**	-.03 [-.11, .06]
Item 33	-.01 [-.10, .08]	-.03 [-.13, .08]	.01 [-.08, .10]	-.05 [-.16, .05]	.01 [-.07, .09]	.02 [-.09, .12]	.01 [-.09, .12]	.14* [.05, .24]	**.59* [.41, .77]**	.04 [-.04, .13]
Item 35	.00 [-.09, .09]	.01 [-.09, .12]	.01 [-.08, .10]	-.03 [-.13, .08]	.05 [-.03, .12]	.01 [-.10, .12]	.01 [-.10, .11]	-.01 [-.11, .09]	**.37* [.18, .56]**	.19* [.11, .29]
Item 47	.01 [-.08, .10]	.00 [-.11, .10]	.00 [-.09, .09]	.10 [-.01, .21]	-.01 [-.09, .06]	-.02 [-.13, .09]	-.02 [-.13, .08]	-.04 [-.14, .06]	**.52* [.33, .71]**	.07 [-.02, .15]
Item 3	-.01 [-.10, .07]	-.03 [-.13, .08]	-.01 [-.10, .07]	.03 [-.07, .13]	.01 [-.07, .09]	-.01 [-.12, .09]	-.01 [-.11, .09]	-.04 [-.13, .06]	.01 [-.11, .12]	**.63* [.56, .73]**
Item 4	.01 [-.08, .10]	.02 [-.08, .13]	.02 [-.07, .11]	.06 [-.04, .17]	-.02 [-.10, .07]	.00 [-.10, .11]	.01 [-.09, .11]	.00 [-.10, .10]	.02 [-.09, .14]	**.59* [.48, .70]**
Item 11	.01 [-.08, .10]	.01 [-.10, .12]	.01 [-.08, .11]	.07 [-.03, .18]	-.01 [-.09, .08]	.01 [-.10, .11]	-.01 [-.11, .10]	-.01 [-.11, .09]	.02 [-.10, .14]	**.59* [.47, .70]**
Item 32	.02 [-.06, .11]	.01 [-.09, .11]	.04 [-.05, .13]	.08 [-.02, .18]	-.01 [-.08, .07]	.07 [-.03, .17]	.04 [-.06, .15]	.06 [-.03, .16]	.15* [.04, .28]	**.29* [.18, .39]**
Item 36	-.01 [-.10, .08]	.00 [-.10, .11]	-.01 [-.10, .08]	-.03 [-.13, .07]	.00 [-.08, .08]	-.03 [-.14, .07]	-.01 [-.12, .09]	-.06 [-.15, .04]	-.02 [-.13, .10]	**.52* [.41, .63]**
Second-order factor loadings	Intrapersonal EC	Interpersonal EC								
Identification-self	**.96* [.75, 1.19]**	-.03 [-.35, .26]								
Comprehension-self	**.90* [.73, 1.08]**	-.04 [-.29, .18]								
Expression-self	**.69* [.48, .91]**	.13 [-.15, .37]								
Regulation-self	**.69* [.50, .86]**	-.05 [-.26, .15]								
Utilization-self	**.14 [-.13, .39]**	.25* [.03, .44]								
Identification-other	.02 [-.21, .23]	**.90* [.74, 1.07]**								
Comprehension-other	.07 [-.19, .29]	**.87* [.69, 1.06]**								
Expression-other	-.14 [-.40, .09]	**.88* [.71, 1.07]**								
Regulation-other	.03 [-.20, .24]	**.85* [.67, 1.01]**								
Utilization-other	.09 [-.14, .30]	**.40* [.14, .63]**								
Factor correlation	Interpersonal EC									
Intrapersonal EC	**.73 [.50, .87]**									
Residual correlation	Identification-self	Comprehension-self	Expression-self	Regulation-self	Utilization-self	Identification-other	Comprehension-other	Expression-other	Regulation-other	Utilization-other
Comprehension-self	.05 [-.08, .18]									
Expression-self	-.01 [-.14, .13]	-.01 [-.14, .12]								
Regulation-self	-.01 [-.15, .12]	.03 [-.10, .16]	.03 [-.10, .16]							
Utilization-self	.01 [-.12, .13]	-.02 [-.14, .10]	.02 [-.10, .14]	-.05 [-.16, .07]						

Identification-other	**.10 [-.03, .23]**	.03 [-.10, .16]	-.02 [-.15, .11]	-.01 [-.15, .12]	.01 [-.12, .13]					
Comprehension-other	.03 [-.11, .16]	**.27* [.14, .39]**	.01 [-.12, .14]	-.01 [-.15, .12]	.03 [-.10, .16]	.05 [-.08, .19]				
Expression-other	.00 [-.14, .13]	.01 [-.12, .13]	**.01 [-.12, .14]**	-.01 [-.14, .12]	.01 [-.10, .13]	.00 [-.13, .13]	.01 [-.12, .13]			
Regulation-other	-.01 [-.15, .12]	.01 [-.12, .14]	.03 [-.10, .16]	**.55* [.45, .64]**	-.02 [-.14, .10]	-.02 [-.15, .11]	-.02 [-.15, .11]	.06 [-.08, .19]		
Utilization-other	-.01 [-.15, .12]	-.02 [-.14, .11]	.03 [-.10, .15]	.09 [-.04, .21]	**.10 [-.01, .21]**	-.02 [-.14, .11]	-.01 [-.14, .12]	-.07 [-.19, .06]	.10 [-.03, .23]	
	Item 1	Item 2	Item 3	Item 4	Item 5	Item 6	Item 7	Item 8	Item 9	Item 10
Item 2	.10* [.01, .18]									
Item 3	-.05 [-.13, .04]	-.06 [-.13, .03]								
Item 4	-.08 [-.16, .01]	-.05 [-.13, .03]	.07 [-.02, .16]							
Item 5	.09* [.01, .16]	.07 [.00, .14]	-.05 [-.13, .03]	-.05 [-.13, .02]						
Item 6	-.02 [-.08, .05]	-.07* [-.13, -.01]	.02 [-.05, .09]	.02 [-.05, .09]	-.04 [-.09, .02]					
Item 7	-.07* [-.14, -.01]	-.07* [-.13, -.01]	.03 [-.04, .10]	.04 [-.03, .12]	-.06* [-.12, -.01]	.07* [.02, .12]				
Item 8	-.06 [-.13, .02]	-.04 [-.11, .03]	.04 [-.04, .12]	.07 [-.01, .15]	-.04 [-.11, .03]	.05 [-.01, .11]	.03 [-.04, .09]			
Item 9	.04 [-.03, .10]	.02 [-.05, .09]	-.02 [-.09, .06]	-.10* [-.17, -.02]	.14* [.08, .20]	-.02 [-.08, .03]	-.08* [-.13, -.02]	-.03 [-.10, .04]		
Item 10	.01 [-.07, .08]	-.13* [-.19, -.07]	.01 [-.06, .08]	.01 [-.05, .08]	-.03 [-.08, .02]	.14* [.10, .19]	.07* [.02, .12]	.02 [-.04, .08]	.04 [-.01, .09]	
Item 11	-.04 [-.12, .04]	-.04 [-.12, .04]	.10* [.01, .18]	.14* [.04, .23]	-.08* [-.15, -.01]	.04 [-.02, .11]	.02 [-.05, .09]	.02 [-.06, .10]	-.08* [-.15, -.01]	-.03 [-.09, .04]
Item 12	-.05 [-.14, .04]	.00 [-.09, .10]	.05 [-.05, .15]	.07 [-.03, .17]	-.05 [-.14, .04]	.02 [-.06, .10]	.03 [-.05, .12]	.04 [-.05, .13]	-.09* [-.17, .00]	-.05 [-.13, .03]
Item 13	-.07* [-.14, .00]	-.05 [-.11, .01]	.07 [.00, .15]	.05 [-.03, .12]	-.05 [-.11, .01]	.06* [.01, .12]	.07* [.01, .13]	.11* [.05, .18]	-.05 [-.11, .01]	.06* [.01, .11]
Item 14	-.03 [-.10, .04]	-.04 [-.11, .02]	.07 [.00, .15]	.02 [-.05, .10]	-.03 [-.10, .04]	.02 [-.04, .07]	.10* [.04, .15]	.02 [-.05, .08]	-.04 [-.10, .02]	.03 [-.02, .08]
Item 15	-.07 [-.14, .01]	-.04 [-.11, .04]	.06 [-.02, .14]	.05 [-.03, .14]	-.05 [-.11, .02]	.03 [-.03, .10]	.02 [-.04, .09]	.04 [-.04, .11]	-.09* [-.16, -.03]	-.04 [-.10, .02]
Item 16	-.08* [-.14, -.01]	-.08* [-.14, -.02]	-.01 [-.08, .06]	.05 [-.02, .12]	-.09* [-.14, -.03]	.10* [.04, .16]	.14* [.09, .19]	.10* [.04, .16]	-.08* [-.13, -.02]	.14* [.10, .19]
Item 17	-.01 [-.08, .05]	.07* [.01, .13]	.00 [-.07, .08]	.04 [-.04, .11]	-.07* [-.12, -.01]	.01 [-.04, .06]	-.01 [-.06, .05]	.02 [-.06, .09]	-.08* [-.13, -.02]	-.03 [-.07, .02]
Item 18	.13* [.06, .19]	.11* [.05, .17]	-.05 [-.12, .02]	-.04 [-.11, .02]	.15* [.08, .21]	-.08* [-.13, -.04]	-.08* [-.13, -.03]	-.07* [-.13, -.01]	.11* [.05, .16]	-.08* [-.12, -.04]
Item 19	-.04 [-.11, .04]	-.05 [-.12, .02]	.08 [-.01, .16]	.05 [-.03, .13]	-.04 [-.11, .02]	.03 [-.03, .09]	.04 [-.02, .10]	.01 [-.07, .08]	-.05 [-.11, .02]	.01 [-.05, .07]
Item 20	.09* [.01, .16]	.12* [.05, .18]	-.06 [-.13, .02]	-.05 [-.12, .03]	.06* [.00, .12]	-.06 [-.12, .01]	-.05 [-.10, .01]	-.07* [-.13, .00]	.03 [-.03, .08]	-.05 [-.10, .01]
Item 21	-.01 [-.10, .07]	-.10* [-.17, -.01]	.06 [-.03, .15]	.04 [-.05, .13]	-.04 [-.11, .04]	.07 [-.01, .14]	.02 [-.06, .09]	.05 [-.04, .13]	.05 [-.03, .13]	.09* [.02, .15]
Item 22	-.02 [-.09, .04]	-.04 [-.10, .02]	.07 [.00, .14]	.03 [-.05, .10]	.00 [-.05, .06]	.04 [-.01, .09]	.04 [-.01, .09]	-.02 [-.08, .04]	.02 [-.04, .08]	.05* [.00, .10]
Item 23	-.06 [-.14, .02]	-.08 [-.15, .00]	.00 [-.09, .08]	.05 [-.04, .13]	-.09* [-.16, -.02]	.02 [-.04, .09]	.06 [-.01, .13]	.00 [-.07, .08]	-.01 [-.08, .07]	.03 [-.03, .10]
Item 24	-.08 [-.17, .00]	-.07 [-.16, .01]	.02 [-.07, .12]	.04 [-.06, .13]	-.04 [-.12, .04]	.05 [-.03, .12]	.08* [.00, .15]	.04 [-.04, .13]	.00 [-.08, .08]	.11* [.04, .18]
Item 25	.06 [-.02, .15]	.04 [-.05, .12]	-.04 [-.13, .05]	-.03 [-.11, .06]	.10* [.02, .17]	-.04 [-.10, .03]	-.10* [-.17, -.03]	.07 [-.03, .16]	.08* [.01, .16]	-.01 [-.07, .06]
Item 26	.09* [.01, .18]	.10* [.01, .18]	-.02 [-.10, .07]	-.03 [-.12, .05]	.04 [-.03, .11]	-.01 [-.07, .05]	-.06 [-.13, .00]	-.05 [-.12, .03]	.01 [-.06, .08]	-.04 [-.11, .03]
Item 27	.05 [-.02, .12]	.00 [-.06, .07]	-.10* [-.17, -.02]	-.07 [-.14, .00]	.07* [.01, .13]	.00 [-.05, .05]	-.04 [-.09, .02]	.02 [-.04, .09]	.08* [.02, .13]	.03 [-.02, .08]
Item 28	.03 [-.04, .10]	.07* [.00, .13]	-.12* [-.19, -.05]	-.07 [-.14, .01]	.10* [.03, .16]	-.05 [-.10, .01]	-.04 [-.10, .01]	-.05 [-.11, .02]	.05 [-.01, .11]	-.01 [-.06, .04]
Item 29	.05 [-.03, .12]	.12* [.05, .19]	-.07 [-.15, .01]	-.07 [-.14, .01]	.09* [.02, .15]	-.08* [-.13, -.02]	-.06* [-.13, .00]	-.07* [-.14, .00]	.09* [.03, .15]	-.07* [-.12, -.02]
Item 30	-.07 [-.14, .01]	-.07 [-.14, .00]	.07 [-.02, .15]	.06 [-.02, .14]	-.03 [-.10, .03]	.01 [-.05, .07]	.16* [.09, .23]	.08* [.01, .15]	-.05 [-.11, .02]	.05 [.00, .11]
Item 31	.04 [-.03, .12]	.00 [-.07, .07]	-.13* [-.20, -.05]	-.08* [-.16, .00]	.14* [.08, .21]	-.02 [-.08, .04]	-.04 [-.10, .02]	-.05 [-.12, .02]	.07* [.01, .14]	.00 [-.06, .05]
Item 32	-.08* [-.15, -.01]	-.10* [-.16, -.03]	.02 [-.06, .11]	.15* [.07, .23]	-.10* [-.15, -.04]	.08* [.02, .13]	.09* [.03, .14]	.08* [.02, .15]	-.07* [-.13, -.01]	-.01 [-.06, .05]

Item 33	-.07 [-.15, .01]	-.08 [-.15, .00]	.03 [-.06, .12]	.07 [-.01, .16]	-.09* [-.16, -.02]	.03 [-.04, .10]	.04 [-.03, .11]	.06 [-.02, .14]	-.07 [-.14, .00]	-.01 [-.07, .05]
Item 34	.09* [.02, .16]	.05 [-.02, .12]	-.07 [-.14, .01]	-.10* [-.17, -.03]	.14* [.06, .21]	-.04 [-.09, .01]	-.04 [-.10, .01]	-.06 [-.12, .01]	.12* [.06, .18]	.03 [-.02, .08]
Item 35	-.06 [-.13, .01]	-.06 [-.12, .01]	.17* [.10, .24]	.13* [.05, .20]	-.08* [-.13, -.02]	.03 [-.02, .08]	.05 [-.01, .11]	.06 [-.01, .12]	-.05 [-.11, .01]	.00 [-.05, .05]
Item 36	.01 [-.07, .08]	-.05 [-.11, .02]	.35* [.28, .41]	-.01 [-.10, .07]	-.04 [-.10, .03]	-.01 [-.07, .05]	.03 [-.03, .09]	.02 [-.05, .09]	.04 [-.02, .11]	.04 [-.02, .10]
Item 37	.10* [.02, .17]	.14* [.07, .21]	-.02 [-.10, .07]	-.01 [-.09, .08]	-.04 [-.10, .03]	-.03 [-.09, .04]	-.04 [-.11, .02]	-.02 [-.09, .05]	-.06 [-.13, .01]	-.10* [-.15, -.04]
Item 38	.00 [-.07, .08]	.00 [-.06, .07]	-.03 [-.10, .05]	-.06 [-.14, .02]	.02 [-.04, .08]	-.02 [-.07, .04]	-.04 [-.10, .02]	.07 [.00, .13]	.09* [.03, .15]	.02 [-.03, .07]
Item 39	.00 [-.08, .08]	.01 [-.07, .09]	.00 [-.09, .08]	.03 [-.06, .12]	-.05 [-.13, .02]	.00 [-.07, .07]	.05 [-.02, .12]	-.01 [-.09, .07]	-.09* [-.16, -.01]	.00 [-.07, .06]
Item 40	.09* [.02, .16]	.10* [.04, .17]	-.03 [-.10, .04]	-.09* [-.15, -.01]	.13* [.07, .18]	-.07* [-.12, -.02]	-.03 [-.09, .03]	-.07* [-.13, -.01]	.06* [.01, .12]	-.05* [-.10, -.01]
Item 41	-.06 [-.14, .03]	-.05 [-.12, .03]	.01 [-.08, .10]	.01 [-.08, .09]	-.03 [-.10, .04]	.12* [.06, .19]	.08* [.02, .15]	.06 [-.02, .13]	.01 [-.07, .08]	.09* [.03, .15]
Item 42	.09* [.03, .15]	.11* [.05, .17]	-.09* [-.15, -.02]	-.04 [-.10, .03]	.08* [.03, .13]	-.04 [-.08, .01]	-.06* [-.11, -.01]	-.09* [-.16, -.02]	.03 [-.02, .09]	-.06* [-.10, -.01]
Item 43	.08 [.00, .16]	.03 [-.05, .11]	-.07 [-.15, .01]	-.06 [-.14, .03]	.06 [-.01, .13]	.00 [-.06, .06]	-.08* [-.14, -.01]	-.02 [-.09, .05]	.03 [-.04, .10]	.01 [-.06, .07]
Item 44	.08* [.00, .16]	.08* [.01, .15]	-.02 [-.11, .06]	-.06 [-.14, .02]	.11* [.04, .18]	-.06 [-.11, .00]	-.02 [-.09, .06]	-.07 [-.14, .01]	.06 [.00, .13]	-.04 [-.09, .02]
Item 45	-.06 [-.14, .02]	-.06 [-.14, .02]	.00 [-.09, .09]	.02 [-.06, .11]	-.07* [-.14, .00]	.04 [-.02, .11]	.05 [-.02, .12]	.02 [-.05, .10]	-.02 [-.09, .05]	.04 [-.02, .10]
Item 46	.06 [-.01, .12]	.07* [.01, .14]	-.05 [-.12, .02]	-.05 [-.12, .02]	.13* [.07, .19]	-.04 [-.09, .01]	-.10* [-.15, -.05]	-.01 [-.07, .06]	.11* [.06, .17]	.00 [-.05, .04]
Item 47	-.07 [-.14, .00]	-.06 [-.12, .01]	.08* [.00, .15]	.08* [.01, .16]	-.09* [-.15, -.03]	.03 [-.02, .09]	.02 [-.04, .08]	.04 [-.03, .11]	-.10* [-.16, -.04]	.00 [-.05, .05]
Item 48	-.03 [-.10, .03]	-.05 [-.11, .02]	-.01 [-.08, .07]	.02 [-.05, .10]	-.06 [-.11, .00]	.10* [.04, .16]	.11* [.06, .16]	.08* [.01, .14]	-.05 [-.11, .00]	.15* [.11, .20]
Item 49	.11* [.02, .18]	.14* [.07, .21]	-.05 [-.14, .03]	-.07 [-.14, .02]	.11* [.04, .18]	-.05 [-.12, .03]	-.05 [-.11, .01]	-.02 [-.10, .05]	.09* [.03, .15]	-.01 [-.07, .05]
Item 50	.01 [-.06, .07]	.00 [-.07, .06]	.04 [-.03, .12]	.01 [-.06, .09]	-.03 [-.09, .03]	.05 [.00, .10]	.03 [-.03, .09]	.03 [-.04, .09]	-.09* [-.15, -.04]	.00 [-.05, .05]
	Item 11	Item 12	Item 13	Item 14	Item 15	Item 16	Item 17	Item 18	Item 19	Item 20
Item 12	.05 [-.05, .15]									
Item 13	.06 [-.02, .13]	.04 [-.05, .12]								
Item 14	.04 [-.04, .11]	.03 [-.06, .11]	.14* [.07, .20]							
Item 15	.08* [.00, .16]	.09 [.00, .18]	.01 [-.06, .08]	.02 [-.04, .09]						
Item 16	.02 [-.05, .09]	.03 [-.06, .11]	.08* [.03, .14]	.04 [-.01, .10]	.02 [-.04, .08]					
Item 17	.05 [-.02, .12]	.13* [.04, .21]	-.01 [-.07, .04]	.01 [-.05, .07]	.03 [-.03, .10]	.00 [-.06, .05]				
Item 18	-.04 [-.11, .03]	-.01 [-.09, .07]	-.04 [-.09, .02]	-.05 [-.11, .01]	-.07* [-.13, -.01]	-.14* [-.18, -.09]	.02 [-.03, .08]			
Item 19	.04 [-.04, .12]	.06 [-.03, .15]	.06 [.00, .13]	.06 [-.01, .13]	.08* [.01, .15]	.04 [-.03, .10]	.05 [-.01, .11]	-.10* [-.16, -.04]		
Item 20	-.05 [-.12, .02]	-.03 [-.12, .06]	-.06 [-.11, .00]	-.02 [-.08, .04]	-.07* [-.13, .00]	-.06* [-.13, .00]	.01 [-.05, .06]	.10* [.05, .15]	-.07 [-.13, .00]	
Item 21	.05 [-.04, .14]	.03 [-.07, .13]	.04 [-.04, .12]	.01 [-.06, .09]	.04 [-.04, .12]	.06 [-.01, .13]	.02 [-.06, .09]	-.06 [-.13, .02]	.04 [-.05, .12]	-.09* [-.16, -.01]
Item 22	-.03 [-.10, .04]	.03 [-.05, .11]	.05 [.00, .11]	.04 [-.01, .10]	.00 [-.06, .06]	-.05 [-.10, .00]	.05 [.00, .10]	.02 [-.03, .07]	.00 [-.06, .06]	-.01 [-.07, .04]
Item 23	.02 [-.07, .10]	-.01 [-.11, .08]	-.01 [-.08, .07]	.03 [-.05, .10]	.02 [-.06, .10]	.07* [.00, .13]	-.02 [-.09, .05]	-.10* [-.16, -.03]	.07 [-.01, .15]	-.06 [-.12, .02]
Item 24	.02 [-.07, .11]	.00 [-.10, .10]	.06 [-.01, .14]	.07 [-.01, .15]	.03 [-.06, .11]	.06 [-.02, .13]	.00 [-.08, .07]	-.06 [-.13, .02]	.02 [-.07, .10]	-.05 [-.13, .03]
Item 25	-.03 [-.12, .05]	-.07 [-.16, .03]	-.05 [-.12, .02]	-.05 [-.12, .03]	.01 [-.07, .09]	-.05 [-.12, .02]	-.05 [-.14, .03]	.08* [.01, .15]	-.04 [-.12, .04]	.06 [-.02, .14]
Item 26	-.06 [-.14, .02]	-.01 [-.11, .08]	-.06 [-.13, .00]	-.08* [-.15, -.02]	-.01 [-.09, .07]	-.05 [-.11, .02]	-.04 [-.11, .03]	.05 [-.01, .12]	-.01 [-.09, .06]	.11* [.04, .18]
Item 27	-.06 [-.13, .01]	-.05 [-.14, .03]	-.05 [-.10, .01]	-.06* [-.12, -.01]	-.08* [-.15, -.02]	-.02 [-.07, .03]	-.05 [-.10, .01]	.02 [-.04, .07]	-.04 [-.11, .03]	.06* [.01, .12]
Item 28	-.08* [-.15, .00]	-.07 [-.15, .02]	-.07* [-.13, -.02]	-.06* [-.12, .00]	-.07 [-.13, .00]	-.01 [-.06, .05]	-.02 [-.08, .04]	.03 [-.02, .09]	-.03 [-.10, .03]	.06* [.01, .12]
Item 29	-.03 [-.10, .05]	-.04 [-.13, .05]	-.07* [-.13, -.01]	.01 [-.06, .07]	-.06 [-.13, .01]	-.06 [-.11, .00]	.00 [-.06, .06]	.12* [.06, .18]	-.04 [-.11, .03]	.06 [-.01, .12]

Item 30	.07 [-.01, .15]	.04 [-.05, .13]	.16* [.10, .23]	.18* [.12, .25]	.02 [-.05, .10]	.12* [.06, .18]	-.01 [-.07, .05]	-.11* [-.16, -.05]	.09* [.02, .16]	-.09* [-.15, -.02]
Item 31	-.07 [-.15, .00]	-.05 [-.14, .04]	-.06* [-.12, .00]	-.05 [-.11, .01]	-.09* [-.16, -.02]	-.04 [-.10, .02]	-.06 [-.12, .01]	.06* [.00, .12]	-.07 [-.13, .00]	.08* [.02, .14]
Item 32	.07 [-.02, .15]	.04 [-.05, .13]	.03 [-.03, .09]	.00 [-.06, .06]	.05 [-.02, .12]	.08* [.02, .13]	.06* [.00, .12]	-.07* [-.12, -.02]	.10* [.03, .16]	-.05 [-.10, .01]
Item 33	.02 [-.06, .11]	-.01 [-.11, .09]	.03 [-.04, .10]	.01 [-.07, .08]	.11* [.03, .18]	.04 [-.03, .11]	.02 [-.05, .09]	-.10* [-.16, -.03]	.09* [.01, .17]	-.07 [-.13, .00]
Item 34	-.11* [-.18, -.03]	-.06 [-.15, .03]	-.06* [-.12, .00]	-.03 [-.10, .04]	-.08* [-.15, -.02]	-.07* [-.12, -.01]	-.05 [-.10, .01]	.09* [.02, .15]	-.10* [-.16, -.03]	.10* [.04, .15]
Item 35	.14* [.06, .21]	.01 [-.08, .09]	.07* [.01, .12]	.08* [.02, .14]	.08* [.02, .15]	.02 [-.04, .07]	.02 [-.04, .08]	-.08* [-.13, -.03]	.07 [.00, .14]	-.05 [-.10, .01]
Item 36	.02 [-.06, .10]	.00 [-.09, .09]	.06 [-.01, .12]	.05 [-.01, .11]	.00 [-.07, .08]	-.03 [-.08, .04]	.01 [-.05, .07]	-.01 [-.07, .05]	.00 [-.07, .07]	-.02 [-.08, .04]
Item 37	.01 [-.07, .09]	.05 [-.05, .14]	-.05 [-.11, .02]	-.07* [-.13, .00]	.01 [-.06, .09]	-.06 [-.12, .01]	.06 [.00, .13]	.02 [-.04, .09]	.00 [-.07, .08]	.05 [-.02, .12]
Item 38	-.02 [-.09, .06]	-.08 [-.17, .01]	.00 [-.06, .06]	-.06 [-.11, .01]	.01 [-.06, .08]	-.02 [-.07, .04]	-.05 [-.12, .02]	.05 [-.01, .10]	-.05 [-.12, .02]	.05 [-.01, .11]
Item 39	.03 [-.06, .12]	.17* [.07, .26]	.02 [-.05, .09]	.05 [-.02, .12]	.08 [-.01, .16]	.02 [-.05, .09]	.19* [.12, .25]	.02 [-.05, .09]	.03 [-.05, .11]	-.03 [-.10, .04]
Item 40	-.04 [-.10, .04]	-.02 [-.11, .06]	-.03 [-.09, .03]	-.04 [-.10, .02]	-.05 [-.11, .01]	-.10* [-.15, -.05]	.00 [-.05, .06]	.17* [.12, .22]	-.06* [-.12, .00]	.07* [.02, .13]
Item 41	.03 [-.05, .12]	-.01 [-.11, .08]	.07 [.00, .14]	.10* [.03, .17]	.00 [-.08, .08]	.10* [.03, .16]	-.02 [-.09, .05]	-.05 [-.11, .02]	.03 [-.05, .11]	-.06 [-.13, .01]
Item 42	.01 [-.05, .08]	-.01 [-.09, .07]	-.06* [-.11, -.01]	-.05 [-.10, .00]	-.02 [-.07, .04]	-.09* [-.13, -.04]	.09* [.04, .14]	.18* [.14, .22]	-.04 [-.09, .02]	.04 [-.01, .09]
Item 43	-.04 [-.12, .04]	-.05 [-.14, .05]	-.06 [-.13, .00]	-.08* [-.15, -.02]	.00 [-.07, .08]	-.01 [-.08, .05]	-.04 [-.10, .03]	.05 [-.01, .11]	-.07 [-.14, .01]	.13* [.06, .19]
Item 44	-.06 [-.14, .02]	-.06 [-.15, .03]	.00 [-.06, .06]	-.03 [-.10, .03]	-.02 [-.10, .05]	-.05 [-.11, .01]	-.07* [-.13, .00]	.12* [.06, .18]	-.07 [-.13, .01]	.04 [-.03, .10]
Item 45	.00 [-.09, .08]	.01 [-.09, .11]	.01 [-.06, .08]	.02 [-.05, .09]	.03 [-.04, .11]	.03 [-.03, .10]	.04 [-.03, .10]	-.07* [-.13, -.01]	.07 [.00, .15]	-.07 [-.14, .00]
Item 46	-.06 [-.13, .01]	-.05 [-.13, .04]	-.02 [-.08, .03]	-.06* [-.11, -.01]	-.05 [-.11, .02]	-.09* [-.13, -.04]	-.05 [-.10, .00]	.07* [.02, .12]	-.06* [-.12, .00]	.07* [.02, .12]
Item 47	.09* [.01, .16]	.10* [.01, .19]	.07* [.00, .13]	.04 [-.03, .10]	.04 [-.03, .11]	.06* [.01, .12]	.13* [.07, .18]	-.06* [-.12, -.01]	.13* [.06, .20]	-.05 [-.11, .01]
Item 48	.03 [-.04, .11]	.03 [-.06, .11]	.06* [.01, .12]	.08* [.02, .13]	.02 [-.05, .08]	.26* [.21, .31]	-.02 [-.08, .03]	-.05* [-.10, .00]	.03 [-.04, .09]	-.06* [-.12, .00]
Item 49	-.06 [-.13, .02]	-.04 [-.13, .05]	-.08* [-.15, -.02]	-.07* [-.14, -.01]	-.08* [-.15, .00]	-.08* [-.15, .00]	-.06* [-.13, .00]	.09* [.03, .16]	-.10* [-.17, -.03]	.12* [.04, .20]
Item 50	.04 [-.04, .11]	.08 [-.02, .17]	.02 [-.04, .08]	.02 [-.04, .08]	.02 [-.05, .09]	.02 [-.04, .07]	.11* [.06, .17]	-.02 [-.08, .03]	.04 [-.02, .11]	-.03 [-.09, .03]
	Item 21	Item 22	Item 23	Item 24	Item 25	Item 26	Item 27	Item 28	Item 29	Item 30
Item 22	.04 [-.04, .12]									
Item 23	.05 [-.03, .14]	.00 [-.07, .06]								
Item 24	.09* [.01, .17]	.04 [-.05, .12]	.03 [-.06, .12]							
Item 25	-.03 [-.12, .06]	-.03 [-.10, .04]	-.06 [-.14, .03]	-.02 [-.11, .08]						
Item 26	-.04 [-.12, .05]	-.03 [-.10, .03]	-.03 [-.11, .05]	-.04 [-.13, .04]	.01 [-.08, .10]					
Item 27	-.05 [-.12, .03]	-.03 [-.08, .02]	.05 [-.02, .12]	.00 [-.08, .07]	.07* [.00, .15]	.04 [-.03, .11]				
Item 28	-.06 [-.14, .02]	-.05 [-.11, .00]	-.06 [-.14, .02]	-.06 [-.14, .02]	.05 [-.03, .12]	.04 [-.03, .11]	.13* [.07, .19]			
Item 29	-.07 [-.14, .01]	-.01 [-.06, .05]	-.02 [-.09, .06]	-.06 [-.14, .02]	.03 [-.05, .11]	.03 [-.04, .10]	.08* [.02, .14]	.09* [.02, .15]		
Item 30	.05 [-.03, .13]	.08* [.02, .14]	.10* [.02, .17]	.05 [-.03, .14]	-.04 [-.12, .04]	-.07 [-.14, .01]	-.05 [-.11, .02]	-.07* [-.13, .00]	-.06 [-.13, .01]	
Item 31	-.02 [-.10, .06]	.00 [-.06, .06]	-.06 [-.13, .02]	-.04 [-.12, .04]	.07 [-.01, .15]	.04 [-.04, .11]	.16* [.10, .23]	.26* [.20, .32]	.07 [.00, .14]	-.07 [-.13, .00]
Item 32	.04 [-.04, .12]	.06* [.00, .11]	.06 [-.01, .14]	.03 [-.05, .10]	-.03 [-.10, .04]	-.07 [-.13, .00]	.01 [-.05, .06]	-.05 [-.11, .01]	-.05 [-.11, .01]	.04 [-.03, .11]
Item 33	.06 [-.03, .14]	.00 [-.07, .06]	.20* [.12, .28]	.03 [-.06, .12]	-.01 [-.09, .08]	-.08 [-.16, .00]	.03 [-.05, .10]	.00 [-.07, .07]	-.05 [-.12, .02]	.07 [-.01, .15]
Item 34	-.06 [-.13, .02]	.04 [-.02, .09]	-.05 [-.12, .03]	-.03 [-.10, .05]	.06 [-.02, .13]	.05 [-.02, .12]	.10* [.04, .16]	.10* [.04, .16]	.12* [.05, .18]	-.06* [-.13, .00]
Item 35	.04 [-.03, .12]	.06* [.00, .11]	.04 [-.03, .11]	.06 [-.02, .13]	-.03 [-.10, .05]	-.05 [-.12, .02]	-.08* [-.14, -.02]	-.08* [-.13, -.02]	-.05 [-.11, .01]	.09* [.03, .16]
Item 36	-.01 [-.10, .07]	.09* [.03, .15]	-.02 [-.10, .05]	.04 [-.05, .12]	-.01 [-.08, .07]	.01 [-.06, .08]	-.04 [-.10, .03]	-.12* [-.18, -.06]	-.09* [-.15, -.02]	.03 [-.04, .10]

Item 37	-.04 [-.12, .04]	-.04 [-.10, .03]	-.04 [-.12, .04]	-.06 [-.15, .02]	.08 [-.01, .16]	.11* [.04, .19]	-.03 [-.09, .04]	.03 [-.04, .09]	.04 [-.03, .11]	-.05 [-.12, .02]
Item 38	-.01 [-.08, .07]	-.01 [-.07, .04]	.00 [-.07, .07]	-.01 [-.09, .07]	.20* [.13, .26]	.01 [-.07, .08]	.06 [.00, .12]	.05 [-.01, .11]	.03 [-.03, .10]	-.02 [-.09, .05]
Item 39	.01 [-.08, .10]	.00 [-.07, .07]	-.01 [-.09, .07]	.01 [-.08, .10]	-.08 [-.16, .01]	-.03 [-.11, .05]	-.06 [-.13, .01]	.01 [-.06, .09]	.00 [-.08, .07]	.01 [-.07, .09]
Item 40	-.08* [-.15, -.01]	.00 [-.05, .05]	-.08* [-.14, -.01]	-.05 [-.12, .03]	.03 [-.05, .10]	.08* [.02, .15]	.00 [-.06, .06]	.03 [-.03, .09]	.14* [.08, .20]	-.09* [-.16, -.03]
Item 41	.06 [-.03, .14]	.03 [-.04, .10]	.05 [-.03, .13]	.13* [.03, .22]	-.06 [-.15, .02]	-.01 [-.09, .07]	-.02 [-.09, .05]	-.03 [-.10, .04]	-.04 [-.11, .03]	.08* [.01, .16]
Item 42	-.02 [-.09, .05]	-.02 [-.07, .02]	-.07* [-.13, .00]	-.04 [-.11, .04]	.02 [-.06, .11]	.03 [-.03, .09]	.01 [-.04, .06]	.03 [-.02, .08]	.13* [.07, .18]	-.08* [-.13, -.02]
Item 43	-.04 [-.13, .04]	-.04 [-.11, .02]	-.03 [-.10, .06]	-.04 [-.13, .04]	.06 [-.02, .15]	.09* [.01, .18]	.07 [.00, .13]	.07* [.00, .14]	.05 [-.03, .12]	-.12* [-.19, -.05]
Item 44	-.04 [-.13, .04]	.01 [-.06, .07]	-.03 [-.10, .05]	-.04 [-.12, .04]	.04 [-.05, .12]	.07 [-.01, .14]	.05 [-.02, .12]	.04 [-.03, .11]	.09* [.02, .16]	-.02 [-.10, .06]
Item 45	.06 [-.03, .14]	.00 [-.07, .06]	.29* [.20, .37]	.05 [-.03, .14]	-.04 [-.12, .04]	-.04 [-.12, .04]	.02 [-.05, .08]	-.07 [-.15, .01]	-.02 [-.09, .05]	.05 [-.03, .12]
Item 46	-.07 [-.14, .01]	-.02 [-.07, .03]	-.08* [-.15, .00]	-.06 [-.13, .02]	.12* [.05, .19]	.06 [-.01, .13]	.19* [.14, .24]	.12* [.06, .18]	.06 [.00, .12]	-.06 [-.11, .00]
Item 47	.04 [-.04, .12]	.04 [-.01, .10]	-.01 [-.08, .07]	.03 [-.05, .10]	-.08* [-.15, -.01]	-.04 [-.11, .03]	-.06 [-.12, .01]	-.02 [-.08, .04]	-.06 [-.12, .01]	.06 [.00, .13]
Item 48	.05 [-.02, .13]	-.04 [-.10, .01]	.02 [-.05, .09]	.06 [-.01, .14]	-.05 [-.12, .02]	-.02 [-.09, .05]	-.03 [-.09, .02]	-.02 [-.07, .04]	-.03 [-.08, .03]	.04 [-.03, .10]
Item 49	-.03 [-.11, .05]	-.02 [-.08, .04]	-.07 [-.14, .02]	-.08 [-.16, .01]	.08 [.00, .16]	.13* [.05, .20]	.07* [.00, .14]	.02 [-.05, .09]	.09* [.02, .16]	-.10* [-.17, -.03]
Item 50	.08* [.00, .15]	.06* [.01, .12]	-.01 [-.08, .06]	.06 [-.02, .14]	-.05 [-.12, .02]	.00 [-.06, .07]	-.02 [-.08, .04]	-.02 [-.08, .04]	-.04 [-.10, .02]	.01 [-.06, .08]
	Item 31	Item 32	Item 33	Item 34	Item 35	Item 36	Item 37	Item 38	Item 39	Item 40
Item 32	-.01 [-.07, .06]									
Item 33	.00 [-.07, .07]	.22* [.15, .28]								
Item 34	.14* [.07, .20]	-.10* [-.15, -.04]	-.07* [-.14, -.01]							
Item 35	-.08* [-.14, -.02]	.11* [.05, .17]	.10* [.02, .18]	-.10* [-.15, -.04]						
Item 36	-.11* [-.18, -.05]	-.01 [-.08, .06]	.02 [-.06, .10]	-.01 [-.07, .05]	.15* [.08, .21]					
Item 37	.02 [-.05, .09]	.00 [-.06, .07]	-.01 [-.08, .07]	.00 [-.07, .07]	-.03 [-.09, .04]	.00 [-.07, .07]				
Item 38	.08* [.01, .14]	-.05 [-.11, .01]	-.01 [-.08, .06]	.03 [-.04, .09]	-.02 [-.08, .04]	-.02 [-.09, .04]	-.02 [-.09, .05]			
Item 39	-.05 [-.12, .03]	.03 [-.05, .10]	.01 [-.07, .09]	-.07 [-.14, .00]	.01 [-.07, .08]	-.02 [-.09, .06]	-.01 [-.09, .08]	-.09* [-.16, -.01]		
Item 40	.00 [-.06, .06]	-.07* [-.12, -.01]	-.11* [-.17, -.05]	.13* [.07, .18]	-.06* [-.11, -.01]	.01 [-.06, .07]	.01 [-.05, .08]	.03 [-.03, .09]	.00 [-.07, .07]	
Item 41	-.05 [-.12, .03]	-.01 [-.08, .07]	.01 [-.07, .10]	-.02 [-.09, .05]	.03 [-.05, .10]	.01 [-.07, .08]	-.09* [-.17, -.01]	.01 [-.07, .08]	.00 [-.09, .08]	-.06 [-.13, .01]
Item 42	.09* [.04, .15]	-.04 [-.09, .01]	-.07* [-.13, -.01]	.07* [.02, .12]	-.07* [-.12, -.03]	-.10* [-.15, -.04]	.06* [.00, .12]	.03 [-.03, .09]	.06 [.00, .13]	.10* [.05, .15]
Item 43	.08* [.01, .15]	-.03 [-.10, .04]	-.04 [-.11, .04]	.07* [.00, .14]	-.05 [-.11, .02]	-.05 [-.12, .02]	.06 [-.02, .14]	.06 [-.01, .13]	-.06 [-.13, .03]	.04 [-.03, .11]
Item 44	.04 [-.04, .11]	-.07* [-.13, -.01]	-.05 [-.12, .03]	.13* [.06, .19]	-.05 [-.12, .01]	-.01 [-.08, .06]	.03 [-.05, .10]	.04 [-.03, .11]	-.04 [-.12, .04]	.15* [.07, .22]
Item 45	-.09* [-.16, -.02]	.08* [.01, .15]	.21* [.13, .29]	-.05 [-.11, .03]	.02 [-.05, .10]	-.01 [-.08, .07]	-.02 [-.10, .05]	-.07 [-.14, .00]	-.01 [-.09, .07]	-.07* [-.14, -.01]
Item 46	.16* [.10, .23]	-.08* [-.13, -.03]	-.06 [-.13, .00]	.13* [.07, .19]	-.06* [-.11, -.01]	-.02 [-.08, .04]	.03 [-.03, .10]	.05 [-.01, .11]	-.09* [-.16, -.02]	.09* [.03, .14]
Item 47	-.08* [-.14, -.02]	.11* [.05, .17]	.04 [-.04, .12]	-.08* [-.13, -.02]	.07* [.00, .13]	.05 [-.02, .11]	.02 [-.04, .09]	-.07* [-.13, -.01]	.11* [.04, .19]	-.02 [-.07, .04]
Item 48	-.03 [-.09, .03]	.04 [-.02, .10]	.00 [-.07, .07]	-.02 [-.08, .04]	-.03 [-.08, .03]	-.02 [-.08, .04]	-.06 [-.13, .00]	-.03 [-.09, .03]	.05 [-.02, .12]	-.07* [-.13, -.02]
Item 49	.07 [-.01, .14]	-.10* [-.16, -.03]	-.10* [-.17, -.02]	.11* [.04, .18]	-.07* [-.13, .00]	-.02 [-.09, .05]	.06 [-.02, .14]	.07 [.00, .14]	-.06 [-.14, .02]	.10* [.04, .17]
Item 50	-.03 [-.09, .04]	.02 [-.04, .08]	-.02 [-.09, .05]	-.04 [-.10, .02]	.03 [-.03, .08]	.03 [-.04, .09]	.06 [-.01, .13]	-.09* [-.15, -.03]	.04 [-.04, .12]	-.05 [-.10, .01]
	Item 41	Item 42	Item 43	Item 44	Item 45	Item 46	Item 47	Item 48	Item 49	Item 50
Item 42	-.04 [-.10, .02]									
Item 43	-.04 [-.12, .04]	.03 [-.03, .09]								

Item 44	-.03 [-.10, .05]	.04 [-.02, .10]	.06 [-.01, .14]							
Item 45	.04 [-.04, .12]	-.04 [-.10, .02]	-.04 [-.11, .04]	-.03 [-.10, .05]						
Item 46	-.06 [-.12, .01]	.02 [-.03, .07]	.08* [.01, .14]	.06 [.00, .13]	-.07 [-.14, .00]					
Item 47	.01 [-.07, .08]	-.02 [-.07, .03]	-.06 [-.12, .01]	-.06 [-.12, .01]	.02 [-.05, .09]	-.08* [-.13, -.02]				
Item 48	.10* [.04, .17]	-.05* [-.10, .00]	.00 [-.07, .06]	-.08* [-.15, -.02]	.01 [-.06, .08]	-.09* [-.14, -.04]	.03 [-.03, .09]			
Item 49	-.02 [-.10, .06]	.04 [-.02, .10]	.15* [.07, .22]	.10* [.02, .17]	-.06 [-.14, .02]	.10* [.04, .17]	-.07* [-.14, .00]	-.03 [-.10, .04]		
Item 50	.01 [-.06, .08]	-.03 [-.08, .02]	-.02 [-.08, .05]	-.05 [-.11, .02]	.01 [-.06, .08]	-.07* [-.12, -.01]	.06* [.00, .12]	.04 [-.01, .10]	-.05 [-.11, .02]	

Sample B: Dutch-speaking Belgian

First-order factor loadings	Identification-self	Comprehension-self	Expression-self	Regulation-self	Utilization-self	Identification-other	Comprehension-other	Expression-other	Regulation-other	Utilization-other
Item 6	**.37* [.14, .61]**	.02 [-.11, .14]	-.01 [-.11, .08]	.01 [-.10, .12]	.05 [-.04, .13]	.00 [-.10, .10]	.01 [-.10, .11]	.01 [-.10, .11]	.00 [-.12, .12]	.01 [-.07, .10]
Item 16	**.44* [.21, .66]**	.00 [-.12, .13]	.00 [-.10, .09]	-.03 [-.14, .08]	.02 [-.06, .10]	.00 [-.11, .10]	.00 [-.10, .10]	.00 [-.11, .10]	-.01 [-.13, .11]	-.01 [-.09, .08]
Item 20	**.50* [.27, .73]**	.03 [-.09, .16]	.00 [-.10, .10]	-.01 [-.12, .10]	-.04 [-.12, .04]	-.01 [-.11, .10]	.00 [-.11, .10]	-.01 [-.12, .10]	.00 [-.13, .12]	-.01 [-.09, .07]
Item 48	**.44* [.26, .60]**	.02 [-.10, .14]	.00 [-.09, .10]	-.01 [-.12, .09]	.04 [-.04, .12]	.01 [-.09, .11]	.01 [-.09, .12]	.01 [-.10, .12]	-.01 [-.13, .11]	-.01 [-.09, .08]
Item 49	**.62* [.39, .83]**	.07 [-.05, .19]	.00 [-.09, .10]	-.03 [-.14, .08]	.00 [-.08, .08]	.01 [-.10, .11]	.01 [-.10, .11]	-.01 [-.12, .10]	.00 [-.13, .12]	-.02 [-.10, .06]
Item 1	-.01 [-.10, .07]	**.72* [.59, .86]**	-.01 [-.10, .08]	.02 [-.09, .12]	-.02 [-.10, .05]	-.01 [-.11, .09]	-.01 [-.12, .09]	-.02 [-.12, .09]	.00 [-.12, .12]	-.01 [-.09, .07]
Item 2	.01 [-.07, .10]	**.50* [.34, .65]**	.03 [-.06, .13]	.05 [-.05, .16]	-.02 [-.10, .06]	.03 [-.07, .13]	.02 [-.08, .12]	.01 [-.09, .12]	.03 [-.09, .15]	.00 [-.08, .08]
Item 10	.01 [-.07, .10]	**.13 [-.04, .29]**	.00 [-.09, .09]	-.07 [-.18, .04]	.09* [.01, .17]	.02 [-.08, .12]	.02 [-.08, .13]	.02 [-.09, .13]	-.01 [-.13, .11]	.01 [-.08, .10]
Item 26	.00 [-.09, .08]	**.62* [.47, .77]**	-.01 [-.11, .08]	.04 [-.06, .15]	-.02 [-.10, .06]	-.01 [-.11, .09]	-.01 [-.11, .09]	-.02 [-.13, .08]	.00 [-.12, .13]	.00 [-.09, .08]
Item 43	.02 [-.07, .10]	**.66* [.50, .81]**	.00 [-.09, .09]	.03 [-.08, .14]	-.02 [-.10, .06]	-.02 [-.12, .09]	-.02 [-.12, .08]	-.01 [-.12, .09]	-.01 [-.13, .11]	-.03 [-.11, .05]
Item 8	.00 [-.09, .08]	-.01 [-.13, .10]	**.65* [.51, .81]**	.03 [-.08, .13]	.02 [-.06, .10]	.00 [-.10, .10]	.00 [-.10, .10]	-.02 [-.12, .08]	.03 [-.09, .15]	.06 [-.03, .14]
Item 17	.01 [-.07, .10]	.04 [-.07, .15]	**.18* [.04, .34]**	.21* [.10, .32]	-.02 [-.10, .06]	-.01 [-.11, .09]	.00 [-.10, .10]	.01 [-.10, .11]	.09 [-.03, .21]	.02 [-.06, .10]
Item 25	.00 [-.09, .08]	.01 [-.10, .12]	**.74* [.58, .89]**	-.03 [-.13, .07]	.02 [-.06, .10]	-.01 [-.11, .10]	.00 [-.10, .10]	.00 [-.10, .11]	.00 [-.12, .12]	.04 [-.04, .12]
Item 38	.00 [-.08, .09]	-.01 [-.12, .10]	**.55* [.44, .70]**	-.03 [-.14, .08]	.02 [-.06, .10]	.01 [-.09, .12]	.00 [-.10, .10]	.01 [-.09, .12]	.00 [-.12, .12]	.00 [-.09, .09]
Item 42	.01 [-.07, .10]	.03 [-.08, .15]	**.26* [.10, .42]**	.07 [-.04, .17]	.00 [-.08, .08]	.03 [-.07, .13]	.04 [-.06, .14]	.04 [-.06, .15]	.05 [-.07, .17]	.03 [-.06, .11]
Item 12	-.02 [-.11, .06]	-.04 [-.16, .07]	-.03 [-.12, .06]	**.73* [.62, .86]**	-.02 [-.10, .06]	-.02 [-.12, .09]	-.01 [-.12, .09]	-.02 [-.13, .08]	-.04 [-.16, .08]	.03 [-.05, .11]
Item 15	.00 [-.09, .08]	-.01 [-.12, .11]	.03 [-.06, .13]	**.63* [.49, .78]**	-.01 [-.09, .07]	-.01 [-.12, .09]	-.01 [-.12, .09]	-.03 [-.13, .08]	-.03 [-.16, .09]	.03 [-.06, .11]
Item 37	.03 [-.05, .12]	.17* [.06, .29]	.03 [-.06, .12]	**.39* [.24, .53]**	-.03 [-.10, .05]	.01 [-.09, .11]	.02 [-.08, .12]	.02 [-.08, .12]	-.01 [-.13, .12]	.00 [-.08, .08]
Item 39	-.01 [-.09, .08]	.00 [-.11, .11]	-.01 [-.10, .08]	**.62* [.47, .76]**	-.02 [-.10, .06]	.00 [-.11, .10]	.00 [-.10, .10]	.00 [-.11, .10]	-.01 [-.13, .12]	-.04 [-.12, .05]
Item 50	.01 [-.08, .09]	.02 [-.09, .13]	.01 [-.09, .10]	**.41* [.25, .55]**	.05 [-.03, .14]	.01 [-.09, .12]	.01 [-.09, .12]	-.01 [-.11, .10]	.02 [-.10, .14]	.00 [-.08, .09]
Item 9	-.02 [-.11, .06]	-.05 [-.16, .06]	-.01 [-.10, .08]	-.23* [-.34, -.12]	**.20* [.09, .31]**	.03 [-.07, .13]	.03 [-.07, .13]	.04 [-.06, .15]	-.04 [-.16, .08]	-.01 [-.10, .07]
Item 21	-.01 [-.09, .07]	-.03 [-.15, .08]	-.01 [-.10, .09]	.01 [-.09, .12]	**.55* [.49, .63]**	-.01 [-.11, .10]	.00 [-.11, .10]	-.01 [-.11, .10]	.02 [-.11, .14]	.05 [-.04, .13]
Item 22	.03 [-.05, .12]	.06 [-.04, .17]	.04 [-.05, .13]	.12* [.01, .22]	**.21* [.10, .32]**	.03 [-.06, .14]	.04 [-.06, .14]	.06 [-.04, .17]	.07 [-.05, .19]	-.02 [-.10, .06]
Item 24	.00 [-.09, .08]	-.01 [-.12, .11]	.00 [-.09, .10]	.00 [-.11, .11]	**.56* [.45, .67]**	.00 [-.10, .10]	.00 [-.11, .10]	-.01 [-.11, .10]	.01 [-.12, .13]	-.01 [-.10, .08]
Item 41	.02 [-.06, .10]	.02 [-.09, .14]	.02 [-.07, .12]	-.02 [-.13, .09]	**.51* [.39, .61]**	.02 [-.08, .13]	.02 [-.08, .13]	.03 [-.08, .14]	-.01 [-.13, .12]	-.02 [-.11, .07]
Item 7	.00 [-.09, .08]	-.01 [-.12, .09]	-.03 [-.12, .06]	.00 [-.10, .10]	.00 [-.08, .08]	**.56* [.35, .76]**	.00 [-.11, .10]	-.01 [-.13, .10]	.00 [-.13, .13]	-.01 [-.09, .08]
Item 29	.01 [-.07, .09]	.02 [-.08, .14]	.02 [-.06, .12]	.01 [-.09, .11]	-.01 [-.08, .07]	**.57* [.42, .74]**	.01 [-.09, .12]	.02 [-.09, .13]	.01 [-.11, .14]	-.01 [-.08, .07]

Item 30	-.01 [-.10, .07]	-.02 [-.12, .09]	-.02 [-.11, .06]	-.04 [-.13, .06]	.02 [-.05, .09]	**.62* [.43, .81]**	.03 [-.08, .14]	.02 [-.09, .13]	.02 [-.10, .14]	.00 [-.08, .07]
Item 40	.01 [-.07, .09]	.02 [-.08, .13]	-.01 [-.10, .08]	-.01 [-.11, .09]	-.01 [-.08, .07]	**.56* [.36, .76]**	.01 [-.10, .11]	-.01 [-.13, .10]	-.03 [-.16, .10]	.00 [-.08, .08]
Item 44	.00 [-.09, .08]	.01 [-.09, .12]	.00 [-.09, .09]	-.02 [-.12, .08]	.02 [-.06, .09]	**.68* [.47, .87]**	.01 [-.10, .12]	-.01 [-.12, .11]	-.04 [-.16, .09]	-.01 [-.09, .07]
Item 5	.01 [-.08, .09]	.00 [-.11, .12]	.01 [-.09, .10]	-.03 [-.13, .08]	.02 [-.06, .10]	.01 [-.10, .12]	**.57* [.35, .79]**	-.01 [-.12, .10]	-.02 [-.15, .11]	.00 [-.08, .08]
Item 13	.00 [-.09, .08]	-.01 [-.12, .09]	.00 [-.10, .08]	.00 [-.10, .10]	.02 [-.06, .09]	.03 [-.08, .13]	**.56* [.40, .74]**	-.01 [-.13, .09]	.00 [-.12, .13]	.03 [-.05, .11]
Item 14	.00 [-.08, .08]	-.04 [-.15, .07]	-.01 [-.10, .08]	-.02 [-.12, .08]	.02 [-.06, .09]	.02 [-.09, .13]	**.56* [.33, .78]**	.03 [-.09, .14]	-.01 [-.14, .12]	-.03 [-.11, .05]
Item 18	.01 [-.08, .09]	.03 [-.08, .14]	.02 [-.07, .11]	.01 [-.09, .11]	.01 [-.07, .09]	.01 [-.10, .11]	**.45* [.22, .67]**	-.02 [-.13, .09]	-.02 [-.15, .11]	.05 [-.04, .13]
Item 34	.01 [-.08, .09]	.01 [-.10, .12]	.00 [-.09, .10]	-.02 [-.12, .09]	.01 [-.07, .09]	.02 [-.09, .13]	**.54* [.32, .76]**	.02 [-.10, .13]	-.02 [-.15, .11]	-.02 [-.11, .06]
Item 23	-.01 [-.10, .07]	-.06 [-.17, .05]	-.01 [-.10, .08]	-.01 [-.12, .09]	.02 [-.06, .09]	.02 [-.08, .13]	.00 [-.10, .10]	**.62* [.46, .79]**	.05 [-.07, .18]	-.03 [-.11, .06]
Item 28	-.01 [-.09, .08]	.00 [-.11, .11]	-.02 [-.11, .08]	.01 [-.10, .11]	-.02 [-.10, .06]	-.01 [-.12, .09]	-.01 [-.12, .10]	**.49* [.30, .67]**	.01 [-.12, .14]	-.07 [-.16, .01]
Item 31	.00 [-.08, .08]	.00 [-.11, .11]	.00 [-.09, .09]	-.01 [-.11, .09]	-.01 [-.09, .07]	-.02 [-.12, .08]	-.01 [-.12, .09]	**.64* [.50, .80]**	.00 [-.13, .12]	-.06 [-.14, .02]
Item 45	-.01 [-.09, .08]	-.02 [-.13, .09]	-.02 [-.11, .07]	.02 [-.09, .12]	.00 [-.08, .08]	.00 [-.11, .10]	.00 [-.11, .10]	**.53* [.35, .70]**	.03 [-.09, .16]	.00 [-.08, .08]
Item 46	.01 [-.07, .09]	.02 [-.08, .13]	.01 [-.08, .10]	.02 [-.08, .12]	.00 [-.08, .08]	.02 [-.09, .13]	.02 [-.08, .13]	**.42* [.25, .61]**	.06 [-.07, .19]	.00 [-.08, .08]
Item 19	-.01 [-.09, .08]	-.02 [-.13, .09]	-.01 [-.10, .09]	-.04 [-.15, .06]	-.01 [-.08, .07]	-.02 [-.12, .09]	-.02 [-.12, .09]	.00 [-.11, .11]	**.75* [.60, .91]**	.02 [-.06, .10]
Item 27	.02 [-.06, .11]	.06 [-.05, .17]	.04 [-.04, .14]	-.05 [-.15, .06]	.02 [-.06, .10]	.06 [-.04, .18]	.05 [-.05, .17]	.14* [.03, .26]	**.30* [.10, .49]**	.01 [-.07, .09]
Item 33	.01 [-.08, .09]	.00 [-.11, .11]	.03 [-.06, .12]	-.01 [-.11, .10]	.01 [-.07, .09]	.01 [-.09, .12]	.01 [-.10, .11]	.08 [-.03, .19]	**.50* [.31, .69]**	.04 [-.04, .13]
Item 35	.00 [-.09, .08]	-.02 [-.13, .09]	.00 [-.09, .09]	-.02 [-.12, .10]	.04 [-.04, .12]	.01 [-.09, .11]	.01 [-.10, .11]	.00 [-.11, .10]	**.25* [.05, .45]**	.19* [.10, .28]
Item 47	.00 [-.08, .08]	-.01 [-.12, .10]	-.01 [-.10, .09]	.05 [-.06, .16]	-.02 [-.09, .06]	-.02 [-.13, .08]	-.02 [-.13, .08]	-.02 [-.13, .09]	**.58* [.38, .77]**	.06 [-.02, .15]
Item 3	-.01 [-.09, .08]	-.02 [-.13, .08]	.00 [-.09, .09]	.02 [-.08, .13]	.02 [-.06, .09]	-.01 [-.11, .10]	-.01 [-.11, .09]	-.02 [-.13, .09]	.02 [-.11, .14]	**.62* [.54, .71]**
Item 4	.01 [-.07, .10]	.03 [-.08, .14]	.03 [-.07, .12]	.05 [-.05, .16]	-.01 [-.09, .07]	.00 [-.11, .10]	-.01 [-.11, .10]	-.03 [-.14, .08]	.03 [-.10, .15]	**.59* [.47, .70]**
Item 11	.00 [-.08, .09]	.00 [-.11, .11]	.02 [-.07, .11]	.05 [-.05, .16]	-.02 [-.09, .07]	.00 [-.10, .10]	-.01 [-.11, .10]	-.01 [-.12, .09]	.02 [-.10, .15]	**.62* [.51, .74]**
Item 32	.02 [-.06, .10]	.03 [-.07, .13]	.03 [-.05, .12]	.06 [-.04, .16]	.00 [-.08, .07]	.06 [-.04, .17]	.06 [-.04, .17]	.08 [-.02, .18]	.19* [.07, .32]	**.22* [.12, .32]**
Item 36	-.01 [-.10, .07]	-.03 [-.14, .08]	-.02 [-.11, .07]	-.04 [-.14, .06]	.01 [-.08, .08]	-.02 [-.12, .08]	-.01 [-.11, .09]	-.04 [-.15, .06]	-.01 [-.13, .11]	**.41* [.30, .53]**

Second-order factor loadings	Intrapersonal EC	Interpersonal EC
Identification-self	**.93* [.71, 1.20]**	.02 [-.31, .30]
Comprehension-self	**.88* [.72, 1.07]**	-.03 [-.27, .18]
Expression-self	**.70* [.46, .94]**	.11 [-.18, .36]
Regulation-self	**.74* [.51, .94]**	-.07 [-.29, .17]
Utilization-self	**.14 [-.23, .49]**	.23 [-.06, .48]
Identification-other	-.03 [-.31, .19]	**.96* [.80, 1.19]**
Comprehension-other	.02 [-.25, .24]	**.93* [.74, 1.14]**
Expression-other	-.07 [-.32, .16]	**.90* [.71, 1.09]**
Regulation-other	.06 [-.17, .26]	**.80* [.60, .98]**
Utilization-other	.10 [-.16, .33]	**.40* [.08, .67]**

Factor correlation	Interpersonal EC
Intrapersonal EC	**.80* [.60, .93]**

Residual correlation	Identification-self	Comprehension-self	Expression-self	Regulation-self	Utilization-self	Identification-other	Comprehension-other	Expression-other	Regulation-other	Utilization-other
Comprehension-self	.04 [-.10, .17]									
Expression-self	-.01 [-.14, .13]	.01 [-.12, .13]								
Regulation-self	-.01 [-.15, .12]	.05 [-.08, .19]	.03 [-.10, .16]							
Utilization-self	.01 [-.12, .14]	-.04 [-.16, .09]	.03 [-.09, .14]	-.04 [-.17, .08]						
Identification-other	**.00 [-.14, .14]**	.01 [-.12, .14]	-.01 [-.14, .12]	-.02 [-.15, .12]	.01 [-.11, .14]					
Comprehension-other	.01 [-.13, .15]	**.18* [.05, .31]**	.00 [-.13, .13]	-.01 [-.15, .12]	.02 [-.11, .14]	.05 [-.09, .18]				
Expression-other	.00 [-.14, .13]	.00 [-.12, .13]	**.07 [-.06, .19]**	.01 [-.12, .14]	.00 [-.12, .13]	.01 [-.12, .14]	.00 [-.14, .13]			
Regulation-other	-.01 [-.15, .13]	.02 [-.12, .15]	.04 [-.09, .17]	**.51* [.40, .60]**	-.02 [-.15, .11]	-.02 [-.15, .12]	-.03 [-.16, .11]	.07 [-.07, .20]		
Utilization-other	-.01 [-.15, .13]	-.02 [-.15, .11]	.06 [-.06, .18]	.07 [-.05, .20]	**.11 [-.01, .22]**	-.01 [-.15, .12]	-.01 [-.14, .12]	-.06 [-.18, .06]	.10 [-.03, .23]	

	Item 1	Item 2	Item 3	Item 4	Item 5	Item 6	Item 7	Item 8	Item 9	Item 10
Item 2	.09* [.01, .17]									
Item 3	-.05 [-.13, .03]	-.03 [-.11, .04]								
Item 4	-.06 [-.13, .03]	-.01 [-.08, .06]	.06 [-.02, .14]							
Item 5	.08* [.01, .15]	.08* [.02, .14]	-.03 [-.10, .04]	-.05 [-.11, .02]						
Item 6	-.03 [-.09, .03]	-.07* [-.12, -.02]	-.02 [-.08, .05]	.04 [-.02, .10]	-.04 [-.08, .00]					
Item 7	-.07* [-.14, -.01]	-.08* [-.13, -.03]	.04 [-.03, .11]	.01 [-.05, .08]	-.08* [-.13, -.03]	.09* [.05, .13]				
Item 8	-.06 [-.14, .01]	-.02 [-.08, .05]	.06 [-.02, .13]	.07 [.00, .15]	-.05 [-.11, .01]	.01 [-.04, .07]	.04 [-.02, .10]			
Item 9	-.02 [-.08, .06]	-.01 [-.06, .05]	-.01 [-.08, .06]	-.06 [-.13, .00]	.13* [.08, .17]	-.05* [-.09, -.01]	-.02 [-.07, .02]	-.02 [-.08, .04]		
Item 10	-.07 [-.14, .00]	-.08* [-.14, -.03]	.01 [-.05, .07]	-.01 [-.06, .05]	-.02 [-.06, .02]	.15* [.12, .18]	.10* [.06, .14]	.04 [-.02, .09]	.07* [.03, .10]	
Item 11	-.04 [-.12, .05]	-.03 [-.10, .05]	.13* [.05, .21]	.12* [.02, .22]	-.05 [-.11, .03]	.00 [-.06, .07]	.02 [-.06, .08]	.03 [-.05, .11]	-.05 [-.12, .02]	-.03 [-.09, .04]
Item 12	-.01 [-.10, .08]	-.01 [-.09, .07]	.05 [-.04, .13]	.07 [-.02, .15]	-.07 [-.14, .01]	.02 [-.04, .09]	.01 [-.06, .09]	.02 [-.07, .10]	-.16* [-.23, -.09]	-.05 [-.11, .02]
Item 13	-.08* [-.15, -.01]	-.04 [-.10, .02]	.06 [-.01, .13]	.04 [-.03, .11]	-.04 [-.10, .01]	.05* [.00, .09]	.14* [.09, .19]	.06 [.00, .13]	.02 [-.03, .07]	.09* [.05, .13]
Item 14	-.09* [-.16, -.02]	-.09* [-.14, -.03]	.01 [-.06, .08]	-.02 [-.09, .05]	-.05 [-.12, .01]	.10* [.05, .14]	.13* [.07, .17]	.04 [-.03, .10]	.01 [-.04, .06]	.14* [.10, .18]
Item 15	-.02 [-.10, .07]	.00 [-.08, .07]	.04 [-.05, .12]	.05 [-.03, .13]	-.08* [-.14, -.01]	.03 [-.03, .09]	.01 [-.06, .08]	.06 [-.02, .14]	-.17* [-.23, -.10]	-.08* [-.13, -.02]
Item 16	-.08* [-.14, -.02]	-.05* [-.10, .00]	.00 [-.06, .06]	.03 [-.03, .09]	-.06* [-.10, -.01]	.14* [.08, .18]	.12* [.08, .16]	.09* [.03, .15]	-.04 [-.08, .00]	.14* [.11, .17]
Item 17	.00 [-.07, .06]	.06* [.00, .11]	.02 [-.05, .09]	.03 [-.03, .10]	-.06* [-.10, -.01]	-.01 [-.04, .03]	-.01 [-.05, .04]	.03 [-.04, .10]	-.14* [-.19, -.10]	-.06* [-.09, -.02]
Item 18	.11* [.04, .17]	.14* [.08, .19]	.01 [-.06, .07]	-.02 [-.08, .04]	.19* [.13, .24]	-.05* [-.09, -.02]	-.11* [-.15, -.06]	-.06* [-.12, .00]	.09* [.05, .13]	-.06* [-.10, -.03]
Item 19	-.10* [-.17, -.02]	-.07* [-.14, .00]	.05 [-.04, .13]	.07 [-.02, .14]	-.08* [-.14, -.01]	.03 [-.03, .09]	.08* [.01, .14]	.06 [-.02, .13]	-.05 [-.11, .02]	.03 [-.03, .08]
Item 20	.10* [.02, .16]	.12* [.06, .17]	-.04 [-.11, .02]	-.03 [-.09, .04]	.08* [.03, .13]	-.03 [-.09, .02]	-.08* [-.13, -.04]	-.03 [-.09, .03]	.00 [-.05, .05]	-.08* [-.12, -.04]
Item 21	-.08* [-.16, .00]	-.07 [-.14, .00]	.10* [.02, .17]	.04 [-.04, .11]	-.01 [-.08, .05]	.06* [.00, .12]	.06 [-.01, .12]	.03 [-.04, .10]	.06 [-.02, .13]	.11* [.06, .17]
Item 22	-.04 [-.09, .03]	-.05* [-.10, .00]	-.04 [-.10, .03]	-.01 [-.07, .06]	-.02 [-.07, .02]	.12* [.08, .16]	.04 [-.01, .08]	.02 [-.04, .07]	-.07* [-.11, -.02]	.08* [.05, .12]
Item 23	-.11* [-.19, -.03]	-.10* [-.16, -.03]	.01 [-.07, .09]	-.01 [-.08, .07]	-.09* [-.15, -.03]	.03 [-.03, .09]	.08* [.02, .14]	.04 [-.04, .11]	.00 [-.07, .06]	.02 [-.04, .07]
Item 24	-.10* [-.18, -.02]	-.09* [-.16, -.02]	.05 [-.04, .13]	.02 [-.06, .10]	-.05 [-.12, .01]	.11* [.05, .16]	.11* [.05, .17]	.08 [.00, .15]	.02 [-.07, .09]	.16* [.11, .21]
Item 25	.09* [.00, .18]	.07 [-.01, .15]	.01 [-.08, .09]	-.01 [-.09, .07]	.08* [.01, .16]	-.08* [-.14, -.02]	-.09* [-.15, -.03]	.08 [-.02, .17]	.08* [.02, .15]	-.06 [-.12, .00]
Item 26	.09* [.01, .16]	.11* [.03, .18]	-.03 [-.10, .05]	-.01 [-.09, .06]	.05 [-.01, .11]	-.05* [-.11, .00]	-.04 [-.10, .02]	-.02 [-.09, .05]	.02 [-.04, .08]	-.04 [-.11, .02]

Item 27	.06 [-.01, .13]	.01 [-.05, .07]	-.06 [-.12, .01]	-.06 [-.13, .00]	.07* [.01, .12]	-.02 [-.06, .02]	-.01 [-.06, .04]	-.01 [-.07, .05]	.08* [.03, .12]	-.01 [-.05, .04]
Item 28	.01 [-.05, .08]	.01 [-.04, .07]	-.05 [-.12, .02]	-.07* [-.13, .00]	.02 [-.03, .07]	-.02 [-.06, .02]	-.01 [-.05, .04]	-.03 [-.09, .03]	.02 [-.03, .06]	.01 [-.03, .05]
Item 29	.07* [.00, .15]	.09* [.02, .15]	-.07 [-.14, .00]	-.05 [-.12, .02]	.11* [.05, .17]	-.05* [-.09, .00]	-.09* [-.15, -.03]	-.04 [-.10, .02]	.06* [.01, .11]	-.05* [-.10, -.01]
Item 30	-.11* [-.18, -.03]	-.08* [-.14, -.01]	.03 [-.04, .11]	.04 [-.04, .11]	-.04 [-.10, .02]	.05 [-.01, .10]	.19* [.12, .25]	.06 [-.01, .13]	.02 [-.04, .08]	.13* [.08, .17]
Item 31	.04 [-.04, .11]	.00 [-.07, .07]	-.10* [-.18, -.03]	-.07 [-.14, .00]	.03 [-.03, .09]	.01 [-.04, .06]	-.02 [-.08, .03]	-.05 [-.11, .02]	.06* [.00, .11]	.00 [-.04, .05]
Item 32	-.08* [-.14, -.01]	-.08* [-.13, -.03]	.02 [-.06, .10]	.12* [.05, .19]	-.08* [-.12, -.03]	.04 [.00, .09]	.06* [.01, .11]	.08* [.02, .14]	-.05* [-.10, -.01]	.00 [-.04, .04]
Item 33	-.10* [-.17, -.03]	-.11* [-.16, -.05]	.04 [-.03, .12]	.08* [.00, .15]	-.12* [-.17, -.07]	.05 [.00, .10]	.07* [.02, .13]	.09* [.02, .16]	-.06* [-.11, -.01]	.03 [-.01, .08]
Item 34	.07* [.00, .14]	.04 [-.02, .10]	-.04 [-.11, .03]	-.08* [-.14, -.01]	.11* [.05, .17]	-.02 [-.07, .02]	-.02 [-.07, .03]	-.05 [-.11, .01]	.08* [.04, .13]	.03 [-.01, .06]
Item 35	-.09* [-.15, -.03]	-.08* [-.12, -.02]	.18* [.11, .24]	.12* [.05, .18]	-.04 [-.09, .00]	.03 [-.01, .07]	.10* [.05, .14]	.06 [.00, .12]	-.02 [-.06, .03]	.02 [-.01, .06]
Item 36	-.01 [-.07, .06]	-.05 [-.10, .01]	.19* [.12, .26]	.04 [-.04, .12]	.05* [.00, .10]	.00 [-.04, .04]	.03 [-.02, .08]	.02 [-.04, .08]	.05* [.00, .10]	.04 [.00, .08]
Item 37	.20* [.13, .27]	.16* [.09, .22]	-.03 [-.10, .04]	-.01 [-.08, .06]	.00 [-.06, .06]	-.03 [-.07, .02]	-.08* [-.13, -.03]	-.06 [-.13, .00]	-.14* [-.19, -.08]	-.12* [-.16, -.07]
Item 38	.04 [-.03, .12]	.02 [-.04, .08]	-.02 [-.09, .05]	-.04 [-.11, .03]	.04 [-.02, .09]	-.03 [-.07, .02]	-.07* [-.12, -.02]	.05 [-.01, .11]	.09* [.04, .14]	-.02 [-.06, .02]
Item 39	-.01 [-.09, .07]	.07 [.00, .14]	.02 [-.06, .10]	.02 [-.06, .10]	-.06 [-.12, .01]	-.01 [-.07, .05]	.01 [-.05, .08]	.01 [-.06, .09]	-.14* [-.20, -.07]	-.05 [-.11, .01]
Item 40	.08* [.01, .14]	.11* [.05, .17]	-.01 [-.08, .05]	-.04 [-.10, .03]	.14* [.09, .19]	-.05* [-.09, -.01]	-.04 [-.10, .02]	-.07* [-.12, -.01]	.08* [.03, .12]	-.06* [-.10, -.02]
Item 41	-.09* [-.16, -.01]	-.11* [-.18, -.04]	.01 [-.07, .09]	.01 [-.07, .09]	-.05 [-.11, .01]	.12* [.06, .17]	.09* [.03, .15]	.06 [-.01, .13]	.05 [-.03, .11]	.16* [.11, .20]
Item 42	.10* [.03, .16]	.12* [.06, .17]	-.06 [-.12, .01]	-.03 [-.09, .03]	.10* [.05, .14]	-.03 [-.06, .01]	-.11* [-.15, -.07]	-.10* [-.17, -.03]	.00 [-.04, .04]	-.09* [-.12, -.06]
Item 43	.12* [.04, .20]	.02 [-.06, .10]	-.07 [-.15, .01]	-.04 [-.12, .04]	.03 [-.04, .09]	.03 [-.03, .09]	-.07* [-.13, -.01]	-.03 [-.10, .04]	-.01 [-.08, .05]	-.03 [-.09, .04]
Item 44	.06 [-.02, .14]	.06 [-.01, .13]	.01 [-.06, .08]	-.06 [-.13, .01]	.14* [.07, .19]	-.07* [-.12, -.02]	.01 [-.07, .07]	-.05 [-.12, .01]	.10* [.04, .15]	-.03 [-.07, .02]
Item 45	-.08* [-.15, -.01]	-.07* [-.13, -.01]	.02 [-.06, .09]	.02 [-.05, .09]	-.08* [-.13, -.03]	.07* [.02, .11]	.07* [.01, .12]	.01 [-.06, .07]	-.04 [-.09, .01]	.03 [-.02, .07]
Item 46	.10* [.03, .16]	.04 [-.02, .10]	-.05 [-.11, .02]	-.09* [-.15, -.02]	.10* [.05, .16]	-.05* [-.09, -.01]	-.07* [-.12, -.02]	-.04 [-.09, .03]	.07* [.03, .12]	-.03 [-.07, .01]
Item 47	-.05 [-.12, .02]	-.04 [-.10, .02]	.08 [.00, .15]	.09* [.01, .16]	-.08* [-.13, -.02]	.00 [-.05, .05]	.04 [-.02, .09]	.04 [-.03, .11]	-.07* [-.12, -.02]	-.02 [-.07, .03]
Item 48	-.06 [-.12, .00]	-.06* [-.12, -.01]	-.02 [-.08, .04]	.02 [-.04, .08]	-.03 [-.08, .01]	.13* [.08, .17]	.09* [.04, .13]	.05 [-.01, .11]	.01 [-.03, .05]	.19* [.15, .22]
Item 49	.16* [.08, .23]	.10* [.03, .16]	-.07 [-.14, .01]	-.04 [-.11, .03]	.10* [.04, .16]	-.07* [-.14, -.01]	-.09* [-.14, -.04]	-.05 [-.11, .02]	.05 [-.01, .10]	-.04 [-.09, .01]
Item 50	-.04 [-.10, .03]	-.01 [-.06, .05]	.01 [-.06, .08]	.02 [-.05, .09]	-.05 [-.10, .00]	.08* [.03, .12]	.03 [-.02, .08]	.03 [-.03, .09]	-.13* [-.17, -.08]	.02 [-.02, .07]
	Item 11	Item 12	Item 13	Item 14	Item 15	Item 16	Item 17	Item 18	Item 19	Item 20
Item 12	.08 [-.01, .17]									
Item 13	.05 [-.03, .12]	.02 [-.06, .10]								
Item 14	-.01 [-.08, .07]	-.01 [-.08, .07]	.14* [.09, .20]							
Item 15	.09* [.01, .18]	.14* [.06, .23]	.00 [-.06, .08]	-.03 [-.09, .05]						
Item 16	-.01 [-.08, .05]	.00 [-.07, .06]	.09* [.04, .13]	.12* [.07, .16]	.02 [-.04, .08]					
Item 17	.04 [-.03, .10]	.13* [.05, .20]	.00 [-.05, .05]	-.02 [-.06, .03]	.06 [-.01, .13]	-.03 [-.07, .01]				
Item 18	-.01 [-.07, .06]	-.04 [-.11, .03]	-.05 [-.10, .01]	-.07* [-.13, -.02]	-.04 [-.10, .02]	-.12* [-.16, -.09]	-.04* [-.08, .00]			
Item 19	.04 [-.04, .13]	.05 [-.03, .14]	.06 [-.01, .13]	.07 [.00, .13]	.03 [-.05, .11]	.07* [.01, .13]	.05 [-.01, .11]	-.11* [-.16, -.05]		
Item 20	-.03 [-.09, .04]	-.02 [-.09, .05]	-.08* [-.13, -.04]	-.06* [-.11, -.02]	-.02 [-.08, .05]	-.06* [-.12, -.01]	-.01 [-.05, .04]	.10* [.06, .15]	-.07* [-.13, .00]	
Item 21	.04 [-.04, .12]	.01 [-.07, .10]	.06 [.00, .13]	.08* [.01, .14]	.01 [-.07, .09]	.05 [-.01, .11]	-.01 [-.07, .05]	-.03 [-.09, .02]	.05 [-.03, .12]	-.10* [-.16, -.04]
Item 22	-.04 [-.11, .02]	.03 [-.04, .10]	.02 [-.03, .06]	.07* [.03, .12]	.03 [-.03, .09]	.04 [.00, .08]	.05* [.01, .09]	-.07* [-.11, -.03]	.04 [-.02, .10]	-.03 [-.07, .01]
Item 23	.02 [-.06, .10]	.00 [-.09, .08]	.06 [-.01, .13]	.08* [.01, .14]	.02 [-.06, .10]	.03 [-.03, .09]	.00 [-.06, .06]	-.12* [-.17, -.07]	.12* [.04, .20]	-.08* [-.14, -.02]

Item 24	.01 [-.08, .09]	-.01 [-.09, .08]	.10* [.04, .17]	.09* [.03, .16]	.00 [-.08, .08]	.11* [.05, .17]	-.03 [-.09, .03]	-.09* [-.15, -.03]	.08* [.00, .16]	-.10* [-.16, -.04]
Item 25	.02 [-.07, .10]	-.05 [-.14, .04]	-.05 [-.12, .02]	-.07 [-.13, .00]	.01 [-.07, .09]	-.07* [-.13, -.01]	-.08* [-.17, .00]	.10* [.03, .16]	-.06 [-.14, .02]	.07 [-.01, .14]
Item 26	-.03 [-.10, .05]	.02 [-.06, .10]	-.04 [-.10, .02]	-.06* [-.12, .00]	-.02 [-.09, .06]	-.05 [-.10, .00]	-.01 [-.06, .05]	.08* [.03, .14]	-.05 [-.12, .03]	.10* [.04, .15]
Item 27	-.06 [-.13, .01]	-.07 [-.14, .00]	-.04 [-.09, .01]	-.02 [-.07, .03]	-.09* [-.16, -.03]	-.05* [-.09, .00]	-.05* [-.09, .00]	.02 [-.03, .07]	-.05 [-.12, .03]	.03 [-.02, .08]
Item 28	-.08* [-.14, -.01]	-.01 [-.09, .06]	-.04 [-.09, .00]	.04 [-.01, .09]	-.05 [-.11, .02]	.01 [-.03, .05]	.01 [-.03, .06]	.01 [-.04, .05]	.00 [-.06, .06]	.02 [-.03, .06]
Item 29	-.03 [-.10, .05]	-.07 [-.14, .01]	-.03 [-.09, .02]	-.04 [-.09, .02]	-.05 [-.12, .02]	-.06* [-.11, -.02]	-.03 [-.07, .03]	.10* [.04, .15]	-.06 [-.12, .01]	.05 [.00, .11]
Item 30	.02 [-.06, .09]	-.01 [-.09, .08]	.21* [.14, .26]	.19* [.13, .25]	-.01 [-.09, .07]	.08* [.03, .14]	-.04 [-.09, .02]	-.09* [-.14, -.03]	.09* [.01, .17]	-.12* [-.17, -.06]
Item 31	-.08* [-.16, .00]	-.06 [-.14, .02]	-.04 [-.10, .01]	.03 [-.03, .08]	-.06 [-.13, .02]	.00 [-.05, .05]	-.02 [-.08, .03]	.02 [-.04, .07]	-.03 [-.09, .04]	.03 [-.03, .09]
Item 32	.04 [-.04, .12]	.02 [-.05, .09]	.05 [.00, .11]	.04 [-.02, .09]	.03 [-.04, .10]	.07* [.02, .11]	.06* [.01, .10]	-.09* [-.14, -.05]	.17* [.11, .23]	-.05* [-.10, -.01]
Item 33	.04 [-.04, .12]	.03 [-.05, .10]	.04 [-.02, .10]	.06 [.00, .12]	.09* [.02, .16]	.08* [.03, .13]	.04 [-.01, .09]	-.14* [-.19, -.09]	.16* [.08, .24]	-.09* [-.14, -.03]
Item 34	-.07 [-.13, .01]	-.05 [-.12, .02]	-.07* [-.12, -.01]	-.02 [-.08, .04]	-.09* [-.15, -.02]	-.05* [-.10, -.01]	-.06* [-.10, -.01]	.08* [.02, .13]	-.08* [-.14, -.02]	.05 [.00, .09]
Item 35	.14* [.07, .20]	.04 [-.03, .11]	.11* [.06, .16]	.05* [.00, .10]	.04 [-.03, .10]	.04 [.00, .08]	.01 [-.03, .05]	-.07* [-.11, -.03]	.07 [-.01, .14]	-.07* [-.11, -.03]
Item 36	.04 [-.05, .12]	.00 [-.07, .07]	.04 [-.01, .09]	-.01 [-.05, .04]	-.01 [-.08, .05]	-.01 [-.05, .03]	-.04 [-.08, .01]	.04 [-.01, .08]	.01 [-.06, .07]	-.02 [-.07, .03]
Item 37	-.02 [-.09, .06]	.04 [-.05, .13]	-.10* [-.15, -.04]	-.10* [-.15, -.04]	.04 [-.05, .12]	-.09* [-.14, -.04]	.12* [.06, .17]	.06* [.00, .11]	-.06 [-.13, .01]	.09* [.03, .14]
Item 38	-.02 [-.09, .05]	-.06 [-.14, .01]	-.05 [-.10, .00]	-.04 [-.09, .01]	-.01 [-.07, .06]	-.02 [-.07, .03]	-.07* [-.13, -.01]	.05 [.00, .10]	-.05 [-.11, .01]	.04 [-.01, .09]
Item 39	.02 [-.07, .10]	.14* [.05, .23]	.00 [-.07, .07]	.01 [-.06, .08]	.11* [.02, .20]	-.01 [-.06, .05]	.23* [.17, .29]	-.03 [-.09, .03]	.03 [-.05, .11]	-.02 [-.08, .05]
Item 40	-.03 [-.10, .04]	-.05 [-.12, .02]	-.04 [-.09, .01]	-.05* [-.10, .00]	-.06 [-.12, .01]	-.08* [-.12, -.03]	-.07* [-.11, -.02]	.18* [.13, .22]	-.10* [-.16, -.04]	.11* [.06, .16]
Item 41	-.02 [-.10, .06]	-.04 [-.12, .05]	.09* [.03, .15]	.12* [.05, .18]	-.03 [-.11, .05]	.16* [.11, .21]	-.02 [-.08, .04]	-.08* [-.14, -.03]	.02 [-.06, .10]	-.07* [-.13, -.01]
Item 42	.05 [-.02, .11]	.01 [-.06, .08]	-.07* [-.11, -.03]	-.08* [-.13, -.04]	.02 [-.05, .08]	-.10* [-.14, -.07]	.06* [.01, .10]	.17* [.13, .21]	-.08* [-.13, -.02]	.08* [.04, .13]
Item 43	-.05 [-.13, .03]	-.02 [-.11, .07]	-.08* [-.14, -.01]	-.07 [-.13, .00]	.01 [-.07, .09]	.01 [-.05, .07]	-.02 [-.08, .05]	.02 [-.04, .08]	-.06 [-.13, .02]	.11* [.05, .17]
Item 44	-.02 [-.09, .06]	-.05 [-.13, .03]	.03 [-.03, .09]	-.02 [-.08, .04]	-.04 [-.11, .03]	-.08* [-.13, -.03]	-.10* [-.15, -.04]	.14* [.08, .19]	-.08* [-.15, -.01]	.05 [-.01, .11]
Item 45	.04 [-.04, .11]	.04 [-.04, .11]	.03 [-.03, .09]	.07* [.02, .13]	.02 [-.05, .09]	.06* [.01, .10]	.06* [.01, .11]	-.10* [-.14, -.05]	.12* [.05, .19]	-.07* [-.12, -.02]
Item 46	-.05 [-.12, .02]	-.06 [-.13, .01]	-.06* [-.11, -.01]	-.03 [-.08, .02]	-.06 [-.12, .01]	-.07* [-.11, -.03]	-.05* [-.09, -.01]	.08* [.03, .13]	-.06 [-.12, .00]	.06* [.01, .11]
Item 47	.07 [-.01, .15]	.10* [.02, .17]	.04 [-.02, .10]	.01 [-.05, .07]	.05 [-.02, .12]	.04 [-.01, .09]	.11* [.06, .16]	-.06* [-.11, -.01]	.17* [.10, .24]	-.02 [-.07, .03]
Item 48	-.04 [-.11, .02]	.01 [-.06, .08]	.06* [.01, .11]	.12* [.08, .17]	-.03 [-.09, .03]	.20* [.16, .24]	.01 [-.04, .05]	-.06* [-.10, -.02]	.02 [-.04, .09]	-.06* [-.11, -.01]
Item 49	-.06 [-.13, .02]	-.07 [-.14, .01]	-.10* [-.16, -.04]	-.08* [-.13, -.02]	-.04 [-.11, .03]	-.06 [-.13, .01]	-.05 [-.10, .00]	.09* [.03, .14]	-.08* [-.14, -.01]	.10* [.02, .17]
Item 50	.00 [-.07, .07]	.09* [.01, .17]	.02 [-.03, .08]	.03 [-.02, .08]	.04 [-.04, .11]	.01 [-.04, .05]	.16* [.11, .20]	-.04 [-.09, .00]	.06* [.00, .13]	-.03 [-.08, .01]

	Item 21	Item 22	Item 23	Item 24	Item 25	Item 26	Item 27	Item 28	Item 29	Item 30
Item 22	.06 [-.01, .13]									
Item 23	.05 [-.02, .13]	.06 [.00, .11]								
Item 24	.13* [.06, .20]	.08* [.01, .16]	.08 [.00, .15]							
Item 25	-.02 [-.10, .06]	-.06 [-.12, .01]	-.04 [-.12, .04]	-.05 [-.14, .03]						
Item 26	-.06 [-.13, .01]	-.05 [-.10, .01]	-.08* [-.15, -.01]	-.07 [-.14, .01]	.04 [-.04, .12]					
Item 27	-.03 [-.09, .04]	.00 [-.05, .04]	.01 [-.05, .08]	-.04 [-.10, .03]	.07 [.00, .14]	.03 [-.03, .09]				
Item 28	-.02 [-.09, .04]	-.03 [-.07, .02]	-.04 [-.11, .03]	-.06 [-.12, .01]	.01 [-.06, .07]	.00 [-.05, .06]	.08* [.03, .13]			
Item 29	-.06 [-.13, .01]	-.02 [-.07, .02]	-.02 [-.09, .04]	-.07* [-.14, .00]	.08* [.00, .15]	.02 [-.04, .09]	.12* [.06, .17]	.02 [-.03, .08]		
Item 30	.08* [.01, .15]	.05 [-.01, .10]	.16* [.09, .23]	.12* [.04, .18]	-.09* [-.16, -.01]	-.07 [-.14, .00]	.00 [-.06, .06]	-.01 [-.06, .05]	-.07* [-.13, .00]	

Item 31	-.04 [-.11, .03]	.05 [.00, .10]	-.03 [-.10, .05]	-.04 [-.11, .03]	.03 [-.05, .11]	.00 [-.07, .07]	.16* [.10, .22]	.20* [.14, .26]	.08* [.01, .14]	-.03 [-.10, .03]
Item 32	.03 [-.04, .09]	.08* [.04, .13]	.11* [.05, .17]	.05 [-.02, .11]	-.06 [-.12, .01]	-.06* [-.12, -.01]	.01 [-.04, .06]	-.02 [-.07, .02]	-.04 [-.09, .02]	.08* [.01, .13]
Item 33	.05 [-.02, .12]	.05* [.00, .10]	.21* [.14, .27]	.08* [.01, .15]	-.06 [-.13, .01]	-.08* [-.15, -.02]	-.04 [-.10, .02]	.03 [-.02, .08]	-.05 [-.11, .01]	.12* [.05, .18]
Item 34	-.03 [-.09, .04]	.01 [-.03, .05]	-.06 [-.12, .00]	-.04 [-.10, .03]	.06 [-.02, .13]	.04 [-.02, .10]	.10* [.04, .15]	.04 [-.01, .09]	.10* [.04, .15]	-.01 [-.07, .05]
Item 35	.11* [.05, .16]	.01 [-.03, .05]	.06* [.01, .12]	.08* [.02, .14]	-.03 [-.09, .04]	-.04 [-.09, .02]	-.07* [-.12, -.02]	-.06* [-.10, -.01]	-.08* [-.13, -.03]	.12* [.06, .17]
Item 36	.05 [-.01, .11]	-.02 [-.06, .03]	-.02 [-.08, .04]	.04 [-.03, .10]	.02 [-.04, .09]	.01 [-.05, .06]	-.01 [-.05, .05]	-.09* [-.14, -.04]	-.02 [-.07, .03]	-.02 [-.07, .04]
Item 37	-.09* [-.16, -.02]	.01 [-.04, .06]	-.07* [-.14, .00]	-.11* [-.17, -.03]	.01 [-.06, .09]	.10* [.03, .17]	.03 [-.03, .09]	.00 [-.05, .06]	.06* [.00, .12]	-.13* [-.19, -.06]
Item 38	-.01 [-.08, .06]	-.03 [-.07, .02]	-.02 [-.08, .04]	-.04 [-.10, .03]	.15* [.09, .21]	.02 [-.05, .08]	.07* [.02, .12]	.02 [-.03, .07]	.08* [.02, .14]	-.02 [-.08, .04]
Item 39	-.02 [-.10, .06]	.03 [-.03, .09]	-.01 [-.09, .07]	-.01 [-.09, .08]	-.07 [-.15, .01]	.01 [-.07, .09]	-.07* [-.14, -.01]	.02 [-.04, .09]	-.05 [-.11, .02]	-.02 [-.10, .06]
Item 40	-.06 [-.12, .01]	-.08* [-.12, -.03]	-.07* [-.12, -.01]	-.05 [-.12, .01]	.07 [-.01, .14]	.07* [.01, .13]	.05 [.00, .10]	.00 [-.05, .05]	.10* [.04, .16]	-.08* [-.14, -.01]
Item 41	.08* [.00, .15]	.07 [.00, .14]	.07 [-.01, .14]	.17* [.08, .25]	-.05 [-.13, .03]	-.07 [-.14, .01]	-.02 [-.08, .04]	.01 [-.05, .07]	-.05 [-.11, .02]	.14* [.07, .20]
Item 42	-.06 [-.11, .01]	-.03 [-.07, .01]	-.07* [-.13, -.02]	-.10* [-.16, -.04]	.06 [-.03, .14]	.04 [-.02, .10]	-.01 [-.05, .04]	.01 [-.03, .05]	.09* [.04, .15]	-.12* [-.17, -.07]
Item 43	-.07 [-.15, .01]	.00 [-.06, .06]	-.07 [-.15, .01]	-.08 [-.15, .01]	.06 [-.03, .14]	.08 [.00, .16]	.06 [-.01, .12]	.07* [.01, .13]	.03 [-.04, .10]	-.09* [-.16, -.01]
Item 44	.00 [-.07, .07]	-.05 [-.10, .01]	-.02 [-.09, .05]	-.04 [-.11, .03]	.08* [.00, .16]	.06 [-.01, .13]	.04 [-.02, .10]	.00 [-.06, .05]	.06 [-.01, .12]	.00 [-.08, .08]
Item 45	.02 [-.04, .09]	.07* [.02, .11]	.28* [.21, .35]	.06 [.00, .13]	-.07 [-.14, .00]	-.07* [-.13, .00]	-.04 [-.09, .01]	-.04 [-.10, .02]	-.04 [-.09, .02]	.09* [.03, .15]
Item 46	-.04 [-.10, .02]	-.01 [-.06, .03]	-.06 [-.13, .01]	-.07* [-.13, .00]	.08* [.00, .15]	.05 [-.01, .11]	.24* [.19, .29]	.05 [-.01, .10]	.12* [.06, .17]	-.06* [-.12, .00]
Item 47	.02 [-.05, .10]	.01 [-.04, .06]	.05 [-.01, .12]	.02 [-.05, .09]	-.07 [-.14, .01]	-.03 [-.10, .03]	-.07* [-.13, -.01]	.01 [-.05, .06]	-.06* [-.12, -.01]	.02 [-.05, .09]
Item 48	.04 [-.02, .11]	.10* [.06, .14]	.01 [-.05, .07]	.10* [.04, .16]	-.07* [-.13, .00]	-.03 [-.09, .02]	-.01 [-.05, .04]	.00 [-.04, .04]	-.05 [-.10, .00]	.08* [.02, .14]
Item 49	-.06 [-.13, .02]	-.04 [-.09, .01]	-.08* [-.14, -.01]	-.08* [-.16, -.01]	.12* [.04, .20]	.10* [.03, .17]	.07* [.01, .13]	.01 [-.05, .06]	.09* [.03, .16]	-.10* [-.16, -.03]
Item 50	.06 [-.01, .12]	.18* [.14, .23]	.02 [-.05, .08]	.05 [-.02, .12]	-.09* [-.16, -.02]	.00 [-.05, .06]	-.07* [-.12, -.02]	-.03 [-.08, .02]	-.03 [-.08, .02]	.02 [-.04, .08]
	Item 31	Item 32	Item 33	Item 34	Item 35	Item 36	Item 37	Item 38	Item 39	Item 40
Item 32	-.02 [-.07, .04]									
Item 33	.01 [-.06, .07]	.24* [.18, .29]								
Item 34	.08* [.02, .14]	-.07* [-.12, -.03]	-.10* [-.15, -.05]							
Item 35	-.08* [-.13, -.03]	.09* [.04, .14]	.12* [.06, .18]	-.08* [-.12, -.03]						
Item 36	-.08* [-.14, -.02]	-.02 [-.08, .03]	-.02 [-.07, .04]	.01 [-.04, .06]	.18* [.14, .22]					
Item 37	.01 [-.05, .08]	-.01 [-.07, .04]	-.06* [-.12, .00]	.02 [-.04, .07]	-.09* [-.14, -.04]	-.06* [-.11, .00]				
Item 38	.08* [.02, .14]	-.05 [-.09, .01]	-.02 [-.07, .04]	.03 [-.02, .08]	-.06* [-.11, -.01]	-.02 [-.07, .03]	.00 [-.06, .06]			
Item 39	-.03 [-.11, .04]	.02 [-.05, .08]	.01 [-.07, .07]	-.06 [-.12, .00]	.01 [-.05, .07]	-.08* [-.14, -.02]	.08 [.00, .16]	-.09* [-.16, -.02]		
Item 40	.02 [-.04, .08]	-.09* [-.14, -.05]	-.12* [-.17, -.07]	.10* [.05, .15]	-.07* [-.11, -.02]	.02 [-.03, .07]	.04 [-.02, .10]	.05 [.00, .10]	-.05 [-.11, .02]	
Item 41	-.01 [-.08, .06]	.02 [-.04, .08]	.07 [.00, .13]	-.01 [-.07, .05]	.05 [-.01, .11]	.00 [-.06, .06]	-.10* [-.17, -.03]	-.01 [-.07, .06]	-.04 [-.11, .05]	-.07* [-.13, -.01]
Item 42	.06* [.01, .11]	-.07* [-.11, -.02]	-.07* [-.12, -.02]	.07* [.03, .12]	-.11* [-.15, -.07]	-.07* [-.11, -.03]	.15* [.10, .20]	.05 [-.01, .11]	.04 [-.02, .10]	.09* [.05, .14]
Item 43	.06 [-.01, .13]	-.03 [-.09, .03]	-.06 [-.12, .02]	.04 [-.02, .10]	-.08* [-.14, -.02]	-.05 [-.11, .01]	.09* [.02, .17]	.03 [-.04, .09]	-.01 [-.09, .08]	.04 [-.02, .10]
Item 44	.02 [-.05, .09]	-.09* [-.14, -.03]	-.12* [-.18, -.06]	.12* [.06, .17]	-.05 [-.10, .01]	.02 [-.04, .07]	.00 [-.06, .07]	.06 [.00, .12]	-.06 [-.13, .01]	.15* [.08, .21]
Item 45	-.08* [-.14, -.01]	.11* [.06, .17]	.17* [.11, .22]	-.05 [-.10, .00]	.04 [-.01, .09]	-.05 [-.10, .00]	-.01 [-.07, .05]	-.10* [-.15, -.04]	.05 [-.02, .11]	-.07* [-.12, -.02]
Item 46	.13* [.06, .20]	-.05 [-.09, .00]	-.08* [-.13, -.03]	.12* [.07, .17]	-.07* [-.11, -.02]	.00 [-.05, .05]	.05 [-.01, .11]	.06* [.00, .11]	-.07* [-.13, -.01]	.07* [.02, .12]
Item 47	-.04 [-.09, .03]	.11* [.05, .16]	.06 [-.01, .13]	-.08* [-.13, -.03]	.06 [.00, .12]	.03 [-.03, .08]	-.01 [-.07, .05]	-.05 [-.11, .00]	.12* [.05, .19]	-.06* [-.11, -.01]

Item 48	.02 [-.04, .07]	.05* [.00, .10]	.03 [-.02, .08]	.01 [-.04, .06]	.01 [-.03, .06]	-.01 [-.06, .03]	-.10* [-.15, -.05]	-.01 [-.06, .04]	.00 [-.06, .06]	-.05* [-.09, -.01]
Item 49	.03 [-.03, .10]	-.10* [-.15, -.04]	-.11* [-.16, -.05]	.11* [.05, .16]	-.11* [-.16, -.05]	.00 [-.05, .06]	.09* [.03, .16]	.05 [-.01, .11]	-.05 [-.12, .02]	.12* [.06, .18]
Item 50	-.05 [-.11, .01]	.05* [.00, .10]	.04 [-.01, .10]	-.03 [-.08, .02]	.02 [-.03, .06]	.00 [-.05, .05]	.05 [-.01, .11]	-.08* [-.13, -.02]	.10* [.02, .17]	-.06* [-.10, -.01]
	Item 41	Item 42	Item 43	Item 44	Item 45	Item 46	Item 47	Item 48	Item 49	Item 50
Item 42	-.09* [-.15, -.04]									
Item 43	-.03 [-.10, .05]	.04 [-.02, .10]								
Item 44	-.03 [-.10, .04]	.07* [.01, .12]	.02 [-.05, .09]							
Item 45	.07* [.00, .13]	-.01 [-.05, .04]	-.05 [-.12, .01]	-.06* [-.12, .00]						
Item 46	-.06* [-.12, .00]	.03 [-.02, .07]	.02 [-.04, .09]	.07* [.00, .12]	-.10* [-.16, -.04]					
Item 47	-.01 [-.08, .07]	-.05* [-.10, .00]	-.04 [-.11, .03]	-.05 [-.11, .01]	.06* [.00, .12]	-.05 [-.10, .01]				
Item 48	.15* [.10, .21]	-.06* [-.10, -.03]	.03 [-.03, .09]	-.04 [-.09, .02]	.04 [-.01, .09]	-.04 [-.08, .01]	-.01 [-.06, .05]			
Item 49	-.06 [-.13, .01]	.08* [.03, .14]	.16* [.09, .23]	.09* [.02, .16]	-.09* [-.14, -.03]	.09* [.03, .14]	-.06* [-.12, .00]	-.03 [-.09, .03]		
Item 50	.05 [-.02, .11]	.00 [-.05, .04]	-.03 [-.09, .03]	-.07* [-.13, -.02]	.03 [-.02, .09]	-.06* [-.11, -.02]	.07* [.01, .12]	.04 [-.01, .08]	-.05 [-.10, .00]	

Sample C: Spanish

First-order factor loadings	Identification-self	Comprehension-self	Expression-self	Regulation-self	Utilization-self	Identification-other	Comprehension-other	Expression-other	Regulation-other	Utilization-other
Item 6	**.32* [.10, .54]**	.03 [-.10, .17]	.01 [-.09, .11]	-.02 [-.13, .10]	.06 [-.03, .16]	.03 [-.08, .14]	.02 [-.09, .13]	.01 [-.09, .11]	.03 [-.10, .15]	.01 [-.08, .10]
Item 16	**.58* [.38, .78]**	.09 [-.04, .22]	.03 [-.07, .12]	-.01 [-.12, .10]	.03 [-.07, .11]	.02 [-.09, .13]	.00 [-.11, .10]	.00 [-.10, .10]	-.03 [-.15, .09]	-.02 [-.11, .07]
Item 20	**.43* [.21, .63]**	.03 [-.11, .16]	-.03 [-.13, .06]	.03 [-.08, .14]	-.08 [-.17, .01]	-.03 [-.14, .08]	-.02 [-.13, .08]	-.02 [-.12, .08]	-.01 [-.13, .11]	.03 [-.06, .12]
Item 48	**.59* [.42, .77]**	.01 [-.12, .14]	-.01 [-.11, .08]	.02 [-.09, .13]	.05 [-.04, .14]	-.01 [-.12, .10]	.00 [-.11, .10]	.02 [-.08, .12]	.02 [-.10, .14]	-.01 [-.10, .08]
Item 49	**.51* [.30, .70]**	.09 [-.04, .22]	.02 [-.07, .11]	-.01 [-.12, .10]	-.03 [-.12, .06]	.02 [-.09, .13]	-.01 [-.11, .10]	.00 [-.09, .10]	-.01 [-.13, .11]	.01 [-.08, .10]
Item 1	-.04 [-.15, .06]	**.77* [.65, .90]**	-.03 [-.12, .06]	.00 [-.11, .11]	-.06 [-.15, .03]	-.03 [-.14, .07]	-.02 [-.13, .08]	-.01 [-.10, .09]	-.01 [-.13, .11]	-.03 [-.12, .06]
Item 2	-.01 [-.12, .10]	**.61* [.46, .76]**	-.01 [-.10, .09]	.02 [-.09, .13]	-.03 [-.12, .06]	-.01 [-.11, .10]	.01 [-.10, .11]	-.01 [-.11, .09]	.02 [-.10, .14]	-.06 [-.15, .03]
Item 10	.07 [-.03, .20]	**.47* [.32, .62]**	.04 [-.05, .14]	.01 [-.09, .12]	-.01 [-.09, .08]	.01 [-.09, .12]	.03 [-.07, .14]	.00 [-.10, .09]	.00 [-.12, .11]	-.02 [-.10, .07]
Item 26	.04 [-.07, .16]	**.44* [.28, .60]**	.03 [-.07, .12]	.06 [-.05, .17]	-.04 [-.14, .05]	.00 [-.11, .11]	.01 [-.10, .11]	-.02 [-.12, .07]	-.02 [-.13, .11]	.01 [-.08, .10]
Item 43	.01 [-.10, .12]	**.66* [.51, .80]**	-.02 [-.11, .07]	.03 [-.07, .14]	.01 [-.08, .10]	.01 [-.10, .11]	-.03 [-.14, .08]	-.02 [-.12, .08]	-.01 [-.13, .11]	.02 [-.07, .11]
Item 8	.02 [-.09, .13]	.01 [-.11, .12]	**.59* [.43, .76]**	.01 [-.10, .11]	.05 [-.04, .14]	.02 [-.09, .13]	.03 [-.07, .14]	.00 [-.10, .09]	.01 [-.10, .13]	.04 [-.05, .13]
Item 17	.04 [-.07, .15]	.01 [-.11, .13]	**.38* [.21, .55]**	.16* [.05, .27]	.01 [-.08, .10]	-.01 [-.12, .09]	.01 [-.09, .12]	.02 [-.07, .12]	.05 [-.06, .17]	.00 [-.09, .09]
Item 25	-.02 [-.13, .09]	-.02 [-.14, .10]	**.70* [.53, .86]**	-.06 [-.17, .05]	-.03 [-.12, .07]	-.01 [-.11, .10]	.00 [-.11, .10]	-.02 [-.12, .08]	.01 [-.12, .12]	.01 [-.08, .10]
Item 38	-.02 [-.13, .08]	-.04 [-.16, .07]	**.53* [.42, .67]**	-.07 [-.18, .04]	.06 [-.03, .16]	.00 [-.11, .11]	-.01 [-.11, .10]	.02 [-.07, .12]	-.01 [-.13, .11]	-.01 [-.11, .08]
Item 42	-.01 [-.11, .10]	.05 [-.07, .16]	**.20* [.02, .38]**	.05 [-.06, .15]	.03 [-.06, .12]	.05 [-.06, .16]	.01 [-.09, .12]	.00 [-.09, .10]	.05 [-.07, .17]	-.05 [-.14, .04]
Item 12	-.02 [-.13, .09]	-.03 [-.15, .09]	-.02 [-.11, .08]	**.80* [.71, .92]**	-.02 [-.11, .08]	-.02 [-.13, .09]	-.02 [-.12, .09]	-.01 [-.11, .09]	-.04 [-.16, .08]	.05 [-.04, .15]
Item 15	-.01 [-.11, .10]	-.02 [-.13, .10]	-.01 [-.10, .08]	**.59* [.46, .72]**	-.03 [-.13, .06]	.01 [-.10, .12]	.00 [-.10, .11]	-.01 [-.11, .09]	.01 [-.12, .13]	.04 [-.06, .13]
Item 37	.09 [-.01, .21]	.20* [.09, .32]	.08 [-.01, .17]	**.27* [.15, .40]**	-.02 [-.10, .07]	-.01 [-.11, .10]	.01 [-.09, .12]	-.01 [-.10, .08]	-.05 [-.16, .07]	.00 [-.08, .09]
Item 39	-.02 [-.13, .08]	-.02 [-.14, .10]	-.02 [-.11, .08]	**.68* [.55, .80]**	-.01 [-.10, .08]	-.03 [-.15, .07]	-.02 [-.13, .08]	-.03 [-.13, .07]	-.01 [-.13, .11]	-.04 [-.13, .05]
Item 50	.02 [-.09, .13]	.04 [-.07, .16]	.02 [-.08, .11]	**.37* [.23, .50]**	.08 [-.01, .17]	.02 [-.08, .13]	.02 [-.08, .12]	.01 [-.09, .11]	.02 [-.10, .14]	.00 [-.09, .09]
Item 9	-.01 [-.11, .09]	-.10 [-.22, .01]	.01 [-.08, .11]	-.11* [-.22, .00]	**.40* [.28, .52]**	.02 [-.08, .13]	.03 [-.07, .13]	.02 [-.08, .12]	-.02 [-.14, .10]	.01 [-.08, .10]

Item 21	.00 [-.11, .11]	-.02 [-.14, .10]	.03 [-.07, .12]	.02 [-.09, .13]	**.67* [.60, .76]**	.00 [-.10, .11]	.00 [-.10, .11]	.00 [-.09, .10]	.04 [-.08, .16]	-.03 [-.12, .06]
Item 22	.04 [-.06, .15]	.06 [-.05, .18]	.01 [-.08, .10]	.09 [-.02, .19]	**.32* [.20, .44]**	.06 [-.04, .17]	.05 [-.05, .16]	.04 [-.05, .15]	.06 [-.06, .18]	-.04 [-.13, .04]
Item 24	.00 [-.10, .11]	.00 [-.12, .12]	.01 [-.08, .10]	.00 [-.11, .10]	**.66* [.55, .76]**	.01 [-.10, .12]	.01 [-.09, .12]	.04 [-.06, .14]	.00 [-.12, .13]	.00 [-.09, .09]
Item 41	-.01 [-.12, .10]	-.01 [-.13, .10]	-.02 [-.12, .07]	.00 [-.11, .11]	**.62* [.51, .72]**	.01 [-.10, .12]	.00 [-.10, .11]	.00 [-.10, .10]	.02 [-.10, .15]	.02 [-.08, .11]
Item 7	.01 [-.09, .12]	-.01 [-.13, .10]	-.01 [-.10, .08]	-.05 [-.15, .06]	.01 [-.08, .10]	**.52* [.31, .71]**	.00 [-.11, .12]	-.01 [-.11, .10]	.02 [-.10, .15]	-.03 [-.12, .06]
Item 29	-.01 [-.12, .09]	-.01 [-.12, .10]	-.01 [-.10, .08]	-.01 [-.11, .09]	.01 [-.07, .10]	**.63* [.49, .79]**	-.02 [-.13, .09]	.04 [-.05, .14]	-.03 [-.15, .09]	-.04 [-.13, .04]
Item 30	.01 [-.09, .12]	-.01 [-.13, .10]	.00 [-.09, .09]	-.03 [-.13, .08]	.02 [-.06, .11]	**.62* [.44, .81]**	.04 [-.07, .16]	.01 [-.09, .11]	.03 [-.09, .15]	.01 [-.08, .10]
Item 40	.01 [-.10, .11]	.00 [-.12, .11]	.02 [-.08, .11]	.00 [-.10, .11]	-.02 [-.11, .07]	**.46* [.26, .66]**	.01 [-.10, .12]	.02 [-.08, .12]	-.02 [-.15, .10]	.01 [-.09, .09]
Item 44	.02 [-.08, .13]	.06 [-.06, .17]	.00 [-.09, .09]	-.03 [-.13, .07]	-.03 [-.12, .06]	**.58* [.39, .77]**	.01 [-.09, .12]	-.02 [-.12, .08]	-.01 [-.13, .11]	-.04 [-.13, .04]
Item 5	.01 [-.10, .12]	.05 [-.07, .16]	-.01 [-.11, .08]	-.04 [-.14, .07]	-.02 [-.11, .08]	.03 [-.08, .15]	**.35* [.12, .57]**	.02 [-.09, .12]	-.02 [-.15, .11]	-.01 [-.10, .09]
Item 13	.01 [-.10, .11]	.00 [-.12, .11]	.06 [-.03, .16]	.06 [-.04, .17]	.03 [-.06, .11]	.00 [-.12, .11]	**.57* [.40, .77]**	-.01 [-.11, .09]	-.01 [-.13, .11]	.02 [-.06, .11]
Item 14	.00 [-.11, .10]	.00 [-.11, .12]	.00 [-.09, .09]	.04 [-.06, .15]	-.01 [-.09, .08]	.04 [-.07, .16]	**.65* [.44, .86]**	-.01 [-.11, .10]	.00 [-.13, .12]	.04 [-.05, .13]
Item 18	.02 [-.09, .12]	.06 [-.06, .17]	.01 [-.09, .10]	-.01 [-.12, .09]	.03 [-.06, .12]	.01 [-.11, .13]	**.34* [.11, .57]**	-.02 [-.12, .09]	-.03 [-.16, .10]	-.05 [-.14, .04]
Item 34	-.01 [-.12, .09]	.00 [-.11, .11]	-.01 [-.10, .09]	-.09 [-.20, .01]	.01 [-.08, .10]	.05 [-.07, .17]	**.47* [.25, .69]**	.03 [-.07, .13]	-.01 [-.14, .12]	-.04 [-.13, .05]
Item 23	-.02 [-.12, .09]	-.03 [-.15, .08]	-.01 [-.10, .09]	.00 [-.10, .11]	.07 [-.02, .16]	-.01 [-.12, .10]	.00 [-.11, .11]	**.68* [.52, .84]**	.02 [-.10, .14]	.03 [-.06, .12]
Item 28	.00 [-.10, .10]	.03 [-.08, .14]	-.01 [-.09, .09]	-.01 [-.11, .09]	-.04 [-.13, .05]	.03 [-.07, .15]	.01 [-.10, .12]	**.32* [.15, .50]**	.04 [-.09, .16]	-.04 [-.13, .05]
Item 31	-.02 [-.12, .09]	-.03 [-.14, .09]	.00 [-.09, .09]	-.05 [-.16, .05]	.02 [-.07, .11]	.01 [-.10, .12]	.00 [-.11, .10]	**.58* [.46, .73]**	.00 [-.12, .12]	-.05 [-.14, .04]
Item 45	.01 [-.10, .11]	-.04 [-.15, .08]	-.02 [-.11, .07]	.00 [-.10, .11]	.01 [-.08, .10]	.01 [-.10, .13]	.01 [-.10, .12]	**.67* [.51, .83]**	.05 [-.08, .17]	-.02 [-.11, .07]
Item 46	.02 [-.08, .13]	.03 [-.08, .15]	.01 [-.08, .11]	.03 [-.08, .14]	-.02 [-.11, .08]	.00 [-.11, .12]	.01 [-.09, .12]	**.04 [-.15, .23]**	.00 [-.12, .13]	.04 [-.06, .13]
Item 19	-.01 [-.11, .10]	.02 [-.10, .14]	-.01 [-.10, .08]	-.04 [-.15, .06]	.00 [-.09, .09]	-.04 [-.16, .07]	-.03 [-.15, .07]	-.03 [-.13, .07]	**.72* [.59, .88]**	.03 [-.06, .12]
Item 27	.01 [-.09, .11]	-.02 [-.13, .10]	.04 [-.05, .13]	-.05 [-.15, .06]	.02 [-.07, .11]	.05 [-.06, .17]	.05 [-.06, .16]	.06 [-.04, .17]	**.33* [.13, .52]**	-.02 [-.11, .07]
Item 33	-.01 [-.12, .09]	-.02 [-.13, .10]	.02 [-.07, .11]	.03 [-.08, .14]	.02 [-.07, .11]	.05 [-.05, .17]	.02 [-.08, .13]	.07 [-.03, .17]	**.49* [.29, .67]**	.10 [.00, .20]
Item 35	.00 [-.10, .10]	-.01 [-.12, .10]	.01 [-.08, .10]	.04 [-.07, .15]	.05 [-.04, .13]	.00 [-.11, .11]	.00 [-.10, .11]	.03 [-.07, .13]	**.30* [.10, .49]**	.30* [.21, .40]
Item 47	.01 [-.09, .12]	-.01 [-.12, .11]	-.01 [-.10, .09]	.04 [-.08, .15]	.03 [-.06, .12]	.00 [-.11, .11]	-.01 [-.12, .10]	.03 [-.08, .13]	**.53* [.33, .72]**	.02 [-.07, .11]
Item 3	-.03 [-.14, .07]	-.05 [-.17, .06]	-.01 [-.10, .09]	-.02 [-.12, .09]	-.05 [-.15, .04]	.00 [-.11, .11]	-.01 [-.11, .10]	.00 [-.10, .09]	.02 [-.10, .14]	**.74* [.68, .82]**
Item 4	.02 [-.09, .12]	-.01 [-.12, .11]	.00 [-.09, .09]	.05 [-.06, .16]	.00 [-.09, .09]	-.02 [-.13, .09]	.00 [-.11, .11]	-.01 [-.11, .09]	.01 [-.12, .13]	**.73* [.64, .82]**
Item 11	.02 [-.08, .13]	.05 [-.07, .16]	.01 [-.08, .10]	.12* [.02, .23]	.02 [-.07, .11]	-.01 [-.12, .10]	.01 [-.10, .11]	-.01 [-.11, .09]	.05 [-.08, .17]	**.62* [.53, .71]**
Item 32	.01 [-.09, .11]	.00 [-.11, .10]	.01 [-.07, .10]	.09 [-.01, .19]	.04 [-.05, .12]	.06 [-.04, .17]	.06 [-.03, .18]	.04 [-.05, .14]	.31* [.18, .45]	**.12* [.02, .22]**
Item 36	.01 [-.09, .12]	.02 [-.09, .14]	-.01 [-.10, .08]	-.06 [-.16, .05]	-.01 [-.11, .08]	.01 [-.10, .12]	.00 [-.10, .10]	-.01 [-.11, .09]	.00 [-.12, .12]	**.53* [.42, .63]**

Second-order factor loadings	Intrapersonal EC	Interpersonal EC
Identification-self	**.99* [.83, 1.20]**	-.03 [-.34, .22]
Comprehension-self	**.90* [.77, 1.07]**	-.10 [-.33, .11]
Expression-self	**.65* [.42, .87]**	.18 [-.10, .41]
Regulation-self	**.61* [.39, .79]**	-.03 [-.23, .17]
Utilization-self	**.20 [-.09, .47]**	.29* [.06, .48]
Identification-other	.06 [-.19, .29]	**.88* [.71, 1.05]**
Comprehension-other	.11 [-.16, .35]	**.89* [.71, 1.07]**

Expression-other	-.12 [-.39, .11]	**.90* [.74, 1.09]**								
Regulation-other	.00 [-.22, .21]	**.83* [.65, .98]**								
Utilization-other	.00 [-.21, .20]	**.34* [.07, .57]**								
Factor correlation	Interpersonal EC									
Intrapersonal EC	**.67* [.38, .86]**									
Residual correlation	Identification-self	Comprehension-self	Expression-self	Regulation-self	Utilization-self	Identification-other	Comprehension-other	Expression-other	Regulation-other	Utilization-other
Comprehension-self	.04 [-.09, .17]									
Expression-self	.01 [-.13, .14]	-.01 [-.14, .12]								
Regulation-self	.01 [-.12, .15]	.04 [-.09, .16]	.00 [-.13, .13]							
Utilization-self	.00 [-.13, .13]	-.05 [-.17, .07]	.03 [-.09, .15]	.01 [-.11, .12]						
Identification-other	**-.32* [-.43, -.19]**	-.01 [-.14, .12]	-.01 [-.14, .12]	-.03 [-.16, .10]	.00 [-.12, .13]					
Comprehension-other	-.03 [-.16, .11]	**-.21* [-.33, -.08]**	.02 [-.12, .15]	-.01 [-.14, .13]	.01 [-.12, .14]	.04 [-.09, .18]				
Expression-other	-.01 [-.14, .13]	-.03 [-.16, .10]	**.01 [-.12, .13]**	.00 [-.13, .12]	.04 [-.08, .16]	.02 [-.10, .15]	.01 [-.13, .14]			
Regulation-other	.00 [-.14, .14]	.00 [-.13, .14]	.01 [-.12, .14]	**.46* [.35, .56]**	.03 [-.09, .16]	-.01 [-.14, .13]	-.01 [-.15, .12]	.05 [-.08, .18]		
Utilization-other	.01 [-.13, .15]	-.02 [-.15, .11]	.00 [-.12, .13]	.08 [-.04, .20]	**.10 [-.02, .21]**	-.03 [-.16, .11]	.00 [-.13, .14]	-.01 [-.14, .12]	.10 [-.04, .22]	
	Item 1	Item 2	Item 3	Item 4	Item 5	Item 6	Item 7	Item 8	Item 9	Item 10
Item 2	.24* [.14, .33]									
Item 3	-.07 [-.18, .04]	-.07 [-.17, .04]								
Item 4	-.03 [-.14, .08]	-.05 [-.16, .05]	.12* [.01, .23]							
Item 5	.20* [.12, .29]	.14* [.06, .22]	-.03 [-.13, .06]	-.06 [-.15, .04]						
Item 6	.04 [-.05, .13]	-.04 [-.12, .04]	-.07 [-.16, .03]	.03 [-.06, .13]	.00 [-.07, .07]					
Item 7	-.04 [-.13, .05]	-.04 [-.12, .05]	-.03 [-.13, .06]	.02 [-.08, .12]	-.06 [-.13, .02]	.12* [.05, .19]				
Item 8	-.07 [-.16, .03]	-.06 [-.14, .04]	.00 [-.11, .10]	.07 [-.04, .17]	-.06 [-.14, .02]	.06 [-.03, .14]	.06 [-.03, .14]			
Item 9	.01 [-.08, .10]	.00 [-.08, .08]	-.02 [-.12, .07]	-.01 [-.11, .08]	.07 [.00, .15]	-.04 [-.11, .03]	-.06 [-.14, .01]	-.03 [-.12, .05]		
Item 10	-.02 [-.11, .07]	-.10* [-.19, -.01]	.01 [-.09, .11]	.02 [-.08, .12]	-.09* [-.17, -.02]	.07 [-.01, .15]	.09* [.01, .16]	.15* [.06, .23]	-.13* [-.21, -.05]	
Item 11	-.03 [-.13, .07]	-.01 [-.10, .09]	.09 [-.01, .20]	.08 [-.03, .19]	.01 [-.08, .10]	.04 [-.04, .13]	-.01 [-.10, .08]	.03 [-.07, .13]	-.03 [-.12, .06]	.01 [-.09, .10]
Item 12	-.02 [-.13, .09]	-.06 [-.16, .05]	.03 [-.08, .15]	.05 [-.07, .16]	-.09 [-.18, .02]	.06 [-.04, .16]	-.01 [-.11, .09]	.06 [-.05, .17]	-.09 [-.18, .01]	.03 [-.07, .13]
Item 13	-.06 [-.15, .04]	-.05 [-.13, .04]	.03 [-.08, .13]	.06 [-.04, .16]	-.09* [-.17, -.02]	.09* [.01, .16]	.08* [.00, .16]	.18* [.10, .27]	-.03 [-.11, .05]	.08 [.00, .17]
Item 14	-.08 [-.17, .02]	-.01 [-.10, .08]	.02 [-.08, .13]	.06 [-.05, .16]	-.05 [-.14, .04]	.02 [-.06, .10]	.03 [-.05, .12]	.14* [.05, .23]	-.02 [-.11, .06]	.07 [-.02, .15]
Item 15	-.04 [-.14, .05]	-.03 [-.12, .06]	.00 [-.10, .10]	.03 [-.07, .14]	-.06 [-.14, .02]	-.03 [-.11, .06]	.02 [-.06, .11]	.04 [-.06, .13]	-.02 [-.10, .06]	.01 [-.08, .10]
Item 16	-.04 [-.14, .06]	-.06 [-.15, .03]	.01 [-.10, .11]	-.01 [-.12, .09]	-.09* [-.17, -.01]	.00 [-.08, .09]	.11* [.02, .19]	.09 [-.01, .18]	-.08* [-.17, .00]	.23* [.14, .31]
Item 17	-.03 [-.12, .06]	.05 [-.04, .13]	.02 [-.08, .12]	.02 [-.08, .11]	-.11* [-.18, -.03]	.02 [-.06, .09]	-.03 [-.11, .05]	.05 [-.05, .14]	-.07 [-.15, .00]	.01 [-.07, .09]
Item 18	.11* [.02, .20]	.16* [.08, .24]	-.07 [-.17, .03]	-.07 [-.17, .03]	.21* [.14, .28]	-.01 [-.08, .07]	-.04 [-.11, .04]	-.12* [-.19, -.03]	.11* [.04, .18]	-.11* [-.19, -.04]
Item 19	-.06 [-.16, .04]	.00 [-.09, .09]	.02 [-.09, .12]	.04 [-.06, .15]	-.08* [-.16, .00]	.05 [-.03, .13]	.10* [.02, .19]	-.01 [-.11, .08]	-.07 [-.15, .01]	.01 [-.08, .09]
Item 20	.09* [.00, .18]	.08 [-.01, .16]	.00 [-.10, .09]	.01 [-.09, .10]	.11* [.04, .18]	-.02 [-.09, .06]	-.02 [-.10, .05]	-.06 [-.14, .03]	.11* [.03, .18]	-.03 [-.11, .05]
Item 21	-.07 [-.17, .03]	-.02 [-.12, .07]	-.02 [-.12, .09]	-.03 [-.13, .08]	-.03 [-.12, .06]	.04 [-.05, .13]	.02 [-.07, .11]	.01 [-.09, .11]	.02 [-.07, .12]	-.01 [-.10, .09]

Item 22	.01 [-.08, .10]	-.04 [-.13, .04]	-.07 [-.17, .03]	.04 [-.05, .14]	-.07 [-.15, .00]	.12* [.05, .20]	.06 [-.01, .14]	-.02 [-.11, .06]	-.03 [-.11, .05]	.05 [-.04, .13]
Item 23	-.03 [-.13, .08]	-.02 [-.12, .08]	.05 [-.05, .16]	.02 [-.09, .12]	.04 [-.05, .12]	.01 [-.07, .10]	-.02 [-.10, .07]	.05 [-.05, .14]	.00 [-.09, .08]	-.01 [-.11, .08]
Item 24	-.06 [-.16, .05]	-.10 [-.19, .00]	.01 [-.10, .12]	.07 [-.04, .18]	-.08 [-.17, .01]	.04 [-.06, .12]	.03 [-.06, .12]	.14* [.04, .24]	-.02 [-.11, .07]	.09 [-.01, .18]
Item 25	.02 [-.08, .13]	.03 [-.06, .13]	.01 [-.10, .12]	-.06 [-.16, .05]	.07 [-.02, .15]	-.07 [-.15, .01]	-.06 [-.15, .02]	.07 [-.03, .17]	.08 [.00, .17]	-.08 [-.17, .01]
Item 26	.02 [-.08, .13]	.05 [-.04, .15]	.00 [-.11, .10]	-.05 [-.15, .05]	.06 [-.01, .14]	-.05 [-.13, .03]	-.07 [-.14, .01]	-.07 [-.16, .02]	.01 [-.07, .08]	-.08 [-.16, .01]
Item 27	.01 [-.08, .10]	.01 [-.07, .09]	-.05 [-.15, .04]	-.06 [-.15, .04]	.04 [-.03, .12]	-.04 [-.11, .03]	.03 [-.05, .10]	.01 [-.07, .09]	.02 [-.06, .10]	-.03 [-.11, .05]
Item 28	.04 [-.05, .13]	.09* [.01, .17]	-.07 [-.17, .03]	-.08 [-.17, .02]	.10* [.02, .17]	-.09* [-.16, -.02]	.00 [-.07, .08]	-.09* [-.17, .00]	.03 [-.05, .10]	-.06 [-.14, .02]
Item 29	.08 [-.02, .18]	.09 [.00, .18]	-.06 [-.17, .05]	-.08 [-.18, .03]	.16* [.07, .23]	-.06 [-.14, .02]	-.02 [-.10, .07]	-.10* [-.19, -.01]	.16* [.08, .24]	-.10* [-.18, -.01]
Item 30	-.04 [-.13, .06]	-.07 [-.16, .02]	.01 [-.09, .12]	.00 [-.10, .11]	.02 [-.06, .10]	.07 [-.01, .15]	.08 [-.01, .16]	.01 [-.08, .10]	-.07 [-.15, .02]	.06 [-.03, .14]
Item 31	.07 [-.02, .17]	.02 [-.07, .10]	-.08 [-.17, .03]	-.02 [-.12, .08]	.06 [-.02, .14]	-.05 [-.12, .03]	.01 [-.07, .09]	-.09 [-.17, .00]	.08* [.00, .16]	-.06 [-.14, .03]
Item 32	-.08 [-.17, .02]	-.06 [-.15, .03]	.01 [-.09, .12]	.06 [-.04, .17]	-.09* [-.16, -.01]	.00 [-.07, .08]	.03 [-.05, .11]	.02 [-.07, .11]	-.03 [-.11, .05]	.00 [-.09, .08]
Item 33	-.03 [-.14, .07]	-.05 [-.15, .04]	.01 [-.10, .13]	.08 [-.04, .19]	-.03 [-.11, .05]	.00 [-.08, .09]	.02 [-.07, .11]	.02 [-.08, .12]	-.01 [-.09, .08]	.01 [-.09, .10]
Item 34	.05 [-.05, .14]	.08 [.00, .17]	-.06 [-.16, .04]	-.06 [-.16, .04]	.10* [.02, .18]	-.05 [-.13, .02]	.00 [-.08, .08]	-.06 [-.14, .03]	.08 [.00, .15]	-.01 [-.10, .07]
Item 35	-.03 [-.12, .07]	-.06 [-.15, .03]	.14* [.04, .24]	.09 [-.01, .20]	-.09* [-.17, -.01]	-.03 [-.11, .05]	-.07 [-.15, .01]	.04 [-.05, .13]	-.03 [-.11, .05]	.00 [-.08, .09]
Item 36	.00 [-.09, .09]	-.08 [-.16, .01]	.15* [.04, .25]	.03 [-.08, .13]	-.07 [-.14, .01]	.04 [-.04, .12]	-.02 [-.10, .07]	.06 [-.03, .14]	-.01 [-.09, .07]	.02 [-.07, .10]
Item 37	.13* [.03, .22]	.15* [.06, .24]	-.02 [-.12, .07]	-.04 [-.13, .06]	.11* [.03, .19]	-.09* [-.16, -.02]	-.04 [-.12, .04]	-.02 [-.10, .07]	.01 [-.07, .08]	-.04 [-.12, .05]
Item 38	.03 [-.06, .13]	-.01 [-.10, .08]	.02 [-.08, .12]	-.08 [-.18, .02]	.00 [-.08, .07]	-.07 [-.14, .01]	-.04 [-.11, .04]	.01 [-.08, .10]	.11* [.04, .19]	-.05 [-.13, .03]
Item 39	-.03 [-.13, .08]	.04 [-.06, .14]	.02 [-.09, .13]	.05 [-.06, .16]	-.01 [-.10, .07]	-.02 [-.11, .07]	-.04 [-.13, .05]	-.01 [-.11, .09]	-.05 [-.13, .04]	.01 [-.08, .10]
Item 40	.10* [.01, .19]	.09* [.00, .17]	.02 [-.08, .12]	-.08 [-.18, .02]	.15* [.07, .22]	-.09* [-.16, -.01]	-.05 [-.13, .03]	-.05 [-.13, .04]	.07 [.00, .15]	-.10* [-.18, -.02]
Item 41	-.07 [-.17, .03]	-.04 [-.13, .06]	.03 [-.08, .14]	.04 [-.06, .15]	-.09* [-.17, -.01]	.09* [.01, .18]	.05 [-.04, .13]	.01 [-.08, .11]	.01 [-.08, .10]	.05 [-.04, .14]
Item 42	.10* [.01, .19]	.11* [.03, .19]	-.06 [-.16, .04]	-.02 [-.12, .07]	.12* [.05, .19]	.00 [-.07, .07]	-.06 [-.13, .01]	-.11* [-.20, -.02]	.12* [.05, .19]	-.05 [-.12, .03]
Item 43	.09 [-.02, .20]	.08 [-.02, .19]	-.01 [-.12, .10]	-.09 [-.20, .02]	.04 [-.05, .13]	-.06 [-.14, .03]	-.06 [-.14, .03]	-.12* [-.21, -.02]	.04 [-.04, .13]	-.02 [-.11, .08]
Item 44	.06 [-.04, .16]	.10* [.01, .19]	-.05 [-.16, .05]	-.11* [-.21, .00]	.19* [.11, .26]	-.08 [-.15, .00]	-.03 [-.12, .05]	-.11* [-.20, -.02]	.13* [.05, .21]	-.10* [-.18, -.01]
Item 45	-.03 [-.13, .07]	-.05 [-.14, .05]	.04 [-.07, .14]	-.01 [-.12, .09]	-.04 [-.12, .05]	-.01 [-.09, .08]	-.03 [-.11, .06]	-.04 [-.13, .06]	-.01 [-.10, .07]	-.01 [-.10, .09]
Item 46	.07 [-.01, .15]	.03 [-.05, .11]	.01 [-.09, .10]	.00 [-.10, .09]	.07* [.00, .14]	-.05 [-.12, .02]	-.09* [-.16, -.02]	.01 [-.07, .09]	.04 [-.03, .11]	-.08* [-.15, .00]
Item 47	-.05 [-.14, .05]	-.05 [-.14, .04]	.06 [-.04, .17]	.00 [-.10, .11]	-.10* [-.17, -.02]	.06 [-.02, .14]	.04 [-.04, .12]	-.03 [-.12, .06]	-.08 [-.15, .00]	.02 [-.07, .10]
Item 48	-.07 [-.16, .03]	-.09* [-.17, .00]	-.02 [-.12, .08]	.06 [-.04, .16]	-.10* [-.17, -.02]	.06 [-.03, .14]	.00 [-.08, .09]	.05 [-.05, .14]	-.03 [-.11, .05]	.15* [.06, .23]
Item 49	.06 [-.04, .16]	.05 [-.04, .15]	.00 [-.10, .10]	-.03 [-.13, .07]	.09* [.01, .17]	-.06 [-.14, .02]	-.03 [-.11, .05]	-.02 [-.11, .07]	.07 [-.01, .15]	-.02 [-.10, .07]
Item 50	.02 [-.08, .11]	-.03 [-.11, .05]	-.01 [-.11, .09]	.06 [-.04, .15]	-.08* [-.16, -.01]	.07 [.00, .14]	-.03 [-.11, .04]	-.03 [-.11, .06]	-.06 [-.14, .01]	.07 [-.01, .15]
	Item 11	Item 12	Item 13	Item 14	Item 15	Item 16	Item 17	Item 18	Item 19	Item 20
Item 12	.08 [-.03, .19]									
Item 13	.01 [-.08, .11]	.08 [-.02, .19]								
Item 14	.05 [-.04, .15]	.04 [-.07, .15]	.21* [.12, .29]							
Item 15	.09 [-.01, .18]	.12* [.02, .23]	.06 [-.03, .14]	.09* [.00, .18]						
Item 16	.03 [-.07, .12]	.03 [-.08, .14]	.03 [-.06, .12]	.07 [-.02, .17]	.08 [-.01, .17]					
Item 17	.03 [-.06, .12]	.05 [-.06, .15]	.09* [.00, .17]	.09* [.00, .17]	.01 [-.07, .10]	.09* [.00, .17]				
Item 18	-.03 [-.12, .06]	-.10 [-.19, .00]	-.04 [-.12, .04]	-.08 [-.17, .01]	-.12* [-.20, -.04]	-.08 [-.16, .01]	-.05 [-.12, .03]			

Item 19	.04 [-.06, .13]	.03 [-.07, .14]	.06 [-.03, .15]	.06 [-.03, .15]	.02 [-.07, .11]	.01 [-.08, .11]	.09* [.01, .18]	-.12* [-.20, -.04]		
Item 20	-.06 [-.14, .03]	-.02 [-.12, .08]	-.08 [-.15, .00]	-.09* [-.17, .00]	-.09* [-.17, -.01]	-.05 [-.13, .04]	-.04 [-.12, .04]	.12* [.04, .19]	-.09* [-.17, .00]	
Item 21	.05 [-.06, .15]	.03 [-.09, .14]	.01 [-.09, .10]	.01 [-.09, .11]	-.01 [-.10, .09]	.05 [-.05, .15]	.10* [.00, .18]	-.05 [-.14, .04]	.07 [-.03, .17]	-.12* [-.21, -.03]
Item 22	-.02 [-.11, .07]	.05 [-.05, .15]	.07 [-.01, .15]	.01 [-.08, .09]	-.01 [-.09, .08]	.04 [-.04, .13]	.07 [-.01, .15]	-.01 [-.09, .06]	.06 [-.03, .14]	-.07 [-.14, .01]
Item 23	.04 [-.06, .14]	.01 [-.10, .12]	.03 [-.06, .13]	.05 [-.04, .15]	.02 [-.07, .12]	-.02 [-.12, .07]	-.01 [-.10, .08]	-.07 [-.16, .01]	.02 [-.08, .12]	-.02 [-.11, .07]
Item 24	.04 [-.06, .15]	.04 [-.07, .16]	.10* [.01, .20]	.06 [-.04, .16]	.05 [-.05, .14]	.05 [-.06, .15]	.00 [-.09, .10]	-.10* [-.18, -.01]	.01 [-.09, .11]	-.08 [-.17, .01]
Item 25	-.02 [-.12, .08]	-.07 [-.18, .04]	.01 [-.08, .10]	-.08 [-.17, .01]	-.03 [-.12, .06]	-.03 [-.12, .07]	-.03 [-.13, .06]	.10* [.02, .18]	-.09 [-.18, .01]	.02 [-.07, .10]
Item 26	-.02 [-.11, .08]	-.05 [-.15, .06]	-.08* [-.17, .00]	-.06 [-.14, .03]	-.05 [-.13, .04]	-.02 [-.11, .07]	-.09* [-.16, -.01]	.13* [.05, .20]	-.05 [-.13, .04]	.13* [.05, .21]
Item 27	-.04 [-.13, .05]	-.06 [-.16, .04]	-.03 [-.10, .05]	-.07 [-.15, .01]	-.04 [-.12, .05]	-.07 [-.15, .01]	-.08* [-.16, -.01]	.10* [.03, .17]	-.06 [-.14, .03]	.06 [-.02, .13]
Item 28	-.05 [-.14, .04]	-.08 [-.17, .03]	-.09* [-.17, -.01]	-.12* [-.20, -.04]	-.08 [-.16, .00]	-.05 [-.13, .04]	-.01 [-.09, .06]	.12* [.05, .19]	-.03 [-.11, .06]	.08* [.00, .15]
Item 29	-.09 [-.19, .01]	-.05 [-.16, .06]	-.10* [-.19, -.02]	-.09* [-.18, .00]	-.06 [-.15, .03]	-.08 [-.17, .02]	-.10* [-.18, -.02]	.15* [.07, .23]	-.10* [-.19, -.01]	.10* [.02, .18]
Item 30	-.01 [-.10, .09]	.00 [-.11, .10]	.06 [-.03, .15]	.15* [.05, .23]	-.03 [-.12, .06]	.04 [-.05, .13]	.00 [-.08, .09]	-.05 [-.13, .04]	.06 [-.03, .15]	-.11* [-.19, -.03]
Item 31	-.07 [-.16, .02]	-.06 [-.17, .04]	-.05 [-.13, .04]	-.10* [-.19, -.02]	-.04 [-.13, .05]	-.10* [-.18, -.01]	.00 [-.08, .09]	.08* [.00, .16]	-.06 [-.14, .03]	.06 [-.02, .14]
Item 32	.02 [-.08, .11]	.00 [-.10, .11]	.02 [-.07, .10]	.02 [-.07, .11]	.05 [-.04, .13]	-.04 [-.13, .05]	.03 [-.05, .11]	-.08* [-.15, -.01]	.15* [.06, .23]	-.06 [-.14, .02]
Item 33	.03 [-.08, .14]	.03 [-.09, .14]	-.02 [-.12, .07]	.00 [-.10, .10]	.07 [-.03, .16]	-.04 [-.15, .06]	.06 [-.03, .14]	-.08* [-.16, .00]	.06 [-.04, .16]	-.04 [-.12, .05]
Item 34	-.02 [-.11, .07]	-.10 [-.19, .01]	-.06 [-.14, .02]	-.07 [-.16, .02]	-.08 [-.16, .01]	-.04 [-.12, .05]	-.10* [-.17, -.02]	.12* [.04, .19]	-.08 [-.16, .00]	.08* [.00, .15]
Item 35	.15* [.06, .24]	.04 [-.06, .15]	-.01 [-.09, .08]	.07 [-.02, .16]	.07 [-.02, .15]	-.01 [-.11, .08]	.00 [-.08, .09]	-.06 [-.13, .02]	-.01 [-.10, .09]	-.02 [-.09, .06]
Item 36	.05 [-.04, .15]	.02 [-.08, .13]	.00 [-.08, .09]	.06 [-.03, .14]	.02 [-.07, .11]	.08 [-.01, .16]	-.04 [-.12, .04]	-.05 [-.12, .03]	.02 [-.07, .11]	.00 [-.08, .08]
Item 37	-.02 [-.11, .07]	-.03 [-.13, .08]	-.07 [-.15, .01]	-.07 [-.15, .01]	-.04 [-.13, .04]	-.02 [-.11, .07]	.02 [-.06, .09]	.09* [.02, .17]	-.08 [-.16, .01]	.13* [.05, .20]
Item 38	-.03 [-.12, .07]	-.07 [-.17, .04]	-.04 [-.12, .05]	-.11* [-.19, -.02]	.01 [-.08, .09]	-.06 [-.15, .03]	-.05 [-.13, .04]	.06 [-.02, .13]	-.07 [-.15, .02]	.03 [-.05, .11]
Item 39	.03 [-.08, .13]	.12* [.01, .23]	.02 [-.07, .11]	.05 [-.05, .15]	.04 [-.06, .13]	-.02 [-.12, .08]	.17* [.08, .25]	.01 [-.07, .10]	.06 [-.04, .15]	.00 [-.09, .09]
Item 40	-.03 [-.12, .07]	-.04 [-.15, .06]	-.06 [-.14, .02]	-.02 [-.10, .07]	.01 [-.08, .09]	-.09* [-.17, .00]	-.07 [-.14, .01]	.17* [.09, .24]	-.12* [-.20, -.03]	.14* [.07, .22]
Item 41	.01 [-.09, .11]	.02 [-.09, .13]	.04 [-.06, .13]	.06 [-.03, .16]	.00 [-.10, .09]	.06 [-.04, .15]	.01 [-.08, .10]	-.03 [-.11, .06]	.08 [-.02, .17]	-.09* [-.17, -.01]
Item 42	-.05 [-.14, .04]	-.03 [-.13, .07]	-.06 [-.13, .02]	-.09* [-.17, -.01]	-.06 [-.14, .02]	-.11* [-.18, -.02]	.04 [-.04, .11]	.13* [.06, .20]	.01 [-.08, .09]	.06 [-.01, .13]
Item 43	-.01 [-.11, .09]	-.02 [-.14, .09]	-.14* [-.22, -.04]	-.13* [-.23, -.04]	-.03 [-.12, .06]	-.01 [-.10, .10]	-.09* [-.17, .00]	.10* [.01, .18]	-.03 [-.13, .06]	.11* [.02, .20]
Item 44	-.04 [-.13, .06]	-.10 [-.20, .01]	-.09* [-.18, -.01]	-.07 [-.16, .02]	-.04 [-.12, .05]	-.09 [-.18, .00]	-.09* [-.17, -.01]	.20* [.12, .27]	-.11* [-.19, -.02]	.14* [.05, .21]
Item 45	-.01 [-.11, .09]	.03 [-.08, .13]	-.01 [-.10, .09]	.04 [-.06, .13]	-.02 [-.11, .07]	.07 [-.03, .17]	.02 [-.07, .11]	-.03 [-.11, .06]	.05 [-.04, .15]	-.04 [-.13, .05]
Item 46	-.05 [-.14, .03]	-.03 [-.12, .07]	.01 [-.07, .08]	-.01 [-.09, .06]	-.02 [-.10, .06]	-.07 [-.15, .01]	-.06 [-.13, .01]	.04 [-.03, .10]	-.06 [-.13, .02]	.08* [.01, .15]
Item 47	-.01 [-.11, .08]	.03 [-.07, .14]	-.02 [-.10, .07]	-.04 [-.13, .05]	.00 [-.09, .09]	.04 [-.05, .13]	.04 [-.04, .12]	-.04 [-.12, .03]	.14* [.05, .23]	-.06 [-.14, .02]
Item 48	.00 [-.09, .10]	.00 [-.11, .11]	.02 [-.07, .11]	.05 [-.04, .15]	.02 [-.07, .10]	.17* [.07, .26]	.05 [-.04, .13]	-.11* [-.19, -.03]	.04 [-.05, .13]	-.04 [-.12, .05]
Item 49	-.08 [-.17, .02]	-.03 [-.13, .08]	-.10* [-.18, -.02]	-.10* [-.19, -.01]	-.06 [-.15, .03]	-.06 [-.15, .04]	-.07 [-.15, .01]	.09* [.01, .17]	-.05 [-.14, .04]	.21* [.13, .29]
Item 50	-.01 [-.11, .08]	.04 [-.07, .15]	.04 [-.04, .12]	.05 [-.04, .13]	-.01 [-.10, .08]	.03 [-.05, .12]	.13* [.05, .20]	.00 [-.07, .08]	.09* [.00, .17]	-.04 [-.12, .03]
	Item 21	Item 22	Item 23	Item 24	Item 25	Item 26	Item 27	Item 28	Item 29	Item 30
Item 22	.12* [.02, .21]									
Item 23	.00 [-.10, .10]	.04 [-.05, .13]								
Item 24	.04 [-.06, .14]	.03 [-.07, .13]	.14* [.04, .24]							
Item 25	-.01 [-.11, .09]	-.09* [-.17, .00]	-.06 [-.16, .04]	-.09 [-.18, .01]						

Item 26	-.05 [-.15, .04]	-.11* [-.18, -.03]	-.08 [-.18, .01]	-.09 [-.18, .01]	.17* [.08, .26]					
Item 27	-.04 [-.13, .05]	.00 [-.08, .07]	-.06 [-.15, .02]	-.06 [-.15, .03]	.10* [.01, .18]	.07 [-.01, .15]				
Item 28	-.04 [-.13, .05]	-.03 [-.11, .05]	-.07 [-.16, .03]	-.12* [-.21, -.03]	.07 [-.01, .16]	.11* [.03, .18]	.27* [.19, .33]			
Item 29	-.05 [-.14, .05]	-.02 [-.10, .06]	-.07 [-.17, .02]	-.15* [-.24, -.06]	.11* [.02, .20]	.10* [.01, .19]	.20* [.12, .28]	.28* [.21, .36]		
Item 30	.05 [-.04, .15]	.06 [-.03, .14]	.03 [-.07, .12]	.05 [-.05, .15]	-.04 [-.13, .05]	-.02 [-.11, .07]	-.05 [-.13, .03]	-.07 [-.15, .02]	-.06 [-.15, .03]	
Item 31	-.02 [-.11, .08]	-.03 [-.11, .05]	-.02 [-.11, .07]	-.07 [-.16, .03]	.07 [-.02, .16]	.00 [-.09, .08]	.20* [.12, .28]	.23* [.15, .31]	.25* [.16, .33]	-.07 [-.16, .02]
Item 32	.06 [-.04, .15]	.05 [-.03, .13]	.06 [-.04, .15]	.03 [-.07, .13]	-.05 [-.14, .04]	-.05 [-.13, .04]	.04 [-.04, .12]	-.03 [-.10, .05]	-.12* [-.20, -.03]	.08 [.00, .17]
Item 33	.06 [-.04, .16]	.03 [-.06, .11]	.11* [.01, .21]	.03 [-.08, .13]	-.03 [-.13, .07]	-.13* [-.22, -.04]	.02 [-.07, .11]	-.04 [-.13, .04]	-.07 [-.16, .02]	.05 [-.05, .15]
Item 34	-.07 [-.15, .03]	.02 [-.05, .10]	-.02 [-.11, .07]	-.06 [-.15, .04]	.08 [-.01, .17]	.12* [.04, .19]	.15* [.07, .22]	.13* [.05, .20]	.19* [.10, .27]	.02 [-.07, .10]
Item 35	.01 [-.08, .11]	-.01 [-.09, .07]	.11* [.02, .20]	.10 [.00, .19]	-.04 [-.13, .05]	-.09* [-.17, .00]	-.08 [-.15, .00]	-.15* [-.22, -.07]	-.18* [-.26, -.09]	.05 [-.04, .14]
Item 36	-.01 [-.10, .09]	-.02 [-.11, .06]	.00 [-.09, .10]	.03 [-.07, .13]	-.04 [-.13, .05]	-.03 [-.11, .06]	-.08 [-.16, .00]	-.07 [-.15, .01]	-.08 [-.16, .01]	.06 [-.03, .15]
Item 37	.03 [-.06, .12]	-.05 [-.12, .03]	-.08 [-.17, .01]	-.12* [-.21, -.03]	.10* [.02, .19]	.13* [.05, .21]	.11* [.03, .19]	.09* [.01, .16]	.07 [-.01, .16]	-.07 [-.15, .02]
Item 38	.02 [-.08, .11]	-.06 [-.14, .02]	.01 [-.08, .10]	-.02 [-.12, .07]	.23* [.15, .31]	.04 [-.05, .12]	.16* [.09, .24]	.07 [.00, .15]	.12* [.04, .20]	-.05 [-.14, .04]
Item 39	-.01 [-.11, .09]	.00 [-.09, .09]	.05 [-.05, .15]	.06 [-.04, .16]	-.08 [-.17, .02]	.00 [-.09, .09]	-.09* [-.18, .00]	-.07 [-.15, .02]	-.12* [-.22, -.03]	.04 [-.06, .13]
Item 40	-.09 [-.18, .00]	-.10* [-.17, -.02]	-.01 [-.10, .08]	-.06 [-.15, .03]	.11* [.02, .20]	.14* [.06, .22]	.06 [-.02, .14]	.13* [.05, .20]	.15* [.06, .24]	-.08 [-.17, .01]
Item 41	.05 [-.05, .15]	.02 [-.07, .11]	.02 [-.08, .12]	.17* [.06, .27]	-.08 [-.17, .02]	-.07 [-.16, .02]	-.05 [-.14, .03]	-.07 [-.16, .01]	-.07 [-.16, .02]	.04 [-.05, .14]
Item 42	.05 [-.04, .14]	.00 [-.08, .07]	-.08 [-.16, .01]	-.11* [-.20, -.03]	.07 [-.02, .16]	.06 [-.01, .14]	.07 [.00, .15]	.08* [.01, .15]	.18* [.11, .26]	-.01 [-.09, .07]
Item 43	-.03 [-.14, .07]	-.01 [-.10, .08]	-.06 [-.16, .05]	-.12* [-.22, -.02]	.09 [-.02, .19]	.19* [.09, .29]	.08 [-.01, .17]	.14* [.06, .23]	.21* [.11, .30]	-.08 [-.18, .01]
Item 44	-.08 [-.17, .02]	-.02 [-.10, .06]	-.04 [-.13, .06]	-.14* [-.23, -.04]	.11* [.02, .20]	.12* [.04, .21]	.07 [-.01, .15]	.08* [.00, .16]	.25* [.16, .34]	-.06 [-.15, .04]
Item 45	.03 [-.07, .13]	.03 [-.06, .11]	.12* [.02, .22]	.02 [-.08, .13]	-.06 [-.15, .04]	-.02 [-.11, .08]	-.05 [-.13, .04]	-.05 [-.14, .05]	.01 [-.09, .10]	.05 [-.04, .15]
Item 46	-.05 [-.13, .04]	-.07* [-.14, .00]	.00 [-.09, .09]	.00 [-.09, .08]	.07 [-.01, .14]	.12* [.04, .19]	.10* [.03, .17]	.08* [.01, .15]	.09* [.02, .17]	-.08 [-.16, .00]
Item 47	.08 [-.01, .18]	.01 [-.08, .09]	.02 [-.07, .12]	.04 [-.06, .14]	-.01 [-.10, .08]	-.04 [-.12, .05]	-.05 [-.13, .03]	-.06 [-.13, .02]	-.07 [-.16, .01]	.07 [-.02, .16]
Item 48	.04 [-.06, .13]	.03 [-.05, .12]	-.02 [-.11, .08]	.07 [-.03, .16]	-.07 [-.16, .02]	.03 [-.06, .11]	-.07 [-.15, .01]	-.07 [-.15, .01]	-.13* [-.22, -.04]	.07 [-.02, .16]
Item 49	-.07 [-.17, .02]	-.06 [-.14, .02]	-.04 [-.14, .05]	-.07 [-.16, .03]	.12* [.02, .21]	.16* [.07, .25]	.06 [-.02, .14]	.10* [.02, .18]	.14* [.05, .23]	-.06 [-.15, .03]
Item 50	.05 [-.05, .14]	.19* [.12, .27]	.01 [-.08, .10]	.04 [-.06, .13]	-.09* [-.17, .00]	-.05 [-.13, .03]	-.10* [-.17, -.03]	-.05 [-.12, .03]	-.10* [-.18, -.01]	.06 [-.03, .14]
	Item 31	Item 32	Item 33	Item 34	Item 35	Item 36	Item 37	Item 38	Item 39	Item 40
Item 32	-.04 [-.12, .05]									
Item 33	-.01 [-.10, .08]	.47* [.40, .54]								
Item 34	.14* [.05, .22]	-.10* [-.17, -.02]	-.12* [-.20, -.03]							
Item 35	-.10* [-.18, -.02]	.12* [.04, .21]	.19* [.10, .28]	-.10* [-.18, -.02]						
Item 36	-.10* [-.18, -.01]	.02 [-.06, .11]	.01 [-.09, .10]	-.08 [-.16, .00]	.15* [.07, .24]					
Item 37	.09* [.01, .17]	.00 [-.08, .08]	-.06 [-.15, .03]	.10* [.02, .17]	-.07 [-.15, .01]	-.08 [-.16, .00]				
Item 38	.12* [.04, .20]	.01 [-.07, .09]	-.01 [-.10, .07]	.10* [.02, .18]	-.02 [-.10, .07]	-.08 [-.16, .01]	.11* [.03, .19]			
Item 39	-.11* [-.20, -.02]	.04 [-.05, .14]	.04 [-.07, .14]	-.10* [-.19, -.01]	.05 [-.05, .14]	-.05 [-.14, .04]	-.02 [-.11, .07]	-.14* [-.22, -.05]		
Item 40	.10* [.01, .18]	-.13* [-.21, -.06]	-.12* [-.21, -.04]	.15* [.07, .22]	-.03 [-.11, .05]	-.04 [-.12, .04]	.10* [.02, .18]	.10* [.02, .18]	-.10* [-.19, -.01]	
Item 41	-.06 [-.15, .03]	.03 [-.06, .12]	.02 [-.08, .12]	-.08 [-.17, .01]	.07 [-.03, .16]	.06 [-.03, .15]	-.12* [-.21, -.04]	-.07 [-.16, .02]	.08 [-.02, .18]	-.10* [-.19, -.02]
Item 42	.10* [.03, .18]	-.04 [-.11, .04]	-.05 [-.13, .03]	.10* [.03, .18]	-.10* [-.17, -.02]	-.18* [-.25, -.10]	.14* [.06, .21]	.15* [.07, .22]	-.04 [-.12, .05]	.11* [.04, .18]

Item 43	.10* [.01, .19]	-.05 [-.14, .04]	-.08 [-.19, .02]	.06 [-.03, .15]	-.09 [-.18, .00]	-.04 [-.13, .05]	.14* [.05, .23]	.11* [.02, .20]	-.10 [-.19, .00]	.09 [.00, .18]
Item 44	.11* [.02, .20]	-.11* [-.19, -.02]	-.08 [-.17, .01]	.20* [.12, .28]	-.06 [-.14, .02]	-.04 [-.12, .05]	.10* [.01, .18]	.09* [.00, .17]	-.11* [-.21, -.02]	.27* [.18, .34]
Item 45	-.03 [-.12, .06]	-.03 [-.12, .06]	.02 [-.08, .12]	-.02 [-.11, .07]	.08 [-.01, .17]	.04 [-.05, .13]	-.06 [-.15, .03]	-.04 [-.13, .05]	.01 [-.09, .11]	-.01 [-.10, .08]
Item 46	.08* [.00, .16]	.00 [-.07, .07]	-.04 [-.12, .04]	.09* [.02, .16]	-.05 [-.12, .02]	.00 [-.08, .07]	.07 [.00, .14]	.08* [.01, .15]	-.03 [-.11, .06]	.13* [.06, .20]
Item 47	-.01 [-.09, .07]	.13* [.04, .21]	.10* [.00, .20]	-.07 [-.15, .01]	.05 [-.04, .14]	.08 [-.01, .16]	-.09* [-.17, .00]	-.12* [-.20, -.04]	.13* [.04, .22]	-.12* [-.20, -.05]
Item 48	-.03 [-.12, .05]	.05 [-.04, .14]	-.01 [-.11, .08]	-.07 [-.15, .02]	.06 [-.03, .15]	.06 [-.03, .14]	-.04 [-.12, .04]	-.07 [-.15, .01]	.09 [-.01, .18]	-.09* [-.17, -.01]
Item 49	.07 [-.02, .16]	-.06 [-.14, .02]	-.09 [-.18, .01]	.09* [.01, .17]	-.10* [-.18, -.01]	-.02 [-.10, .07]	.15* [.06, .23]	.09 [.00, .17]	-.08 [-.17, .02]	.12* [.04, .20]
Item 50	-.12* [-.19, -.04]	.05 [-.03, .13]	-.01 [-.09, .08]	-.06 [-.13, .02]	.12* [.04, .20]	.05 [-.03, .13]	-.02 [-.10, .06]	-.09* [-.16, -.01]	.14* [.05, .23]	-.09* [-.17, -.02]
	Item 41	Item 42	Item 43	Item 44	Item 45	Item 46	Item 47	Item 48	Item 49	Item 50
Item 42	-.10* [-.18, -.02]									
Item 43	-.08 [-.18, .02]	.10* [.02, .19]								
Item 44	-.12* [-.20, -.03]	.15* [.07, .22]	.26* [.17, .35]							
Item 45	.05 [-.05, .15]	-.03 [-.12, .05]	-.08 [-.18, .02]	-.03 [-.13, .06]						
Item 46	-.13* [-.21, -.05]	.09* [.03, .16]	.07 [-.02, .15]	.11* [.03, .18]	-.09 [-.17, .00]					
Item 47	.10* [.01, .19]	-.08* [-.16, .00]	-.09 [-.18, .01]	-.10* [-.18, -.02]	.11* [.02, .20]	-.09* [-.16, -.02]				
Item 48	.10* [.01, .19]	-.11* [-.19, -.04]	-.07 [-.16, .03]	-.13* [-.21, -.04]	.07 [-.02, .17]	-.06 [-.13, .02]	.14* [.06, .23]			
Item 49	-.12* [-.20, -.03]	.02 [-.06, .10]	.26* [.16, .35]	.21* [.13, .29]	-.01 [-.11, .08]	.06 [-.01, .14]	-.07 [-.15, .02]	-.01 [-.10, .08]		
Item 50	.11* [.02, .20]	-.02 [-.09, .06]	-.11* [-.19, -.02]	-.08* [-.16, .00]	.07 [-.02, .15]	-.05 [-.12, .02]	.05 [-.03, .13]	.11* [.03, .19]	-.14* [-.22, -.06]	

Sample D: Japanese

First-order factor loadings	Identification-self	Comprehension-self	Expression-self	Regulation-self	Utilization-self	Identification-other	Comprehension-other	Expression-other	Regulation-other	Utilization-other
Item 6	**.52* [.32, .72]**	.02 [-.10, .15]	.01 [-.06, .07]	.02 [-.08, .13]	.02 [-.07, .11]	-.02 [-.14, .09]	.00 [-.12, .12]	.01 [-.08, .10]	.00 [-.13, .13]	.01 [-.08, .11]
Item 16	**.52* [.31, .72]**	.03 [-.10, .15]	.01 [-.06, .07]	-.02 [-.12, .09]	.03 [-.06, .12]	.00 [-.12, .12]	.05 [-.07, .17]	-.01 [-.10, .09]	.00 [-.13, .12]	-.01 [-.10, .09]
Item 20	**.39* [.18, .59]**	.05 [-.07, .18]	.00 [-.06, .07]	.03 [-.07, .14]	-.01 [-.10, .08]	.02 [-.10, .13]	.00 [-.12, .12]	-.01 [-.10, .08]	-.01 [-.14, .12]	.01 [-.09, .10]
Item 48	**.54* [.39, .71]**	.02 [-.10, .14]	.00 [-.06, .07]	-.03 [-.13, .07]	.06 [-.03, .15]	.03 [-.08, .15]	.01 [-.10, .13]	.00 [-.09, .09]	-.01 [-.14, .11]	-.01 [-.10, .08]
Item 49	**.54* [.34, .73]**	.08 [-.05, .20]	.00 [-.07, .07]	.01 [-.09, .11]	-.02 [-.11, .07]	-.04 [-.16, .07]	.01 [-.11, .13]	-.02 [-.11, .07]	-.04 [-.16, .09]	.00 [-.10, .09]
Item 1	-.01 [-.11, .09]	**.69* [.56, .83]**	.00 [-.07, .06]	-.02 [-.12, .08]	-.04 [-.13, .05]	-.03 [-.14, .08]	.00 [-.12, .11]	.02 [-.07, .11]	.00 [-.13, .13]	.00 [-.09, .10]
Item 2	-.02 [-.13, .08]	**.58* [.40, .74]**	-.01 [-.07, .06]	.00 [-.10, .10]	-.04 [-.13, .05]	-.01 [-.13, .10]	.02 [-.10, .14]	-.01 [-.10, .08]	.03 [-.10, .16]	.00 [-.09, .09]
Item 10	.02 [-.08, .13]	**.54* [.37, .70]**	-.01 [-.07, .06]	.00 [-.10, .10]	.02 [-.07, .11]	.04 [-.07, .16]	-.02 [-.13, .10]	.01 [-.08, .10]	-.02 [-.15, .10]	-.01 [-.10, .09]
Item 26	.04 [-.06, .15]	**.56* [.40, .72]**	.01 [-.06, .08]	.06 [-.05, .16]	-.04 [-.14, .05]	-.01 [-.13, .10]	.00 [-.12, .12]	-.03 [-.12, .06]	.00 [-.13, .12]	.01 [-.09, .10]
Item 43	.05 [-.05, .16]	**.51* [.34, .68]**	.00 [-.06, .07]	.02 [-.08, .12]	.02 [-.07, .11]	.05 [-.06, .17]	-.03 [-.15, .09]	.00 [-.09, .09]	-.01 [-.13, .12]	-.01 [-.11, .08]
Item 8	.01 [-.09, .12]	.02 [-.10, .14]	**.63* [.43, .83]**	-.03 [-.14, .07]	.01 [-.08, .10]	.01 [-.11, .13]	.04 [-.08, .16]	-.01 [-.10, .08]	.01 [-.12, .14]	.00 [-.09, .10]
Item 17	.02 [-.08, .13]	-.01 [-.13, .11]	**.41* [.18, .62]**	.08 [-.02, .19]	.01 [-.09, .10]	-.02 [-.14, .09]	-.01 [-.13, .11]	.01 [-.08, .10]	-.02 [-.14, .11]	.00 [-.09, .10]
Item 25	.00 [-.10, .10]	-.02 [-.14, .09]	**.50* [.29, .71]**	.01 [-.10, .11]	-.02 [-.11, .07]	.00 [-.11, .11]	.01 [-.11, .12]	.00 [-.09, .10]	.03 [-.10, .16]	.01 [-.08, .11]
Item 38	-.02 [-.12, .07]	.00 [-.12, .11]	**.33* [.25, .46]**	-.02 [-.12, .08]	.00 [-.09, .09]	-.04 [-.16, .07]	-.01 [-.12, .11]	.02 [-.07, .11]	.02 [-.10, .15]	-.01 [-.11, .08]
Item 42	.00 [-.10, .10]	.00 [-.12, .11]	**.17 [-.04, .39]**	.04 [-.06, .15]	.01 [-.08, .10]	.07 [-.04, .19]	.02 [-.09, .14]	.04 [-.05, .14]	.03 [-.10, .15]	.01 [-.08, .11]
Item 12	.00 [-.10, .09]	.01 [-.10, .12]	.00 [-.06, .07]	**.65* [.56, .76]**	.02 [-.07, .10]	.02 [-.09, .13]	.02 [-.10, .14]	.04 [-.05, .13]	.01 [-.12, .13]	.02 [-.07, .11]

Item 15	-.01 [-.11, .09]	-.03 [-.15, .08]	-.01 [-.07, .06]	**.67* [.54, .81]**	.01 [-.08, .10]	-.02 [-.13, .10]	.03 [-.09, .15]	-.02 [-.11, .07]	.03 [-.09, .16]	.01 [-.08, .10]
Item 37	.03 [-.06, .13]	.07 [-.04, .18]	.02 [-.05, .09]	**.54* [.40, .67]**	-.01 [-.10, .08]	.00 [-.11, .12]	.02 [-.10, .14]	-.02 [-.10, .07]	-.01 [-.13, .12]	.01 [-.08, .10]
Item 39	-.01 [-.11, .08]	-.01 [-.13, .10]	-.01 [-.07, .06]	**.71* [.58, .84]**	-.02 [-.11, .07]	.00 [-.12, .11]	-.04 [-.15, .08]	.01 [-.08, .10]	-.01 [-.14, .11]	-.02 [-.11, .07]
Item 50	.02 [-.08, .12]	.02 [-.09, .13]	.01 [-.06, .07]	**.32* [.17, .47]**	.11* [.02, .21]	.03 [-.08, .15]	.03 [-.09, .15]	.00 [-.09, .09]	.02 [-.11, .15]	.03 [-.06, .13]
Item 9	.00 [-.09, .10]	.03 [-.09, .14]	.00 [-.06, .07]	-.05 [-.15, .06]	**.12 [-.06, .29]**	.02 [-.10, .13]	.03 [-.08, .15]	-.01 [-.10, .08]	.04 [-.09, .16]	.02 [-.07, .11]
Item 21	.00 [-.10, .10]	-.01 [-.13, .10]	.00 [-.07, .06]	.03 [-.08, .13]	**.56* [.49, .65]**	.02 [-.09, .14]	.02 [-.10, .14]	-.01 [-.10, .08]	-.01 [-.14, .12]	.00 [-.10, .09]
Item 22	.01 [-.08, .12]	.02 [-.10, .13]	.01 [-.06, .08]	.08 [-.03, .18]	**.38* [.22, .53]**	.00 [-.12, .11]	-.01 [-.12, .11]	.02 [-.07, .12]	.04 [-.08, .17]	.01 [-.09, .10]
Item 24	-.01 [-.11, .09]	-.05 [-.16, .07]	.00 [-.07, .07]	.00 [-.11, .10]	**.49* [.33, .63]**	-.01 [-.13, .10]	.01 [-.11, .13]	.01 [-.08, .10]	.05 [-.08, .18]	.02 [-.07, .12]
Item 41	.00 [-.10, .10]	-.01 [-.12, .11]	.00 [-.06, .07]	.01 [-.10, .11]	**.55* [.41, .68]**	-.05 [-.17, .07]	-.01 [-.13, .11]	.05 [-.04, .15]	.00 [-.13, .13]	-.01 [-.11, .08]
Item 7	.01 [-.09, .10]	.03 [-.08, .15]	.01 [-.06, .07]	-.02 [-.12, .08]	-.02 [-.11, .07]	**.59* [.40, .76]**	.03 [-.09, .15]	.00 [-.09, .09]	-.02 [-.15, .11]	.00 [-.09, .10]
Item 29	.00 [-.09, .10]	.03 [-.08, .14]	.00 [-.07, .06]	.00 [-.10, .09]	-.05 [-.13, .04]	**.68* [.55, .84]**	.00 [-.12, .12]	.01 [-.08, .09]	-.07 [-.21, .05]	-.02 [-.12, .07]
Item 30	-.01 [-.10, .09]	-.05 [-.16, .06]	-.01 [-.08, .05]	-.04 [-.13, .05]	.02 [-.07, .10]	**.65* [.49, .81]**	.08 [-.03, .21]	.02 [-.06, .11]	.08 [-.04, .21]	.02 [-.07, .12]
Item 40	-.01 [-.10, .09]	.00 [-.11, .11]	-.01 [-.07, .06]	.00 [-.09, .10]	-.05 [-.13, .04]	**.56* [.38, .73]**	.04 [-.08, .16]	.03 [-.06, .12]	-.02 [-.15, .11]	.01 [-.08, .10]
Item 44	.00 [-.10, .10]	.06 [-.05, .17]	.01 [-.06, .08]	.01 [-.09, .10]	-.01 [-.10, .07]	**.61* [.44, .78]**	.02 [-.09, .14]	-.01 [-.10, .08]	.00 [-.13, .13]	-.01 [-.10, .08]
Item 5	.02 [-.07, .12]	-.01 [-.12, .10]	.01 [-.05, .08]	-.06 [-.15, .04]	.00 [-.09, .09]	.09 [-.02, .22]	**.40* [.18, .61]**	-.01 [-.09, .08]	.01 [-.12, .14]	.02 [-.07, .12]
Item 13	.01 [-.08, .11]	.02 [-.09, .13]	.01 [-.05, .08]	.04 [-.06, .14]	-.01 [-.09, .08]	.01 [-.10, .12]	**.67* [.52, .85]**	-.04 [-.13, .05]	.01 [-.12, .13]	-.01 [-.10, .08]
Item 14	.03 [-.07, .13]	.01 [-.11, .12]	.00 [-.07, .07]	.05 [-.05, .14]	.00 [-.08, .09]	.10 [-.02, .22]	**.61* [.40, .81]**	.01 [-.08, .10]	-.01 [-.14, .12]	.01 [-.09, .10]
Item 18	.01 [-.09, .10]	.00 [-.11, .12]	-.01 [-.08, .05]	-.04 [-.14, .06]	.04 [-.05, .13]	.04 [-.07, .16]	**.46* [.24, .67]**	-.01 [-.10, .08]	-.03 [-.16, .10]	-.01 [-.11, .08]
Item 34	.02 [-.08, .12]	.01 [-.11, .12]	.01 [-.06, .07]	-.02 [-.12, .08]	.00 [-.09, .09]	.04 [-.08, .16]	**.39* [.16, .61]**	.01 [-.08, .10]	.05 [-.08, .19]	.00 [-.09, .10]
Item 23	-.01 [-.11, .09]	-.05 [-.16, .06]	-.01 [-.08, .05]	-.01 [-.11, .08]	.04 [-.05, .13]	.02 [-.09, .13]	-.03 [-.14, .09]	**.69* [.54, .84]**	.06 [-.07, .19]	.00 [-.09, .10]
Item 28	.02 [-.08, .11]	.07 [-.04, .18]	.01 [-.05, .08]	.04 [-.06, .14]	.02 [-.07, .11]	.02 [-.10, .13]	.06 [-.06, .17]	**.34* [.17, .51]**	.03 [-.09, .16]	.01 [-.08, .10]
Item 31	.01 [-.09, .10]	.02 [-.09, .13]	.01 [-.06, .07]	.02 [-.08, .12]	.01 [-.08, .10]	-.03 [-.14, .08]	-.02 [-.13, .10]	**.52* [.42, .65]**	.00 [-.12, .12]	.00 [-.09, .09]
Item 45	-.01 [-.11, .08]	-.03 [-.15, .08]	.00 [-.07, .07]	.04 [-.06, .14]	.01 [-.08, .10]	.03 [-.08, .15]	-.01 [-.13, .10]	**.68* [.53, .84]**	.05 [-.08, .18]	-.02 [-.11, .08]
Item 46	.01 [-.09, .10]	.00 [-.11, .11]	.01 [-.06, .07]	-.01 [-.11, .08]	-.02 [-.10, .07]	.02 [-.09, .13]	-.02 [-.13, .10]	**.39* [.23, .56]**	.01 [-.12, .14]	-.01 [-.10, .08]
Item 19	-.03 [-.13, .07]	-.01 [-.12, .10]	-.01 [-.07, .06]	-.01 [-.10, .08]	-.02 [-.10, .07]	-.05 [-.17, .06]	.01 [-.11, .12]	.02 [-.07, .11]	**.77* [.62, .94]**	.00 [-.09, .09]
Item 27	-.01 [-.11, .08]	.00 [-.11, .10]	.00 [-.07, .06]	-.03 [-.13, .07]	.02 [-.07, .11]	.02 [-.09, .14]	.02 [-.10, .14]	.05 [-.04, .15]	**.55* [.36, .74]**	.00 [-.09, .10]
Item 33	.02 [-.08, .12]	.00 [-.10, .11]	.01 [-.05, .08]	.01 [-.08, .11]	.02 [-.07, .10]	.04 [-.07, .16]	-.01 [-.13, .11]	.04 [-.05, .13]	**.61* [.42, .79]**	.05 [-.04, .15]
Item 35	-.01 [-.10, .09]	.00 [-.11, .11]	.01 [-.06, .07]	-.05 [-.15, .05]	.02 [-.07, .11]	.02 [-.10, .13]	.03 [-.09, .16]	.04 [-.05, .13]	**.50* [.30, .69]**	.03 [-.06, .13]
Item 47	.00 [-.10, .10]	-.01 [-.12, .10]	.00 [-.06, .07]	.06 [-.05, .16]	.03 [-.06, .12]	-.05 [-.17, .07]	-.03 [-.16, .08]	.01 [-.08, .10]	**.66* [.47, .85]**	.01 [-.09, .10]
Item 3	-.02 [-.12, .07]	-.02 [-.13, .09]	-.01 [-.07, .06]	.00 [-.10, .10]	-.04 [-.13, .06]	.00 [-.12, .12]	.00 [-.12, .11]	-.04 [-.14, .05]	.00 [-.13, .12]	**.49* [.36, .66]**
Item 4	.02 [-.08, .12]	.04 [-.07, .16]	.01 [-.06, .08]	.01 [-.09, .11]	.00 [-.09, .09]	-.01 [-.13, .11]	.01 [-.12, .13]	-.02 [-.11, .07]	.01 [-.12, .15]	**.61* [.39, .83]**
Item 11	-.02 [-.12, .08]	.00 [-.11, .11]	.00 [-.06, .07]	.11* [.01, .21]	.01 [-.08, .10]	-.03 [-.15, .09]	.02 [-.10, .14]	.00 [-.09, .09]	.04 [-.09, .18]	**.22 [-.02, .46]**
Item 32	.01 [-.09, .11]	-.01 [-.12, .10]	.00 [-.07, .07]	.05 [-.04, .15]	.00 [-.09, .09]	.03 [-.09, .15]	-.01 [-.13, .11]	.00 [-.09, .09]	.19* [.05, .33]	**.50* [.28, .71]**
Item 36	.02 [-.08, .12]	.01 [-.10, .12]	-.01 [-.07, .06]	.04 [-.06, .14]	.02 [-.08, .11]	-.03 [-.14, .09]	-.01 [-.14, .11]	.01 [-.08, .10]	.01 [-.13, .14]	**.29* [.06, .52]**

Second-order factor loadings	Intrapersonal EC	Interpersonal EC
Identification-self	**.98* [.80, 1.22]**	-.07 [-.40, .19]
Comprehension-self	**.89* [.72, 1.08]**	-.06 [-.31, .17]

Expression-self	**.67* [.39, .96]**	.20 [-.16, .49]								
Regulation-self	**.58* [.35, .79]**	.09 [-.15, .30]								
Utilization-self	**.30 [-.04, .60]**	.14 [-.14, .39]								
Identification-other	.02 [-.22, .23]	**.83* [.65, 1.01]**								
Comprehension-other	.14 [-.09, .35]	**.77* [.57, .95]**								
Expression-other	-.09 [-.35, .15]	**.79* [.58, .99]**								
Regulation-other	-.07 [-.29, .13]	**.95* [.80, 1.11]**								
Utilization-other	.04 [-.26, .31]	**.85* [.60, 1.06]**								
Factor correlation	Interpersonal EC									
Intrapersonal EC	**.73* [.48, .89]**									
Residual correlation	Identification-self	Comprehension-self	Expression-self	Regulation-self	Utilization-self	Identification-other	Comprehension-other	Expression-other	Regulation-other	Utilization-other
Comprehension-self	.06 [-.08, .20]									
Expression-self	.01 [-.13, .15]	-.01 [-.14, .12]								
Regulation-self	-.01 [-.14, .13]	.01 [-.12, .13]	.02 [-.11, .14]							
Utilization-self	.01 [-.12, .14]	-.05 [-.18, .08]	.01 [-.12, .14]	.05 [-.07, .17]						
Identification-other	**.21* [.07, .33]**	.05 [-.08, .18]	-.01 [-.14, .12]	-.03 [-.16, .09]	-.04 [-.17, .08]					
Comprehension-other	.05 [-.09, .18]	**.30* [.17, .41]**	.01 [-.13, .14]	-.02 [-.14, .11]	-.02 [-.15, .11]	.11 [-.03, .24]				
Expression-other	-.01 [-.14, .13]	-.03 [-.15, .10]	**.16* [.03, .29]**	.04 [-.09, .16]	.06 [-.07, .18]	.02 [-.11, .15]	-.04 [-.17, .09]			
Regulation-other	-.03 [-.16, .11]	-.01 [-.15, .12]	.02 [-.11, .15]	**.39* [.28, .50]**	.05 [-.08, .18]	-.04 [-.17, .09]	-.02 [-.16, .11]	.09 [-.04, .22]		
Utilization-other	-.01 [-.14, .13]	-.01 [-.14, .12]	.00 [-.14, .13]	.05 [-.08, .18]	**.18* [.05, .31]**	-.03 [-.16, .11]	-.02 [-.15, .12]	-.03 [-.16, .11]	.08 [-.06, .22]	
	Item 1	Item 2	Item 3	Item 4	Item 5	Item 6	Item 7	Item 8	Item 9	Item 10
Item 2	.19* [.09, .28]									
Item 3	-.05 [-.14, .04]	-.01 [-.10, .08]								
Item 4	.03 [-.07, .12]	.05 [-.04, .15]	.13* [.04, .22]							
Item 5	.01 [-.08, .10]	.01 [-.07, .10]	-.03 [-.11, .06]	.03 [-.06, .11]						
Item 6	.00 [-.10, .09]	-.06 [-.14, .03]	.04 [-.05, .12]	.04 [-.05, .13]	.00 [-.09, .08]					
Item 7	.01 [-.09, .10]	-.02 [-.10, .07]	.05 [-.04, .13]	.01 [-.08, .10]	-.01 [-.09, .08]	.09* [.00, .18]				
Item 8	.04 [-.06, .14]	.02 [-.08, .11]	.01 [-.08, .10]	.07 [-.02, .16]	.00 [-.09, .09]	.07 [-.02, .17]	.08 [-.01, .17]			
Item 9	-.04 [-.13, .05]	-.05 [-.13, .04]	.01 [-.07, .09]	.00 [-.08, .08]	-.06 [-.13, .02]	.03 [-.05, .11]	-.02 [-.11, .06]	-.03 [-.11, .06]		
Item 10	.01 [-.09, .11]	-.05 [-.14, .05]	-.04 [-.13, .05]	.01 [-.08, .10]	-.04 [-.13, .05]	.11* [.02, .20]	.04 [-.05, .13]	-.03 [-.12, .07]	.09* [.00, .17]	
Item 11	-.05 [-.14, .04]	-.07 [-.15, .02]	.16* [.08, .24]	.02 [-.07, .12]	-.05 [-.13, .03]	.03 [-.05, .12]	.02 [-.07, .10]	.03 [-.06, .12]	-.02 [-.10, .06]	-.03 [-.11, .06]
Item 12	.01 [-.09, .11]	.03 [-.07, .12]	.02 [-.07, .11]	.03 [-.07, .13]	-.10* [-.19, -.01]	.02 [-.07, .12]	.04 [-.05, .14]	.02 [-.08, .12]	-.04 [-.13, .05]	.00 [-.09, .10]
Item 13	-.01 [-.11, .08]	.03 [-.07, .12]	.10* [.01, .19]	.00 [-.09, .10]	-.06 [-.15, .04]	.02 [-.08, .11]	.08 [-.01, .18]	.10* [.01, .20]	.04 [-.05, .12]	-.01 [-.11, .08]
Item 14	-.01 [-.11, .09]	.03 [-.07, .12]	.05 [-.04, .14]	.01 [-.09, .10]	-.07 [-.17, .02]	-.01 [-.10, .09]	.09 [-.01, .18]	.02 [-.08, .12]	.00 [-.09, .09]	.01 [-.08, .11]
Item 15	-.04 [-.14, .07]	-.03 [-.13, .07]	.05 [-.04, .15]	-.02 [-.11, .08]	.00 [-.09, .09]	.04 [-.05, .13]	-.02 [-.11, .08]	-.04 [-.13, .06]	.01 [-.08, .09]	-.03 [-.12, .07]
Item 16	-.03 [-.13, .06]	-.05 [-.14, .04]	-.07 [-.15, .02]	-.06 [-.15, .03]	.03 [-.06, .11]	.05 [-.04, .15]	.02 [-.07, .11]	.01 [-.08, .11]	.03 [-.05, .12]	.10* [.01, .19]

Item 17	-.01 [-.10, .08]	-.01 [-.09, .08]	.07 [-.02, .15]	.07 [-.02, .16]	-.07 [-.15, .01]	.04 [-.05, .12]	.01 [-.08, .10]	.03 [-.07, .13]	.00 [-.08, .08]	.00 [-.09, .09]
Item 18	.05 [-.04, .15]	.04 [-.05, .13]	-.04 [-.12, .04]	-.06 [-.15, .03]	.30* [.22, .38]	-.02 [-.10, .07]	-.01 [-.10, .08]	-.03 [-.12, .06]	-.08 [-.16, .00]	-.07 [-.16, .01]
Item 19	-.01 [-.11, .09]	.01 [-.08, .11]	.12* [.03, .22]	.08 [-.02, .18]	-.07 [-.16, .02]	.01 [-.09, .10]	.03 [-.06, .13]	.04 [-.06, .14]	.05 [-.04, .14]	-.05 [-.14, .05]
Item 20	.03 [-.07, .13]	.05 [-.04, .14]	-.09* [-.17, -.01]	.05 [-.04, .13]	.07 [-.02, .15]	-.02 [-.11, .07]	-.01 [-.10, .07]	.02 [-.07, .11]	-.03 [-.11, .05]	-.03 [-.11, .06]
Item 21	-.08 [-.18, .02]	-.01 [-.10, .09]	-.01 [-.10, .08]	-.06 [-.15, .04]	.01 [-.08, .10]	.06 [-.03, .16]	.01 [-.08, .11]	-.02 [-.12, .08]	-.01 [-.10, .09]	-.06 [-.15, .04]
Item 22	.00 [-.09, .10]	-.02 [-.11, .07]	-.06 [-.15, .02]	.08 [-.01, .17]	-.06 [-.14, .03]	.03 [-.05, .12]	.00 [-.09, .09]	.00 [-.09, .10]	.06 [-.03, .14]	.08 [-.01, .17]
Item 23	-.01 [-.12, .09]	-.04 [-.14, .06]	.03 [-.07, .13]	.00 [-.10, .10]	-.11* [-.20, -.01]	.03 [-.07, .12]	.04 [-.06, .13]	-.05 [-.15, .05]	.03 [-.07, .12]	-.02 [-.11, .09]
Item 24	-.09 [-.19, .01]	-.08 [-.17, .01]	.05 [-.04, .14]	.01 [-.09, .10]	-.02 [-.11, .07]	.00 [-.09, .10]	-.03 [-.12, .07]	-.02 [-.11, .08]	.01 [-.09, .10]	.08 [-.02, .17]
Item 25	-.01 [-.10, .09]	.03 [-.07, .11]	-.02 [-.10, .07]	.00 [-.09, .09]	.18* [.09, .26]	-.06 [-.14, .03]	-.06 [-.15, .02]	.10 [-.01, .20]	-.05 [-.13, .03]	-.06 [-.14, .03]
Item 26	-.02 [-.13, .08]	.05 [-.05, .14]	.05 [-.04, .14]	-.02 [-.11, .08]	.02 [-.07, .11]	.00 [-.09, .09]	-.04 [-.14, .05]	.04 [-.06, .14]	.01 [-.08, .09]	.02 [-.07, .12]
Item 27	.05 [-.04, .14]	.00 [-.09, .09]	-.06 [-.15, .02]	.01 [-.08, .10]	.04 [-.05, .12]	-.07 [-.16, .02]	-.04 [-.13, .05]	.00 [-.09, .09]	-.01 [-.09, .07]	-.04 [-.13, .05]
Item 28	.06 [-.04, .15]	-.01 [-.09, .08]	-.12* [-.20, -.04]	-.07 [-.16, .01]	.05 [-.03, .13]	-.06 [-.14, .03]	-.04 [-.13, .04]	-.01 [-.10, .08]	-.06 [-.14, .02]	.05 [-.04, .13]
Item 29	.00 [-.10, .10]	.01 [-.08, .11]	-.07 [-.16, .02]	-.05 [-.14, .04]	.16* [.08, .25]	-.06 [-.15, .03]	-.05 [-.14, .05]	-.07 [-.16, .03]	-.04 [-.12, .05]	.05 [-.04, .14]
Item 30	-.07 [-.17, .04]	-.02 [-.12, .08]	.02 [-.08, .12]	-.01 [-.11, .09]	.01 [-.09, .11]	-.04 [-.14, .05]	.10 [.00, .20]	-.01 [-.11, .10]	.00 [-.09, .09]	-.03 [-.13, .06]
Item 31	.07 [-.03, .16]	-.01 [-.10, .08]	-.10* [-.19, -.02]	.02 [-.07, .11]	.06 [-.03, .14]	-.02 [-.11, .07]	-.03 [-.11, .06]	.02 [-.07, .11]	-.09* [-.17, -.01]	.03 [-.06, .12]
Item 32	-.02 [-.12, .08]	.03 [-.06, .13]	-.02 [-.12, .07]	.05 [-.05, .15]	-.08 [-.17, .01]	.01 [-.08, .11]	-.02 [-.12, .07]	-.01 [-.10, .09]	.05 [-.03, .14]	-.06 [-.15, .04]
Item 33	-.04 [-.14, .06]	.00 [-.09, .10]	-.05 [-.14, .05]	.00 [-.10, .10]	-.06 [-.15, .03]	-.01 [-.10, .09]	-.06 [-.16, .03]	.02 [-.08, .12]	.07 [-.02, .15]	-.03 [-.12, .07]
Item 34	.01 [-.08, .10]	.00 [-.09, .08]	.01 [-.07, .09]	.00 [-.08, .09]	.00 [-.08, .09]	-.03 [-.12, .05]	-.05 [-.13, .04]	.01 [-.08, .09]	.04 [-.04, .11]	.02 [-.07, .10]
Item 35	-.05 [-.15, .05]	-.02 [-.11, .07]	.10* [.01, .18]	.02 [-.07, .12]	-.08 [-.17, .00]	.08 [-.01, .17]	-.02 [-.10, .07]	.02 [-.07, .12]	.01 [-.08, .09]	.02 [-.07, .11]
Item 36	-.04 [-.13, .05]	-.09* [-.17, .00]	.16* [.08, .24]	.02 [-.07, .11]	-.06 [-.14, .02]	.15* [.07, .23]	-.05 [-.14, .03]	-.05 [-.14, .04]	.11* [.03, .19]	.07 [-.01, .16]
Item 37	.05 [-.05, .14]	.07 [-.03, .16]	.05 [-.04, .14]	.05 [-.04, .14]	.01 [-.08, .10]	-.04 [-.13, .05]	-.07 [-.16, .02]	.01 [-.08, .11]	-.05 [-.13, .04]	-.05 [-.14, .04]
Item 38	.08 [-.01, .17]	.02 [-.06, .11]	-.04 [-.12, .04]	-.05 [-.13, .04]	.13* [.05, .21]	-.06 [-.14, .03]	-.07 [-.15, .01]	.02 [-.07, .10]	-.05 [-.13, .02]	-.06 [-.14, .03]
Item 39	-.03 [-.13, .07]	-.03 [-.12, .07]	.01 [-.08, .10]	-.04 [-.14, .06]	-.07 [-.16, .03]	.01 [-.09, .11]	.03 [-.07, .12]	-.04 [-.14, .06]	-.04 [-.13, .05]	.04 [-.06, .14]
Item 40	.01 [-.09, .11]	.00 [-.09, .09]	-.08 [-.16, .01]	-.02 [-.11, .07]	.20* [.11, .28]	-.04 [-.13, .05]	.01 [-.09, .10]	-.07 [-.16, .03]	-.05 [-.13, .03]	.07 [-.02, .16]
Item 41	.00 [-.10, .11]	-.08 [-.17, .02]	-.02 [-.11, .07]	-.03 [-.13, .07]	-.08 [-.17, .01]	.05 [-.04, .15]	-.02 [-.11, .08]	.01 [-.09, .11]	.06 [-.04, .16]	.06 [-.04, .15]
Item 42	.02 [-.07, .11]	.01 [-.08, .09]	-.05 [-.13, .03]	-.06 [-.15, .02]	.14* [.06, .22]	-.05 [-.13, .03]	.00 [-.08, .08]	-.07 [-.17, .03]	-.04 [-.11, .04]	-.03 [-.11, .06]
Item 43	.09 [-.01, .20]	-.03 [-.13, .06]	-.13* [-.21, -.04]	-.02 [-.12, .07]	.10* [.01, .18]	-.03 [-.12, .07]	.02 [-.07, .11]	-.02 [-.11, .08]	-.01 [-.09, .08]	.06 [-.04, .16]
Item 44	.07 [-.03, .17]	.00 [-.09, .10]	-.08 [-.17, .00]	-.02 [-.12, .07]	.17* [.08, .26]	-.05 [-.14, .05]	.02 [-.07, .12]	.00 [-.09, .10]	-.04 [-.12, .05]	.00 [-.10, .09]
Item 45	-.01 [-.12, .09]	.00 [-.09, .10]	-.05 [-.14, .05]	-.07 [-.17, .03]	-.03 [-.12, .07]	.03 [-.07, .12]	.02 [-.08, .12]	-.05 [-.15, .05]	.06 [-.04, .15]	.01 [-.09, .11]
Item 46	.04 [-.05, .13]	-.06 [-.15, .02]	-.11* [-.19, -.03]	-.04 [-.13, .05]	.06 [-.03, .14]	-.03 [-.12, .05]	-.06 [-.15, .02]	.02 [-.07, .11]	-.12* [-.20, -.04]	.06 [-.03, .15]
Item 47	.01 [-.09, .11]	.04 [-.05, .14]	.02 [-.07, .11]	.02 [-.08, .11]	-.04 [-.13, .05]	-.04 [-.13, .06]	-.01 [-.10, .08]	-.04 [-.13, .06]	.09* [.01, .17]	-.02 [-.11, .07]
Item 48	.02 [-.07, .12]	-.04 [-.13, .05]	-.01 [-.10, .08]	-.07 [-.16, .02]	-.04 [-.12, .05]	.06 [-.03, .15]	-.04 [-.12, .06]	-.02 [-.11, .08]	.07 [-.02, .15]	.10* [.01, .19]
Item 49	.02 [-.08, .12]	.01 [-.08, .10]	-.01 [-.09, .08]	.03 [-.06, .13]	.02 [-.07, .11]	-.06 [-.15, .04]	-.07 [-.16, .02]	-.02 [-.11, .08]	-.02 [-.11, .06]	.01 [-.09, .10]
Item 50	-.06 [-.15, .03]	-.04 [-.12, .05]	.03 [-.05, .11]	.03 [-.06, .12]	-.02 [-.10, .06]	.10* [.02, .19]	-.03 [-.11, .06]	-.02 [-.11, .07]	-.01 [-.09, .08]	.08 [-.01, .17]
	Item 11	Item 12	Item 13	Item 14	Item 15	Item 16	Item 17	Item 18	Item 19	Item 20
Item 12	.08 [-.01, .17]									
Item 13	.13* [.04, .21]	.06 [-.04, .16]								

Item 14	.05 [-.04, .14]	.05 [-.05, .15]	.15* [.05, .25]							
Item 15	.13* [.03, .21]	.01 [-.09, .11]	.08 [-.02, .17]	.02 [-.08, .12]						
Item 16	-.05 [-.13, .04]	-.01 [-.11, .08]	.05 [-.05, .14]	.08 [-.01, .17]	-.02 [-.11, .08]					
Item 17	.04 [-.05, .12]	.10* [.01, .19]	.08 [-.01, .17]	.02 [-.07, .11]	-.01 [-.10, .08]	.01 [-.08, .09]				
Item 18	-.05 [-.13, .03]	-.11* [-.20, -.02]	-.05 [-.15, .04]	-.03 [-.13, .06]	-.01 [-.10, .09]	.02 [-.07, .10]	-.11* [-.19, -.03]			
Item 19	.13* [.04, .22]	.05 [-.05, .15]	.14* [.04, .23]	.01 [-.09, .11]	.05 [-.05, .16]	-.07 [-.16, .03]	-.03 [-.12, .07]	-.12* [-.21, -.02]		
Item 20	-.09* [-.17, -.01]	.07 [-.02, .16]	-.10* [-.19, -.01]	-.05 [-.14, .05]	-.04 [-.13, .06]	-.05 [-.14, .04]	-.02 [-.10, .07]	.05 [-.03, .13]	-.06 [-.15, .04]	
Item 21	.05 [-.04, .14]	.00 [-.10, .10]	.06 [-.04, .16]	.05 [-.05, .14]	.04 [-.06, .14]	.07 [-.03, .16]	.02 [-.07, .11]	.03 [-.07, .12]	-.05 [-.15, .05]	-.07 [-.16, .03]
Item 22	-.03 [-.11, .06]	.12* [.03, .21]	-.07 [-.16, .03]	-.01 [-.11, .08]	-.05 [-.14, .05]	.00 [-.09, .09]	.07 [-.01, .16]	-.05 [-.13, .04]	-.01 [-.11, .08]	-.01 [-.09, .08]
Item 23	.05 [-.04, .15]	.01 [-.10, .11]	.02 [-.08, .12]	.03 [-.08, .13]	.02 [-.09, .12]	.01 [-.09, .11]	.02 [-.08, .11]	-.12* [-.21, -.03]	.09 [-.02, .19]	-.07 [-.16, .03]
Item 24	.04 [-.04, .13]	-.04 [-.14, .06]	-.02 [-.12, .08]	.00 [-.10, .10]	.03 [-.07, .13]	.08 [-.02, .17]	-.01 [-.10, .08]	-.03 [-.12, .06]	.05 [-.05, .15]	-.05 [-.14, .04]
Item 25	.07 [-.01, .15]	-.06 [-.15, .03]	-.06 [-.15, .04]	-.09* [-.18, .00]	.03 [-.06, .13]	-.01 [-.09, .08]	-.05 [-.14, .04]	.13* [.04, .21]	.02 [-.08, .11]	.04 [-.05, .13]
Item 26	.03 [-.06, .12]	.01 [-.09, .11]	.00 [-.10, .09]	-.03 [-.12, .07]	.00 [-.09, .11]	.00 [-.10, .09]	-.02 [-.11, .07]	.05 [-.04, .14]	-.01 [-.11, .10]	.13* [.04, .22]
Item 27	-.17* [-.25, -.09]	-.03 [-.12, .07]	-.05 [-.14, .04]	-.03 [-.12, .06]	-.04 [-.13, .05]	-.02 [-.11, .07]	-.03 [-.12, .05]	.07 [-.02, .16]	-.05 [-.14, .05]	.07 [-.01, .16]
Item 28	-.06 [-.14, .02]	.05 [-.05, .14]	-.09 [-.17, .00]	.02 [-.07, .11]	-.03 [-.12, .06]	-.03 [-.11, .06]	.03 [-.05, .11]	.10* [.02, .18]	-.10* [-.19, -.01]	.06 [-.02, .14]
Item 29	-.06 [-.15, .03]	-.04 [-.14, .06]	-.02 [-.11, .08]	-.03 [-.13, .07]	-.03 [-.13, .07]	.00 [-.09, .09]	-.04 [-.13, .05]	.13* [.04, .22]	-.11* [-.20, -.01]	.11* [.02, .20]
Item 30	-.02 [-.11, .08]	-.02 [-.13, .08]	.04 [-.06, .14]	.16* [.06, .26]	-.06 [-.17, .04]	.06 [-.04, .16]	.01 [-.09, .10]	.00 [-.10, .09]	-.03 [-.13, .08]	-.05 [-.14, .05]
Item 31	-.12* [-.20, -.04]	-.01 [-.10, .09]	-.13* [-.21, -.04]	-.04 [-.13, .05]	-.06 [-.15, .04]	-.04 [-.13, .05]	-.01 [-.09, .08]	.07 [-.02, .15]	-.05 [-.14, .05]	.04 [-.05, .12]
Item 32	.05 [-.05, .14]	.03 [-.07, .13]	-.04 [-.14, .06]	-.02 [-.12, .08]	.03 [-.07, .13]	.00 [-.09, .10]	-.02 [-.11, .07]	-.10* [-.19, -.01]	.09 [-.01, .19]	.02 [-.07, .12]
Item 33	.03 [-.06, .12]	.03 [-.07, .13]	-.04 [-.14, .06]	-.01 [-.11, .09]	.03 [-.08, .13]	.05 [-.05, .14]	-.01 [-.10, .08]	-.07 [-.16, .02]	.01 [-.10, .12]	-.04 [-.13, .05]
Item 34	-.01 [-.09, .07]	-.09 [-.18, .01]	.02 [-.07, .11]	-.06 [-.15, .03]	.01 [-.09, .10]	-.05 [-.13, .04]	-.03 [-.11, .05]	.07 [-.02, .15]	.07 [-.03, .16]	.02 [-.06, .10]
Item 35	.10* [.02, .19]	.00 [-.09, .10]	.02 [-.07, .11]	.03 [-.07, .12]	.02 [-.07, .12]	.02 [-.07, .10]	-.01 [-.09, .08]	-.07 [-.15, .02]	.04 [-.06, .14]	-.08 [-.16, .01]
Item 36	.04 [-.04, .12]	.02 [-.07, .11]	-.01 [-.10, .08]	-.01 [-.10, .08]	.03 [-.06, .12]	-.01 [-.09, .08]	.01 [-.07, .09]	-.03 [-.11, .05]	-.03 [-.13, .06]	-.04 [-.12, .04]
Item 37	.06 [-.02, .15]	-.01 [-.11, .09]	-.03 [-.12, .07]	.00 [-.09, .10]	.06 [-.04, .16]	-.04 [-.13, .05]	.03 [-.05, .12]	.13* [.04, .21]	.01 [-.09, .11]	.05 [-.04, .14]
Item 38	-.07 [-.15, .01]	-.03 [-.12, .06]	-.04 [-.13, .05]	-.07 [-.15, .02]	.02 [-.07, .11]	.03 [-.06, .11]	-.02 [-.10, .06]	.06 [-.02, .14]	.00 [-.09, .09]	.04 [-.04, .12]
Item 39	.05 [-.04, .14]	.06 [-.04, .16]	.01 [-.09, .11]	.05 [-.06, .15]	.10 [-.01, .21]	.02 [-.07, .12]	.08 [-.01, .17]	-.11* [-.20, -.02]	.01 [-.09, .12]	-.07 [-.16, .03]
Item 40	-.11* [-.19, -.03]	-.03 [-.13, .06]	-.06 [-.15, .04]	-.04 [-.14, .05]	-.03 [-.13, .07]	-.02 [-.11, .07]	-.06 [-.14, .03]	.19* [.10, .27]	-.10* [-.20, .00]	.06 [-.03, .14]
Item 41	-.04 [-.13, .05]	.00 [-.10, .10]	-.01 [-.11, .09]	.00 [-.10, .10]	.01 [-.10, .11]	.02 [-.08, .11]	-.02 [-.11, .08]	-.02 [-.11, .07]	-.01 [-.11, .10]	-.05 [-.14, .04]
Item 42	-.07 [-.15, .01]	-.02 [-.11, .07]	-.08 [-.17, .00]	-.05 [-.14, .04]	.00 [-.09, .09]	.00 [-.08, .08]	-.02 [-.10, .07]	.11* [.03, .19]	-.08 [-.17, .01]	.05 [-.04, .13]
Item 43	-.09* [-.18, -.01]	-.01 [-.10, .09]	-.07 [-.16, .03]	-.08 [-.17, .02]	-.05 [-.14, .05]	.02 [-.07, .12]	-.06 [-.14, .03]	.09* [.00, .18]	-.09 [-.19, .02]	.21* [.12, .29]
Item 44	-.11* [-.20, -.03]	-.04 [-.14, .06]	-.07 [-.16, .03]	-.01 [-.10, .09]	-.04 [-.13, .07]	-.03 [-.13, .06]	-.07 [-.15, .02]	.16* [.07, .24]	-.08 [-.18, .02]	.12* [.03, .21]
Item 45	-.03 [-.13, .06]	.04 [-.06, .15]	-.01 [-.11, .09]	.00 [-.10, .11]	-.02 [-.12, .09]	.03 [-.07, .13]	-.01 [-.10, .08]	-.02 [-.12, .07]	.05 [-.06, .15]	-.03 [-.12, .07]
Item 46	-.10* [-.18, -.02]	.06 [-.03, .15]	-.09 [-.17, .00]	-.08 [-.17, .02]	-.09 [-.18, .00]	-.02 [-.11, .06]	.05 [-.03, .13]	.07 [-.01, .15]	-.08 [-.17, .01]	.04 [-.04, .13]
Item 47	.02 [-.07, .11]	-.05 [-.15, .05]	.01 [-.09, .10]	-.06 [-.15, .04]	.08 [-.02, .18]	-.02 [-.11, .08]	.01 [-.08, .09]	-.03 [-.12, .06]	.19* [.09, .29]	-.07 [-.16, .02]
Item 48	-.10* [-.18, -.01]	-.04 [-.14, .05]	.00 [-.10, .09]	.03 [-.07, .12]	-.02 [-.12, .08]	.05 [-.05, .14]	.04 [-.04, .13]	-.05 [-.14, .04]	-.07 [-.17, .03]	-.06 [-.15, .03]
Item 49	-.03 [-.11, .06]	-.06 [-.15, .04]	-.03 [-.12, .07]	-.02 [-.11, .08]	.01 [-.08, .11]	.01 [-.09, .10]	.03 [-.06, .12]	.08 [-.01, .17]	-.04 [-.14, .06]	.09 [.00, .18]
Item 50	.00 [-.08, .08]	.02 [-.07, .12]	-.02 [-.11, .07]	-.01 [-.10, .08]	.01 [-.09, .11]	.01 [-.08, .09]	.06 [-.03, .14]	-.04 [-.12, .05]	-.04 [-.13, .06]	-.05 [-.13, .04]

	Item 21	Item 22	Item 23	Item 24	Item 25	Item 26	Item 27	Item 28	Item 29	Item 30
Item 22	.03 [-.07, .13]									
Item 23	.04 [-.07, .14]	.03 [-.07, .13]								
Item 24	.05 [-.05, .15]	.08 [-.03, .18]	.06 [-.04, .16]							
Item 25	-.03 [-.12, .07]	-.05 [-.13, .04]	-.10* [-.19, .00]	-.02 [-.11, .07]						
Item 26	-.02 [-.11, .08]	-.10* [-.19, -.01]	-.10 [-.20, .01]	-.12* [-.21, -.02]	.06 [-.03, .16]					
Item 27	.00 [-.09, .10]	-.03 [-.11, .06]	-.03 [-.13, .06]	-.05 [-.14, .04]	.03 [-.06, .11]	.05 [-.04, .14]				
Item 28	-.01 [-.10, .09]	.05 [-.04, .13]	-.10 [-.20, .00]	-.04 [-.13, .05]	.06 [-.02, .15]	.00 [-.09, .09]	.10* [.01, .18]			
Item 29	-.04 [-.13, .06]	-.02 [-.11, .08]	-.08 [-.17, .02]	-.10* [-.19, .00]	.11* [.02, .20]	.08 [-.02, .17]	.06 [-.04, .15]	.05 [-.03, .14]		
Item 30	.05 [-.06, .15]	.02 [-.08, .12]	.04 [-.07, .14]	.03 [-.08, .13]	-.10* [-.20, -.01]	-.04 [-.14, .07]	.01 [-.08, .11]	-.01 [-.11, .08]	.00 [-.10, .10]	
Item 31	-.09 [-.18, .01]	.03 [-.06, .12]	-.07 [-.16, .03]	-.01 [-.10, .08]	.05 [-.04, .14]	.02 [-.07, .11]	.17* [.08, .25]	.22* [.13, .30]	.10* [.01, .19]	-.06 [-.16, .04]
Item 32	.03 [-.07, .13]	.02 [-.08, .11]	.02 [-.09, .12]	.04 [-.06, .14]	.01 [-.09, .10]	.03 [-.07, .12]	.00 [-.09, .09]	-.02 [-.11, .07]	-.06 [-.15, .04]	.03 [-.07, .14]
Item 33	.01 [-.09, .11]	.08 [-.01, .18]	.08 [-.03, .18]	.05 [-.05, .15]	-.06 [-.15, .04]	-.02 [-.12, .08]	.05 [-.05, .14]	.00 [-.09, .09]	-.05 [-.14, .05]	.12* [.02, .22]
Item 34	-.05 [-.14, .04]	-.05 [-.13, .04]	-.03 [-.12, .07]	.01 [-.08, .10]	.10* [.02, .19]	.10* [.01, .19]	.06 [-.03, .14]	.03 [-.05, .11]	.02 [-.07, .11]	.00 [-.09, .09]
Item 35	.03 [-.07, .12]	.03 [-.06, .11]	.16* [.06, .25]	.08 [-.01, .18]	-.05 [-.14, .03]	-.07 [-.16, .03]	-.03 [-.12, .05]	-.07 [-.15, .02]	-.13* [-.22, -.04]	.09 [-.01, .19]
Item 36	.01 [-.09, .09]	.02 [-.07, .10]	.05 [-.05, .14]	.10* [.01, .18]	-.06 [-.15, .02]	.00 [-.09, .09]	-.06 [-.14, .02]	.00 [-.08, .08]	-.11* [-.19, -.02]	.00 [-.09, .10]
Item 37	.00 [-.10, .10]	-.06 [-.15, .03]	-.07 [-.17, .03]	-.05 [-.14, .05]	.14* [.05, .23]	.09 [-.01, .18]	.01 [-.08, .10]	-.01 [-.10, .08]	.06 [-.03, .16]	-.08 [-.18, .02]
Item 38	-.02 [-.11, .07]	-.06 [-.14, .03]	-.03 [-.12, .06]	.01 [-.07, .10]	.10* [.02, .19]	.05 [-.04, .13]	.03 [-.05, .11]	.04 [-.04, .12]	.07 [-.02, .15]	-.08 [-.17, .01]
Item 39	.02 [-.09, .12]	-.01 [-.10, .09]	.04 [-.06, .15]	.01 [-.09, .11]	-.07 [-.17, .02]	.02 [-.08, .12]	-.04 [-.13, .06]	-.02 [-.11, .07]	-.03 [-.13, .07]	.04 [-.06, .15]
Item 40	-.05 [-.14, .05]	-.08 [-.16, .02]	-.07 [-.16, .03]	-.06 [-.15, .04]	.07 [-.02, .16]	.02 [-.07, .12]	.10* [.01, .19]	.10* [.02, .19]	.19* [.10, .28]	-.04 [-.14, .06]
Item 41	.08 [-.02, .18]	.06 [-.04, .17]	.14* [.03, .24]	.12* [.01, .23]	-.12* [-.21, -.03]	-.09 [-.19, .01]	.03 [-.07, .12]	-.03 [-.12, .06]	-.10* [-.20, .00]	.03 [-.08, .13]
Item 42	-.06 [-.15, .03]	-.01 [-.09, .07]	-.06 [-.15, .03]	-.01 [-.09, .08]	.05 [-.03, .14]	.00 [-.09, .09]	.06 [-.02, .14]	.10* [.02, .18]	.13* [.04, .21]	-.04 [-.13, .06]
Item 43	-.02 [-.11, .08]	-.01 [-.10, .08]	-.09 [-.19, .01]	-.07 [-.16, .03]	.07 [-.02, .16]	.08 [-.02, .18]	.06 [-.04, .15]	.15* [.06, .24]	.12* [.03, .22]	-.07 [-.17, .03]
Item 44	.00 [-.10, .10]	-.06 [-.15, .04]	-.08 [-.18, .03]	-.08 [-.17, .02]	.09 [.00, .18]	.00 [-.10, .10]	.09 [-.01, .18]	.14* [.05, .23]	.19* [.09, .28]	.00 [-.11, .10]
Item 45	.04 [-.07, .14]	.02 [-.08, .12]	.26* [.15, .37]	.02 [-.08, .12]	-.06 [-.15, .04]	-.09 [-.19, .01]	.02 [-.08, .11]	-.08 [-.18, .02]	.01 [-.09, .11]	.06 [-.05, .17]
Item 46	-.07 [-.16, .02]	.06 [-.03, .14]	-.12* [-.21, -.02]	-.06 [-.14, .03]	.03 [-.06, .11]	-.01 [-.11, .08]	.12* [.03, .20]	.25* [.17, .33]	.10* [.01, .19]	.00 [-.09, .10]
Item 47	.01 [-.08, .11]	-.01 [-.10, .09]	.07 [-.03, .17]	.07 [-.02, .17]	.04 [-.05, .13]	-.06 [-.16, .04]	-.02 [-.11, .07]	-.03 [-.11, .06]	-.08 [-.18, .01]	-.01 [-.12, .09]
Item 48	.01 [-.08, .11]	.05 [-.04, .14]	.04 [-.06, .14]	.08 [-.01, .17]	-.02 [-.11, .07]	-.02 [-.12, .07]	-.02 [-.10, .07]	-.03 [-.12, .05]	.00 [-.10, .09]	.11* [.01, .21]
Item 49	-.03 [-.13, .06]	-.05 [-.14, .04]	-.04 [-.14, .06]	-.07 [-.16, .03]	.08 [-.02, .16]	.20* [.11, .29]	.01 [-.08, .10]	.04 [-.05, .13]	.04 [-.05, .14]	-.01 [-.11, .09]
Item 50	.09* [.00, .18]	.17* [.09, .26]	.03 [-.07, .12]	.14* [.05, .22]	-.08 [-.16, .00]	-.06 [-.15, .03]	-.03 [-.12, .05]	-.05 [-.13, .04]	-.02 [-.11, .07]	.03 [-.07, .12]
	Item 31	Item 32	Item 33	Item 34	Item 35	Item 36	Item 37	Item 38	Item 39	Item 40
Item 32	-.04 [-.13, .05]									
Item 33	-.07 [-.16, .03]	.30* [.21, .39]								
Item 34	.05 [-.04, .13]	-.03 [-.12, .06]	-.08 [-.17, .01]							
Item 35	-.08 [-.17, .00]	.07 [-.03, .16]	.11* [.01, .21]	.09* [.00, .17]						
Item 36	-.06 [-.14, .02]	.04 [-.06, .13]	.01 [-.08, .10]	.01 [-.07, .09]	.22* [.14, .30]					
Item 37	.01 [-.08, .10]	-.05 [-.15, .04]	-.10* [-.20, -.01]	.06 [-.03, .14]	-.03 [-.12, .07]	-.01 [-.10, .07]				

Item 38	.12* [.04, .20]	-.05 [-.14, .04]	-.02 [-.11, .07]	.05 [-.03, .13]	.00 [-.08, .08]	-.08* [-.16, -.01]	.04 [-.05, .12]			
Item 39	-.01 [-.10, .09]	.00 [-.10, .10]	.01 [-.09, .11]	-.06 [-.15, .03]	-.05 [-.15, .04]	-.01 [-.10, .08]	.05 [-.05, .15]	-.10* [-.18, -.01]		
Item 40	.14* [.05, .23]	-.06 [-.15, .04]	-.10* [-.19, .00]	.05 [-.04, .14]	-.16* [-.24, -.07]	-.04 [-.12, .04]	.05 [-.04, .14]	.07 [-.02, .15]	-.05 [-.15, .05]	
Item 41	.04 [-.05, .13]	-.03 [-.14, .07]	.01 [-.09, .11]	.01 [-.08, .10]	.09 [-.01, .18]	.06 [-.03, .15]	-.09 [-.18, .01]	.00 [-.09, .09]	.03 [-.08, .13]	-.08 [-.17, .02]
Item 42	.16* [.08, .24]	-.08 [-.17, .00]	-.05 [-.13, .04]	.02 [-.06, .10]	-.09* [-.17, -.01]	-.10* [-.18, -.02]	.00 [-.09, .09]	.22* [.14, .29]	.04 [-.05, .13]	.19* [.11, .27]
Item 43	.09* [.00, .18]	-.11* [-.20, -.01]	-.08 [-.18, .02]	-.05 [-.13, .04]	-.12* [-.20, -.02]	-.07 [-.15, .02]	.07 [-.03, .16]	.02 [-.06, .11]	-.03 [-.13, .07]	.14* [.05, .23]
Item 44	.05 [-.04, .15]	-.13* [-.23, -.04]	-.09 [-.18, .01]	.02 [-.07, .11]	-.10* [-.19, -.01]	-.07 [-.15, .02]	.06 [-.04, .15]	.06 [-.02, .15]	-.05 [-.15, .05]	.23* [.13, .32]
Item 45	-.09 [-.19, .00]	-.01 [-.12, .09]	.08 [-.03, .18]	-.05 [-.14, .04]	.05 [-.05, .15]	.03 [-.07, .12]	-.04 [-.14, .06]	.04 [-.06, .13]	.04 [-.07, .14]	-.04 [-.14, .06]
Item 46	.32* [.24, .39]	-.07 [-.16, .02]	-.02 [-.11, .07]	-.01 [-.09, .07]	-.09* [-.17, -.01]	-.03 [-.11, .05]	-.05 [-.14, .04]	.02 [-.06, .10]	-.02 [-.11, .08]	.19* [.10, .27]
Item 47	-.02 [-.11, .07]	.13* [.03, .23]	.03 [-.08, .13]	.05 [-.04, .14]	.05 [-.05, .14]	.09* [.01, .18]	.02 [-.07, .12]	.02 [-.07, .11]	.07 [-.03, .17]	-.09 [-.18, .00]
Item 48	.01 [-.08, .10]	-.01 [-.10, .09]	.03 [-.06, .13]	.03 [-.06, .11]	.04 [-.05, .13]	.06 [-.03, .14]	-.07 [-.16, .02]	-.04 [-.12, .05]	.03 [-.06, .13]	-.02 [-.11, .07]
Item 49	.00 [-.09, .09]	-.03 [-.12, .07]	-.05 [-.14, .05]	.08 [.00, .17]	-.12* [-.21, -.03]	.00 [-.09, .09]	.14* [.05, .23]	-.06 [-.15, .02]	.01 [-.09, .11]	.08 [-.02, .17]
Item 50	-.01 [-.09, .08]	.04 [-.05, .13]	.05 [-.05, .14]	-.02 [-.10, .06]	.05 [-.04, .13]	.09* [.01, .17]	-.03 [-.12, .06]	-.03 [-.11, .05]	.05 [-.05, .15]	-.01 [-.10, .08]
	Item 41	Item 42	Item 43	Item 44	Item 45	Item 46	Item 47	Item 48	Item 49	Item 50
Item 42	.01 [-.08, .10]									
Item 43	-.04 [-.14, .06]	.16* [.08, .24]								
Item 44	-.08 [-.18, .02]	.18* [.10, .27]	.27* [.18, .36]							
Item 45	.10 [-.01, .20]	.02 [-.07, .11]	-.07 [-.17, .04]	-.02 [-.12, .08]						
Item 46	.00 [-.09, .10]	.17* [.09, .25]	.16* [.07, .24]	.12* [.03, .21]	-.06 [-.16, .04]					
Item 47	.04 [-.06, .14]	-.09* [-.17, .00]	-.10 [-.19, .00]	-.11* [-.21, -.02]	.05 [-.06, .15]	-.10* [-.19, -.01]				
Item 48	.15* [.06, .25]	-.05 [-.13, .04]	.01 [-.09, .10]	-.06 [-.15, .04]	.02 [-.08, .12]	.00 [-.09, .09]	.06 [-.04, .15]			
Item 49	-.05 [-.15, .05]	.00 [-.08, .09]	.15* [.06, .25]	.02 [-.08, .12]	-.10 [-.20, .00]	.05 [-.04, .14]	-.01 [-.10, .09]	.07 [-.03, .16]		
Item 50	.06 [-.04, .15]	.00 [-.08, .08]	-.02 [-.11, .07]	-.02 [-.11, .07]	.01 [-.08, .11]	-.01 [-.09, .07]	.01 [-.08, .10]	.07 [-.02, .15]	-.08 [-.17, .01]	

Note. 95% credible intervals are in square brackets. EC: emotional competence

*95% credible interval does not include zero.
