# Supplementary material for: Evaluating the higher-order structure of the Profile of Emotional Competence (PEC): Confirmatory factor analysis and Bayesian structural equation modeling
Source: PLoS One. 2019 Nov 14;14(11):e0225070. doi: 10.1371/journal.pone.0225070 (PMC6855477; doi:10.1371/journal.pone.0225070)
Supplement: S1 Text — (PDF) [file pone.0225070.s004.pdf]

S1 Text. Details and previous research applications of the hybrid structure model.

A bifactor model typically specifies that correlations among items or first-order factors can be accounted for by (a) a general factor (G-factor) representing shared variance among all the items' first-order factors and (b) a set of specific factors (S-factor) where variance over and above the general factor is shared among subsets of items or first-order factors [1, 2]. The S-factors are typically specified as uncorrelated (orthogonal) to one another or to the G-factor. Moreover, bifactor models can easily be expanded to include more than one G-factor, like the uncorrelated-trait and uncorrelated-method (UTUM) model for analyzing a multitrait, multimethod matrix [3, 4]. For example, Wu and Chen [4] applied this model to capture Elliot and Murayama [5]'s 2 (valence of competence: approach or avoidance) × 2 (definition of competence: mastery or performance) achievement goals. Furthermore, Caci, Morin [6] used a bifactor model with more than one G-factor for the Strengths and Difficulties Questionnaire [7], to capture 3 (board categories: strengths/prosocial behaviors, externalized disorders, or internalized disorders) × 4 (type of difficulty: conduct problems, hyperactivity-inattention, emotional symptoms, or peer problems) factor structure. Such a model is called a *hybrid model* [3].

References

1. Reise SP. The rediscovery of bifactor measurement models. Multivar Behav Res. 2012; 47:667–96. doi: 10.1080/00273171.2012.715555.

2. Rodriguez A, Reise SP, Haviland MG. Evaluating bifactor models: Calculating and interpreting statistical indices. Psychol Methods. 2016; 21: 137–50. doi: 10.1037/met0000045.

3. Howard JL, Gagné M, Morin AJS, Forest J. Using bifactor exploratory structural equation modeling to test for a continuum structure of motivation. J Manage. 2018; 44: 2638–64. doi: 10.1177/0149206316645653.
4. Wu C-H, Chen LH. Examining dual meanings of items in 2 × 2 Achievement Goal Questionnaires through MTMM modeling and MDS approach. Educ Psychol Meas. 2009; 70: 305–22. doi: 10.1177/0013164409344501.
5. Elliot AJ, Murayama K. On the measurement of achievement goals: Critique, illustration, and application. J Educ Psychol. 2008; 100: 613–28. doi: 10.1037/0022-0663.100.3.613.
6. Caci H, Morin AJ, Tran A. Investigation of a bifactor model of the Strengths and Difficulties Questionnaire. European Child & Adolescent Psychiatry. 2015; 24: 1291–301. doi: 10.1007/s00787-015-0679-3.
7. Goodman R. The Strengths and Difficulties Questionnaire: A research note. Journal of Child Psychology and Psychiatry. 1997; 38: 581–6. doi: 10.1111/j.1469-7610.1997.tb01545.x.
