# Supplementary material for: Evaluating the higher-order structure of the Profile of Emotional Competence (PEC): Confirmatory factor analysis and Bayesian structural equation modeling
Source: PLoS One. 2019 Nov 14;14(11):e0225070. doi: 10.1371/journal.pone.0225070 (PMC6855477; doi:10.1371/journal.pone.0225070)
Supplement: Attachment — Submitted filename: Respnses_to_Reviewers.docx [file pone.0225070.s005.docx]

**Responses to the Editor and Reviewers**

We would like to express our sincere gratitude to the editor and the reviewer for their insightful comments. Their comments help us to improve the paper significantly.

**Responses to the Editor**

**Comment 1:** *Please ensure that your manuscript meets PLOS ONE's style requirements, including those for file naming.*

**Response:** We have rechecked the guideline and ensured that our manuscript meets PLoS ONE’s style requirements.

**Comment 2:** *Please provide additional details regarding participant consent. In the Methods section, please ensure that you have specified (1) whether consent was informed and (2) what type you obtained (for instance, written or verbal). If your study included minors, state whether you obtained consent from parents or guardians. If the need for consent was waived by the ethics committee, please include this information.*

**Response:** In accordance with the comment, we have specified participant consent in the revised manuscript (lines 142–146).

**Responses to Reviewer 1**

**Comment 1:** *To explain my responses to the set questions, some of the code and data are in a private OSF page which I cannot currently access.*

**Response:** We apologize for this error. We have corrected it as suggested.

**Comment 2:** *My major concern with this manuscript is that there is a little conflation of measure and construct. The theoretical clarity of EI has often been questioned, and so when you suggest EI and EC are interchangeable terms on p2/3 I think this has more scope for causing confusion than clarity. For example, some researchers differentiate between cognitive ability (emotional intelligence), trait (affect-related personality) and behaviour (e.g. emotion regulation). It could be clearer exactly how you expect the competency perspective to fit in/around these other domains. This concern is furthered by the critique of competencies as atheoretical/ambiguous both within the EI domain, and much broader. The lack of theoretical position on competencies means there are some areas of ambiguity throughout the manuscript. For example, why would dimensions be excluded for not representing both ability and trait approaches – why would this matter if they were something different to these perspectives, or only related to one? As a result of these sorts of concerns, I think this paper would be much more valuable if it represented a discussion on the structure of the scale, rather than claiming for a definitive model of competencies.*

**Response:** Thank you for this thoughtful suggestion. As we explained in the manuscript, we use the term “emotional competence” rather than “emotional intelligence” because it can be improved via relatively short trainings and we operationalized the construct through the PEC. This is consistent with previous research using the PEC (e.g., Batselé, Stefaniak, & Fantini-Hauwel, 2019; Nozaki & Koyasu, 2016; Szczygiel & Mikolajczak, 2019). However, we agree that we should include a discussion on the structure of the scale, rather than claiming for a definitive model of competencies.

Thus, we have revised the Introduction section to be oriented toward the scale (i.e., Profile of Emotional Competence; PEC), rather than the construct (lines 43–125).

**Comment 3:** *Your detailing, presentation and analysis of the models looks pretty clear although some further details would be of benefit. For example, the justification for MLR over WLSMV is only convincing if you make the argument that the indicators provide additional value in model discrimination. I don’t have any experience with BSEM, although I am familiar with the principles, and so found some of the analysis section a little tricky to follow. My main problem with this is that it can be easy to miss errors or make subjective decisions which are difficult to follow. As such, I believe the analyses are robust but some of the more technical notes could be made a little easier to follow.*

**Response:** We have added an explanation that AIC and BIC are frequently used for model comparison (lines 181–183). Moreover, we have rechecked the contents of the analysis section while referring to reported information in previous research using BSEM (e.g., de Beer & Bianchi, in press).

**Comment 4:** *Your discussion is mostly clear, although I have two comments here. Firstly, the speculation for previous findings on lines 365-370 I would not encourage. Secondly, the fact that utilization-self did not load in the BSEM results is not a limitation of the current research- this finding should be discussed in the results or discussion section more substantively. You make an interesting line of argument around it not being include in factor structures of ability (e.g. see also Fan et al.) however again this links back to my first substantive comment about theoretical ambiguity.*

**Response:** In accordance with the comment, we have deleted lines 365–370 in the previous manuscript. Moreover, we have moved the discussion about utilization-self from the limitation section to the discussion section (lines 380–387).

Thank you again for your helpful and encouraging comments on the manuscript. We very much hope that you will find the revised manuscript suitable for publication.

**Responses to Reviewer 2**

**Comment 1:** *The study represents an important attempt to integrate previous findings into a model of Emotional Competences that goes beyond the traditional emotional intelligence paradigms. The authors utilize advanced statistical techniques to demonstrate that the five factor structure of Emotional Competences is relatively stable across cultures and show that the model distinguishing between intrapersonal and interpersonal domains fit the data best. Findings from this study can assist researchers and EI program developers who would like to base their implementations on a solid and evidence-based model of EC. There is tremendous value in investigating systematically the higher order structure of Emotional Competence in different cultures, and findings from this study would be very helpful for the design of future EI implementation programs on EI. The authors successfully introduce a complex theoretical distinction and clarify terminology issues, which have been a problem during decades of emotional intelligence research. The use of very relevant and up-to-date references is noticeable.*

**Response:** Thank you for your comment. We are grateful for your acknowledgment of the value of our research.

**Comment 2:** *At the end of the description of the PEC the authors refer to the fact that the modification of the Likert scale for one of their samples did not affect the main results. It would be helpful if they could clarify this sentence and indicate how they reached this conclusion. In addition, it would be advisable to incorporate examples of items in the PEC illustrating every component of the test.*

**Response:** We found similar factor structure across all samples (i.e., the modified target-based structure is best). Based on this result, we reached this conclusion. We have added this explanation in the revised manuscript (lines 165–167. Moreover, we have added example items of the PEC in the revised manuscript (lines 150–160).

**Comment 3:** *Some demographic variables seem to be quite different across samples (e.g., main age), as well as the questionnaire administration conditions (through data collection company online for the Japanese data only), so it would be advisable to identify clearly how the authors dealt with these differences.*

**Response:** As we explained in the manuscript, the main purpose of using four different samples is to test the stability and replicability of the results. Given that factor structure is usually same across different age or gender groups (e.g., Tsaousis & Kazi, 2013), we expect this difference will not significantly affect our results. We just tested whether the results were stable and replicable across samples and found that the modified target-based structure is best in all samples. Thus, no special method is need to dealt with differences in demographic variables in this study.

Furthermore, participants in all samples answered the questionnaire online. Thus, the questionnaire administration condition was similar across samples. We have clarified this point in the revised manuscript (line 142).

**Comment 4:** *The authors made great efforts in interpreting the results regarding interpersonal and intrapersonal EC. In order to further enhance this discussion, the authors could refer to how these findings could inform future implementation programs aiming to develop EC in particular, and Emotional Education in general (see Perez-Gonzalez & Qualter, 2018).*

**Response:** In accordance with the comment, we have added implication for EC training programs in the discussion section (line 358–367).

**Comment 5:** *As for the recommendations for future research, specific comments on future validation of the model could be made (content validity, discriminatory power, etc.). Also, the authors could elaborate on previous validation work using the PEC earlier on in the manuscript.*

**Response:** In accordance with the comment, we have suggested the need for future validation work in the discussion section (lines 393–395). Moreover, we have cited previous validation works that are using the PEC in the Introduction section (line 52–53).

Thank you again for your helpful comments on the manuscript. We hope that you will find the revised manuscript suitable for publication.
