# Supplementary material for: Evaluating the higher-order structure of the Profile of Emotional Competence (PEC): Confirmatory factor analysis and Bayesian structural equation modeling
Source: PLoS One. 2019 Nov 14;14(11):e0225070. doi: 10.1371/journal.pone.0225070 (PMC6855477; doi:10.1371/journal.pone.0225070)
Supplement: Attachment — Submitted filename: Respnses_to_Reviewers.docx [file pone.0225070.s006.docx]

**Responses to the Editor and Reviewers**

We would like to express our sincere gratitude to the editor and the reviewer for their insightful comments. Their comments help us to improve the paper significantly.

**Responses to Reviewer 1**

**Comment 1:** *You have done a good job at reframing the work to be about the scale rather than the construct, however your introduction still presents a slightly confused review of EI theory. For example, you conflate EC with trait EI (p3) – particularly in the second paragraph (starting “Research conducted over the last two decades…”) where you cite mostly trait EI research, however some may argue that your central argument that EC is malleable to training is much less true for trait EI. Furthermore, this becomes slightly further confused when suggesting the PEC is built upon the*

*Mayer and Salovey model (p4) which itself is an ability EI framework. I would recommend you take a clearer stance on this – this could be done in a number of different ways – you could argue that EC is different to ability EI and trait EI, you could argue that trait EI and EC are in essence the same, you could argue that competencies are the behavioural outcomes of ability EI (hence the Mayer and Salovey framework) and trait EI (hence self-report behavioural measure), or take it in a different way. Whichever way, framing it more clearly and acknowledging the consequences of this decision is vital. As it stands this is the major obstacle to publication for this work is this theoretical clarity which frames the whole paper.*

**Response:** A recent meta-analysis shows that relatively short trainings can improve all EI based on the ability model, the trait model, and the mixed model, unlike cognitive intelligence (Hodzic et al., 2018). Given this line of research, we think emotional competence (EC) is a more suitable term to refer to these individual differences than emotional intelligence (EI). This is why we use the term EC instead of EI in the manuscript. We have added this explanation in the main text (lines 22–27). Moreover, to increase the theoretical clarity, we have also added the explanation of the tripartite model proposed by Mikolajczak et al. (2009) to clarify how different levels of EC/EI (knowledge, abilities and traits) are related to each other (lines 31–40) and what the terms “EC” used here refers to.

**Comment 2:** *Remove ‘emotional competence using’ from the title*

**Response:** In accordance with the comment, we have removed them from the title.

**Comment 3:** *P3. Trait EI… is TYPICALLY measured using self-report. It’s worth adding this conditional statement as there has been a body of research considering other-rated trait EI/competencies*

**Response:** In accordance with the comment, we have added “typically” to that sentence (line 41).

**Comment 4:** *P13 there are two ‘that’ in the sentence – that that*

**Response:** We have fixed this mistake (lines 236).

**Comment 5:** *P13 your lack of convergence sounds like a Heywood case. Is that the case and if so did you attempt any resolution?*

**Response:** With regards to the competence-based structure and modified competence-based structure, it is a Heywood case, because some correlation coefficients among second-order factors exceeded 1.00 (see S1 Table). We might try to fix this type of issue by using inequality constraint options (set correlations < 1.00) during estimation). However, this procedure is not recommended in the previous literature because these ‘‘fixes’’ obscure important sources of model misspecification (Fan & Lance, 2017; Kline, 2010). Rather, previous literature suggests that we should try to inspect the source of the problem and interpret them (Kline, 2010). As the S1 Table clearly shows, many correlation coefficients among second-order factors exceeded 1.00. Thus, instead of unreasonable attempts to fix these issues, we interpreted the source of the improper solution and conclude that the factors were overextracted in the competence-based structure and modified competence-based structure.

**Comment 6:** *P26 you link the findings to emotional education as requested by the second reviewer – I would encourage you to expand on this just a little more in context of the theoretical approach adopted. i.e. if you take a trait or competency approach, what is the evidence that such individual differences can be trained, and what would you expect to improve from understanding the factor structure differentiating between interpersonal and intrapersonal?*

**Response:** We have added recent findings showing that trait-level EC can be improved through training programs and discussed the value of intra- and interpersonal dimensions for emotional education (lines 365–375).

**Comment 7:** *P27 you discuss the utilisation branch and acknowledge in other factor analytic work that this does not emerge. Could you provide just a little more detail about your findings in this area i.e. do loadings or relationships to other factors seem higher than the others etc.*

**Response:** We have added this information to the revised manuscript (lines 392–393).

Thank you again for your helpful and encouraging comments on the manuscript. We very much hope that you will find the revised manuscript suitable for publication.

**Responses to Reviewer 2**

**Comment 1:** *The authors have successfully answered all the comments raised and I recommend this manuscript for publication in PLOSONE.*

**Response:** Thank you for your comment. We are grateful for your acknowledgment of the value of our research.
